# Assignment of the *Q*-Bands of the Chlorophylls: Coherence Loss via Q_x_ − Q_y_ Mixing

**DOI:** 10.1038/srep02761

**Published:** 2013-09-26

**Authors:** Jeffrey R. Reimers, Zheng-Li Cai, Rika Kobayashi, Margus Rätsep, Arvi Freiberg, Elmars Krausz

**Affiliations:** 1 School of Chemistry, The University of Sydney, 2006, NSW, Australia; 2 Australian National University Supercomputer Facility, Mills Rd, Canberra, ACT 0200, Australia; 3 Institute of Physics, University of Tartu, Riia 142, 51014 Tartu, Estonia; 4 Institute of Molecular and Cell Biology, University of Tartu, Riia 23, 51010 Tartu, Estonia; 5 Research School of Chemistry, The Australian National University, Canberra 2601, Australia

## Abstract

We provide a new and definitive spectral assignment for the absorption, emission, high-resolution fluorescence excitation, linear dichroism, and/or magnetic circular dichroism spectra of 32 chlorophyllides in various environments. This encompases all data used to justify previous assignments and provides a simple interpretation of unexplained complex decoherence phenomena associated with Q_x_ → Q_y_ relaxation. Whilst most chlorophylls conform to the Gouterman model and display two independent transitions Q_x_ (S_2_) and Q_y_ (S_1_), strong vibronic coupling *inseparably mixes* these states in chlorophyll-a. This spreads *x*-polarized absorption intensity over the *entire* *Q*-band system to influence all exciton-transport, relaxation and coherence properties of chlorophyll-based photosystems. The fraction of the total absorption intensity attributed to Q_x_ ranges between 7% and 33%, depending on chlorophyllide and coordination, and is between 10% and 25% for chlorophyll-a. CAM-B3LYP density-functional-theory calculations of the band origins, relative intensities, vibrational Huang-Rhys factors, and vibronic coupling strengths fully support this new assignment.

All exciton-transport and quantum coherence aspects of photosynthesis^1^,^2^,^3^,^4^,^5^,^6^ are controlled by the properties of the *Q*-band of chlorophyllides. The Gouterman model^7^ describes this band as comprising two (possibly overlapping) independent electronic transitions named Q_x_ (or S_2_) and Q_y_ (or S_1_) after their polarization directions within the macrocycle plane. Each transition in absorption involves a sharp major peak (the band origin) followed at slightly higher energy by extensive vibrational structure that coalesces at low resolution into a wide low-intensity broad sideband. Many experimental techniques such as absorption (ABS), emission (EMI), polarized emission, linear dichroism (LD), circular dichroism (CD), and magnetic circular dichroism (MCD) have been applied to assign the key spectral features and their changes as a function of chemical composition and solvation environment. We obtain a consistent spectral assignment covering a range of environments for 32 chlorophyllide and related macrocycles: (chlorophylls (Chl) a, b, c1, c2, c3, d; pheophytins (Pheo) a, b, d; bacteriochlorophylls (BChl) a, b, c, d, e, f, g; bacteriopheophytins (BPheo) a, b, g; the metal-substituted Chl-a derivatives Co(II)-Chl-a, Cu(II)-Chl-a, Ni(II)-Chl-a, Zn(II)-Chl-a; tetrameso(di-*t*-butylphenyl)porphyrin; protochlorophyll-a; protopheophytin-a; chlorin-e_6_ trimethyl ester; rhodochlorin dimethyl ester; isochlorin-e_5_ dimethyl ether; methylpheophorbide-a; pyromethylpheophorbide-a; and mesopyromethylpheophorbide-a (see Supplementary Information (SI) Sect. S1).

We focus primarily on ABS and MCD data owing to the wealth of available information collected during the last 50 years, with spectra for 12 critical chlorophyllide/solvent combinations shown in Fig. 1 and data for another 17 combinations shown in SI Sect. S4. Note that Fig. 1 includes analysis of ChlZ(D1) *in situ* in Photosystem-II, deduced from the spectral differences observed when this Chl is deleted from the protein^8^,^9^. All ABS spectra are shown in the form *A*(Δ*ν*)/*ν* as a function of the frequency change Δ*ν* from the intense Q_y_ band origin as this "band shape" function is independent of the actual origin location, unlike spectra plotted as a function of wavelength. Similarly, the MCD spectra are plotted as ΔΔ*A*(Δ*ν*)/*ν* where Δ*A* is the natural CD spectrum and ΔΔ*A* is it's variation with applied magnetic field. Individual spectra arising from the Q_x_ and Q_y_ states simply add to give the total ABS spectrum but in MCD the Q_y_ band gives rise to a positive signal whilst the Q_x_ band gives rise to a negative one, allowing polarization information to be extracted from combined ABS and MCD data sets; an important feature, however, is that the magnitude of the ratios of the MCD to ABS contributions from each state differ significantly.

Figs. 1(a)–(e) show some well-known and well-understood spectra for chlorophyllides like BChl-a and Pheo-a for which the energy gap Δ*E* between the Q_x_ and Q_y_ origins is large, of the order 3000–4500 cm^−1^ 10,11. The critical features of the Gouterman model are immediately apparent, including the locations of the Q_y_ and Q_x_ origins, which are indicated on the figure by arrows, and the extensive but weak sideband structure. The origin transitions are broadened by inhomogeneous effects and by the activation of low-frequency molecular vibrations and intermolecular phonons, but can be viewed as single transitions. However, excitation of the high-energy vibrational sidebands results in considerable excited-state dynamics, with the excess energy rapidly being distributed incoherently into the surroundings. This limits coherence effects in photosynthesis.

Most significantly, the spectra of Chl-a shown in Figs. 1f, 1i, and 1j are strikingly different to those of say BChl-a and Pheo-a, displaying *two* *x*-polarized absorptions that naively both look like band origins, with the relative intensity of the bands being strongly solvent dependent. An important feature in common, however, is that the energy gap Δ*E* significantly reduces from that in solvents like ether with 5-coordinate magnesium (5CO) compared to that in solvents like alcohols and pyridine which induce 6-fold coordination (6CO). As is emphasised in Fig. 2, for Chl-a, one of the two *x*-polarized bands is located quite close to the Q_y_ origin at Δ*E* = ~700 cm^−1^ (6CO, in pyridine) or ~1100 cm^−1^ (5CO, in ether), with the other band being much further removed at Δ*E* = ~1700 cm^−1^ (6CO) or ~2100 cm^−1^ (5CO)^11^,^12^. The nature of the processes that give rise to these sub-bands, and the fraction of the absorption attributable to each, will significantly influence exciton transport and photosynthetic function. Any interaction between the Q_y_ state and these *x*-polarized features will redistribute energy flow and facilitate decoherence. For over 50 years, the quest to assign the Q-band spectrum of Chl-a has sought answers to these fundamental questions concerning the operation of natural photosynthesis, as well as to provide principles for the design of artificial devices.

Historically, two different assignments of the spectra of Chl-a have been proposed: the "traditional" 1960's assignment identifies the higher-energy component as the Q_x_ origin^10^,^13^,^14^,^15^,^16^, while the "modern" 1980's assignment selects the lower-energy one, see Fig. 2. The traditional assignment is strongly supported by the observed^17^ asymmetry between ABS and reflected EMI spectra: as emission occurs from only Q_y_, a significant reduction of emission observed at 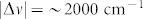 is taken to identify the Q_x_ origin in this region. However, Rebane and Avarmaa^18^,^19^,^20^ measured high-resolution polarized fluorescence excitation (FE) spectra of Chl-a in ether at 4.5 K and concluded that the lower band must be the Q_x_ origin. Over the next 20 years, improved LD spectra^21^ and quantitative MCD studies^22^,^23^,^24^ on Chl-a and its closely related variants BChl-c and BChl-d (see e.g. Fig. 1) were interpreted to support the "modern" assignment over the "traditional" one.

If the "traditional" assignments were correct, then the Q_x_ state would be too far removed from Q_y_ to significantly influence decoherence, exciton transport and photosynthetic function. Indeed, all previous studies of exciton transport have ignored possible effects of Q_x_, effectively adopting this assignment^2^,^25^,^26^,^27^. While profound consequences could arise based on the "modern" assignment, their influence must be scaled by the fraction *f_x_* of the absorption attributed to Q_x_. No such estimate has ever been made as this requires understanding of the origin of *both* of the observed *x*-polarized band components. Hence, to date it has not been possible to go beyond the traditional assignment when considering photosynthetic function.

The essential problem is that the Gouterman model predicts one *x*-polarized band^14^, and both the "traditional" and "modern" assignments fail to qualitatively account for the observation of two bands. Very recently, our own research has brought this issue into focus, contradictorily concluding that: (i) spectral hole-burning (HB) experiments on photosystems give results that seem to be consistent only with the "traditional" assignment^27^, (ii) high-resolution change in fluorescence line narrowing (ΔFLN) experiments support the "modern" assignment^28^, and (iii) *neither* assignment leads to Δ*E* values in agreement with CAM-B3LYP time-dependent density-functional theory (TD-DFT). A global solution is required that accounts for *all* spectral features of *all* chlorophyllides in *all* solvation environments.

Many of these issues were indeed known to Gouterman 50 years ago and he and later workers considered possible solutions including: the involvement of a third electronic state, Franck-Condon progressions, and Q_x_ − Q_y_ vibronic coupling^10^,^14^,^18^,^19^,^20^. Franck-Condon effects can be quickly discounted as if 0 ← 0 and 1 ← 0 transitions show significant intensity then 2 ← 0 should be observable and it is not. The presence of a third electronic state was indicated by TD-DFT calculations^29^,^30^ which placed another transition in the *Q*-band region. However, we have shown that the identified state was in fact a misrepresented, high-energy *N*-band of Chl-a^31^, a type of band whose properties are incorrectly described by the density-functionals used in those studies^32^. Similar predictions made by these methods for porphyrins were found to be incorrect^32^. Also, we showed that these TD-DFT methods incorrectly predict the *N*-band to become the lowest-energy state in PS-I^33^. Modern density functionals such as CAM-B3LYP^34^, as well as *ab initio* coupled-cluster calculations, place the *N*-bands at far higher energy^31^. Vibronic coupling thus remains the only viable possibility to explain the appearance of two *x*-polarized bands for Chl-a^27^,^33^.

Previously, we postulated that the inclusion of vibronic coupling is essential to even a *qualitative* understanding of the spectroscopy of Chl-a^27^,^33^. Here, we present a quantitative vibronic-coupling^35^ model that accounts for *all* observed *Q*-band phenomena and apply it to interpret the spectroscopic properties of 32 chlorophyllides (and related molecules) in a wide range of solvents and photosystems. While the previous assignments of the spectrum of chlorophyll evoked the basic Gouterman model^7^ which treats Q_x_ and Q_y_ as independent transitions that can be slightly perturbed by effects such as vibronic coupling, our new assignment sees the two transitions as being *inseparably mixed*. This mixing, and how it generates two separate *x*-polarized bands that are well removed from the unperturbed Q_x_ origin, are illustrated in Fig. 2. Our analysis is directly based on quantitative descriptions for such a scenario as originally proposed by Gouterman^16^,^36^. The critical results of this new assignment, which display a surprisingly strong dependence of the Q_x_ intensity on chlorophyllide and environment, are then shown to be in excellent quantitative agreement with CAM-B3LYP TD-DFT calculations that were performed 5–7 years ago but found to be inconsistent with proposed assignments. In the Discussion we use our assignment to understand the decoherence processes associated with Q_x_ → Q_y_ relaxation, a complex phenomenon with no current qualitative explanation, showing how this process correlates with general chemical kinetics schemes.

## Results

### Absorption and magnetic circular dichroism spectral analysis

The Q_y_ and Q_x_ transitions arise owing to two different electronic excitations within chlorophyllides, facilitating absorption and emission of radiation in orthogonal directions^14^. As the ground-states of the molecules have different equilibrium geometries to these excited states, electronic excitation induces vibrational motion, adding vibrational sidebands to the electronic band origins. High-resolution hole-burning (HB) spectroscopy can determine the Huang-Rhys factors that specify the Franck-Condon-allowed relative contribution of each molecular vibrational motion to this process. However, as argued in SI Sect. S3, HB data is insufficiently precise to sustain a full quantitative spectral analysis of chlorophyllide properties. Instead, we develop a new method for extracting the Huang-Rhys factors that analyses high resolution FE data combined with low-resolution spectral band-contour data. FE provides a precise measurement of relative Huang-Rhys factors but cannot determine their absolute scale factor, a feature that is actually easily determined from low-resolution spectra. Fig. 3 shows the observed^20^ high-resolution FE spectrum of Chl-a in ether (black) at 4.2 K and the almost indistinguishable fit to the sum of 236 vibrational-mode excitations (red) and other contributions. The total Huang-Rhys factor is 0.278 whilst the total reorganization energy is 262 cm^−1^ (details are given in SI Sect. S2). Important aspects of this process are the full inclusion of intrinsic zero-phonon-line (ZPL) and phonon side-band (PSB) linewidth profiles (see Fig. 3 insert) as well as full inclusion of multi-quanta excitations (this contribution is shown in blue in Fig. 3). However, the most critical feature of the fitting process is the specification of the intensity attributed to Q_x_. This step is done self-consistently along with the subsequently described fitting of the ABS and MCD spectra: an initial Q_x_ spectrum is assumed and used to generate the Q_y_ Huang-Rhys factors from the FE data, and then these factors are used to fit a new Q_x_ spectrum to the MCD data, and the process is cycled until the extracted component spectra no longer change.

Fig. 4a shows the Franck-Condon allowed component of the FE spectrum reconstructed from these Huang-Rhys factors and ZPL profile where it is compared to analogous spectra simulated using CAM-B3LYP/6-31G* calculated factors (see SI Sect. S6). Excellent agreement between such observed and calculated high-resolution data has already been described for BChl-a, a molecule with a large Q_y_ − Q_x_ gap for which the experimental data is simple to interpret^37^, and Fig. 4a demonstrates that agreement (at least to low resolution) is also obtained from our (complex) analysis of Chl-a.

We apply the relative Huang-Rhys factors fitted to the Chl-a data to all chlorophyllides, noting that the often significant observed changes in the shape and intensity of the vibrational sidebands are usually well represented by simply rescaling the total Huang-Rhys factor. A single numerical factor per chlorophyllide and environment is thus used to fit many spectral changes. Also, as no high-resolution data is available for the Huang-Rhys factors of the Q_x_ state, we assume that these are the same as those determined for Q_y_. This crude assumption is adequate for our purpose as the vibrational sidebands of Q_x_ usually appear outside of the critical energy range required to assign spectra. Similarly, we also neglect the effects of Q_x_ − B_y_ vibronic coupling in this work.

Critical, however, is the mixing of Q_x_ character into the Q_y_ state (and vice versa) through vibronic coupling, which confuses the identities of these two states. In practice what this means is that a vibrational sideband of the Q_y_ state can absorb (or emit) in *both* (*y*) and (*x*) polarization directions. When the forbidden (*x*) absorption becomes comparable with the Franck-Condon allowed absorption, the Q_y_ and Q_x_ states become intrinsically mixed and cannot be considered to be within the Born-Oppenheimer approximation.

Unfortunately current experimental data do not provide high-resolution information regarding the nature and form of the vibrational motions that mix Q_x_ and Q_y_. As CAM-B3LYP/6-31G* calculations qualitatively reproduce the experimental data for the Franck-Condon allowed spectrum (Fig. 4a), they are expected to provide useful information concerning the vibronic coupling as well. Results for Chl-a (see SI Sect S6) are shown in Fig. 4b, predicting that a single mode at a *coupling-modified* frequency of ca. 1100 cm^−1^ in Q_y_ dominates the coupling. Hence we use a one-mode model (see SI Sect. S4) to fit the observed ABS and MCD spectra of Chl-a, fitting the *unperturbed* frequency of this mode in the ground state at *ν_vc_* = 1500 cm^−1^ with coupling constant of *α* = 750 cm^−1^. The small energy gap between this unperturbed Q_y_ vibrational line and Q_x_ origin for Chl-a is sketched in Fig. 2. We apply these parameters universally to every chlorophyllide in each solvation environment, ignoring any chemical variations, as our aim here is to depict the qualitative spectral features of all chlorophyllides rather than to focus on say how chemical variations quantitatively control photosynthesis. Similarly, use of multi-mode models will improve quantitative accuracy but not change the key qualitative features.

Previous quantitative analyses of the MCD spectra of chlorophyllides have taken the approach of fitting the observed ABS spectra to sums of Gaussian functions. Typically, 7 Gaussians are used to describe the Q-band origins and dominant side-band features^22^,^23^,^24^. For each Gaussian, 4 parameters are involved: the centre, height, and width of each function plus a scaling factor which relates the MCD intensity to the ABS intensity, giving a total of 28 parameters. Such an analysis is incapable of detecting a weak band of one polarization that coincides with a strong band of the other. All it yields is a reduced relative MCD scale factor for the stronger band. Later we show that minor and seemingly unimportant spectral features for molecules like BChl-a and Pheo-a, not detected by Gaussian analysis, manifest immediately when our vibronic-coupling model is applied; these features turn out to be critical to the global analysis of chlorophyllides.

Our alternative approach involves 9 adjustable parameters used to fit the combined ABS and MCD spectra for each chlorophyllide (see SI Sect. S4). Two of these parameters are unimportant frequency-shift and absorption-strength scaling factors, making just 7 non-trivial parameters: the energy gap Δ*E*, the relative fraction *f_x_* of the absorption with *x* polarization, inhomogeneous line widths for each state, *single* MCD scale factors for each state, and the total Huang-Rhys factor *S* used sometimes to rescale the Franck-Condon factors compared to Chl-a in ether. All parameters thus have clear physical meaning and provide concise quantitative analysis of the observed spectra.

**Figure 1:**
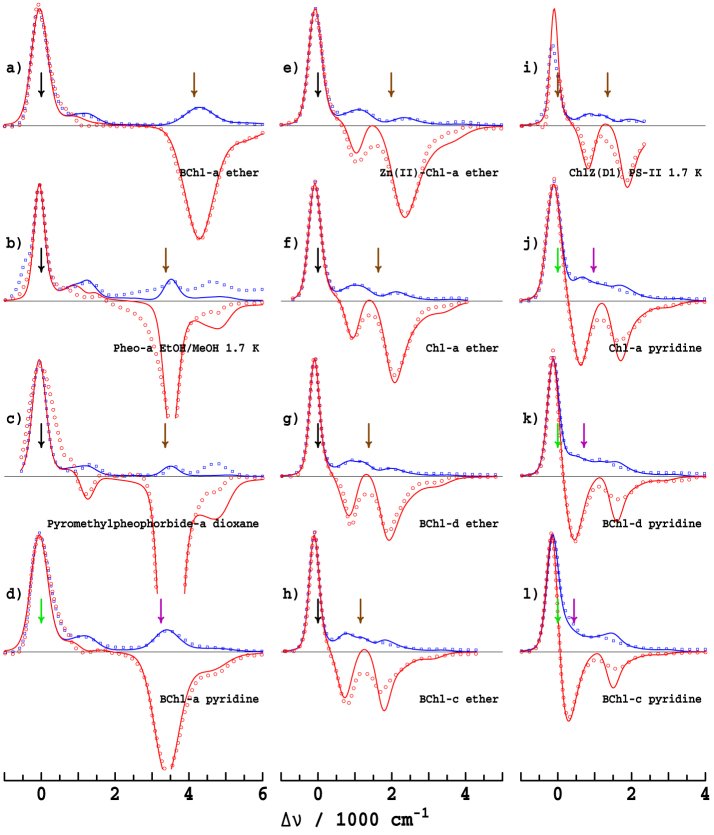
Observed ABS (*A*/*ν*, blue dots) and MCD (ΔΔ*A*/*ν*, red dots) spectra and their fits (lines) obtained using a vibronic coupling model with *ν_vc_* = 1500 cm^−1^ and α = 750 cm^−1^, see SI Sect. S4. Solvents are as indicated, measurements were made at room temperature unless otherwise noted. Unperturbed electronic origins are indicated by arrows: black- free-base or 5CO Q_y_, brown- free-base or 5CO Q_x_, green- 6CO Q_y_, purple- 6CO Q_x_. All spectra are broadened using a Gaussian function of HWHM = 47 cm^−1^ to reduce noise, obtained from: a,d-Umetsu^24^; f,h,j,l- Umetsu^22^; c- Briat^49^; b- Razeghifard^50^; e- Nonomura^25^; i- Krausz^8^,^9^; g,k- Umetsu^23^.

**Figure 2:**
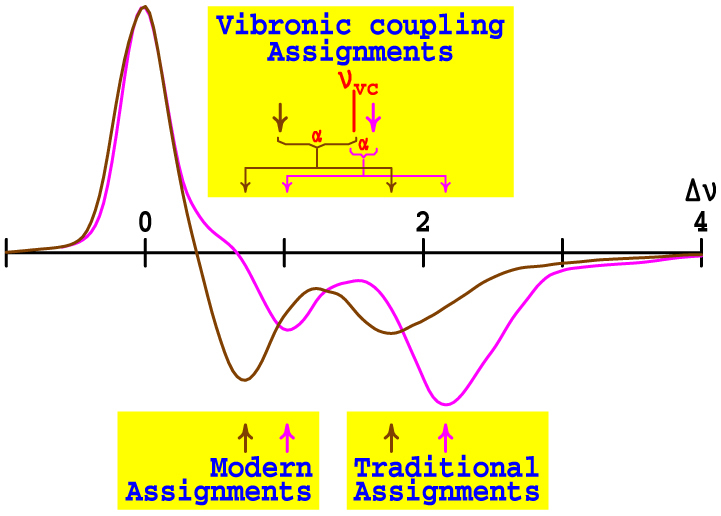
The traditional, modern, and vibronic coupling assignments of the Q_x_ origin from the MCD spectra^22^ (relative ΔΔ*A*/*ν* vs. Δ*ν* in 1000 cm^−1^) of Chl-a in ether (magenta) and pyridine (brown); *ν_vc_* is the unperturbed frequency of the coupling vibration in the Q_y_ state while *α* is the coupling between this vibration and Q_x_.

**Figure 3:**
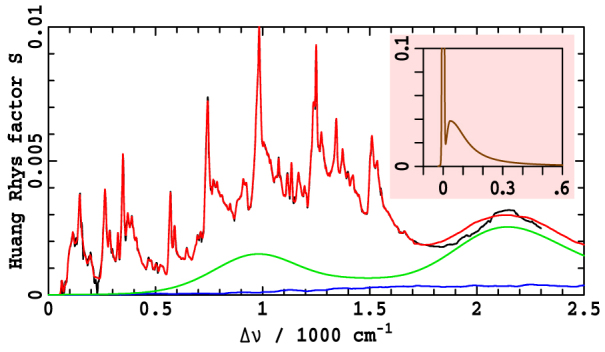
The normalized high-resolution FE spectrum^20^ of Chl-a in ether at 4.2 K, measured at 660 nm, is shown in black after subtraction of the estimated zero-phonon line and phonon side-band spectrum (insert). This is then fitted (red) to the sum of a low-resolution *x*-polarized component (purple, obtained self-consistently along with the MCD spectral fit) and a 236-mode Franck-Condon allowed component of total *S* = 0.278; the blue curve shows the contribution of multiple excitations to this Franck-Condon spectrum. Full details are in SI Sect. S2.

Results of our simultaneous fit of the ABS and MCD spectra of 12 chlorophyllide/solvent combinations are summarized in Fig. 1, with the spectra of each individual component and results for a further 18 more combinations given in SI Sects. S9 and S4, respectively; values of Δ*E* and *f_x_* are provided in Table 1 for the most important systems. As the figures show, all significant features are semi-quantitatively accommodated by this fitting procedure, including the appearance of *two* intense *x*-polarized bands for Chl-a, BChl-c, BChl-d, etc.

A critical feature of vibronic coupling is that its manifestations are controlled by the energy gap Δ*E* which, from Table 1, is found to range from −2420 cm^−1^ for a porphyrin to 4140 cm^−1^ for BChl-a in ether. Another critical feature identified is that the fraction of *x*-polarized absorption varies from 0.07 for chlorin-e6 in dioxane and Ni(II)-Chl-a in ether to 0.33 for the porphyrin. While this fraction controls the magnitude of the *x*-polarized signal, its appearance as either a dominant single band (as in porphyrin, BChl-a and Pheo-a) or as two separate bands (as in Chl-a), is controlled by the energy gap.

Consider first the spectra for BChl-a in ether shown in Fig. 1a. Vibronic coupling mixes the active vibrational mode of the Q_y_ state at Δ*ν* = *ν_vc_* = 1500 cm^−1^ with the Q_x_ origin at Δ*ν* = Δ*E*/*h* = 4140 cm^−1^ (Table 1). Rayleigh-Schrodinger perturbation theory indicates that this interaction will depress the frequency of the vibronically active mode of Q_y_ by 2*α*^2^/(Δ*E* − *hν_vc_*) = 420 cm^−1^ to ca. 1100 cm^−1^ and apportion it a fraction 2*α*^2^/(Δ*E* − *hν_vc_*)^2^ = 0.16 of the total Q_x_ intensity. While this intensity is quite small, it is clear from the figure that the observed MCD bandshape at Δ*ν* = 1100 cm^−1^ is depressed relative to the ABS bandshape, just as the vibronic coupling model predicts. Such critical *x*-polarized absorption is not identifiable using Gaussian fitting procedures^24^. The spectra in Fig. 1 are ordered by decreasing Δ*E*, revealing a continual intensification of the *x*-polarized Q_y_ vibrational band as the gap gets smaller; this effect is seen for *all* chlorophyllides in *all* solvents including many examples like pyromethylpheophorbide-a (Fig. 1c) for which the *x*-polarized component of the Q_y_ transition is quite clear from inspection of the raw data.

**Figure 4:**
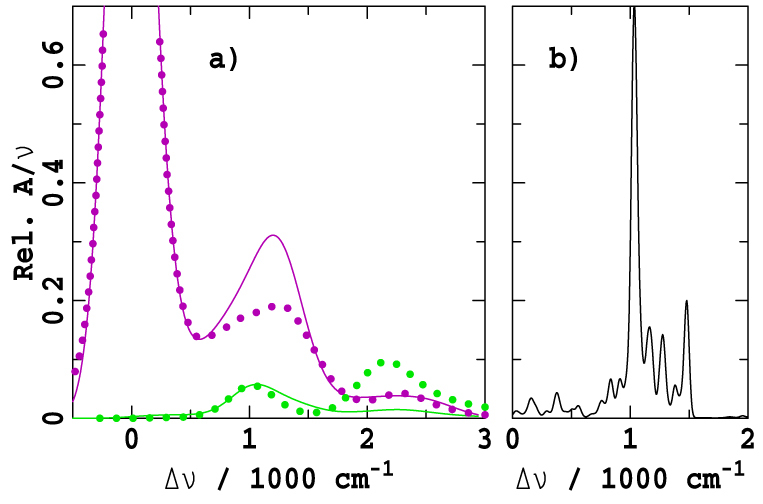
Comparison of observed and CAM-B3LYP/6-31G* spectral properties (see SI Sect. S6) of Chl-a: (a) Observed Q_x_ + Q_y_ (dots) and CAM-B3LYP Q_y_ (lines) absorption components for the Franck-Condon *y*-polarized (purple) and Herzberg-Teller *x*-polarized (green); with (b) showing the calculated distribution of vibronic-coupling strengths.

Consider now the spectra for Chl-a in ether, Fig. 1f. The bandgap is reduced to Δ*E* = 1640 cm^−1^ (Table 1) so that the Q_x_ origin becomes nearly resonant with the vibronically active mode of Q_y_ at Δ*ν* = *ν_vc_* = 1500 cm^−1^. As a result, the *x*-polarized intensity is split nearly equally between two components located at Δ*ν* ≈ *ν_vc_* ± 2^−1/2^*α*/*h* = 970 cm^−1^ and 2030 cm^−1^. Hence *both* observed spectral features originate from the Q_x_ origin, and the *x*-polarized intensity is distributed over the entire Q-band system. Fig. 4a compares the fitted *x*-polarized absorption component to one obtained using simplistic CAM-B3LYP/6-31G* calculations (see SI Sect. S6). These calculations determine the vibronic coupling constants for each individual mode (as shown in Fig. 4b) and then use perturbation theory to calculate the spectrum, ignoring the effects of resonance. Good agreement is seen between the fitted and calculated band structures in the important region near Δ*ν* = 1000 cm^−1^, indicating strong qualitative support for the experimental assignment. Resonance is ignored in these calculations and therefore the second *x*-polarized band at Δ*ν* = 2200 cm^−1^ cannot be reproduced.

Fig. 1 also shows that when Δ*E* falls below 1500 cm^−1^, as for Chl-a in pyridine or alcohols and for BChl-c and BChl-d, the lower-energy component dominates the *x*-polarized bandshape. This occurs as the resonance becomes weakened and the lower-energy component retains the primary character of the unperturbed Q_x_ origin. In porphyrins and some molecules like Chl-c, Q_x_ becomes the lowest-energy transition, see SI Sect. S7.

### Resolution of some cornerstone experiments

**Figure 5:**
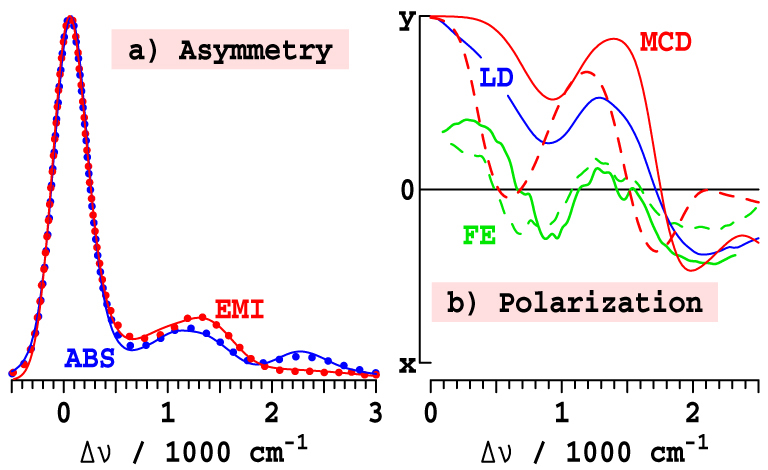
Comparison of observed data with interpretation for Chl-a in ether. (a) Asymmetry between the 295 K ABS *A*(Δ*ν*)/*ν* and reflected EMI *E*(−Δ*ν*)/*ν*^3^ spectra from experiment^28^ (dots) and the vibronic-coupling model fits to the MCD data (lines, see SI Sect. S4) with *S* increased by 50% to 0.417 for emission. (b) Linear polarization (5CO solid lines, 6CO dashed lines) from LD Fragata^21^ (after correction for the observed 20° nonorthogonality of the Q_x_ and Q_y_ polarizations, see SI Sect. S5), from polarized FE^19^ obtained at 665 nm (largely 6CO) and 675 nm (mixed) fluorescence recording, and from the MCD fit and analytical inversion assuming maximal Q_x_ intensity.

The "traditional" assignment was originally advanced based on the relative strengths of the two *x*-polarized bands for Chl-a in 5CO solvents like ether, but the subsequent demonstrations that the relative intensities could invert by changing the solvent or chlorophyllide invalidated this argument^22^,^23^,^24^. Another significant diagnostic was the observed asymmetry between ABS and reflected EMI spectra. As shown in Fig. 5a (see SI. Sect. S6) for Chl-a in ether, the ABS spectra is much more intense in the critical 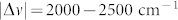 region, suggesting this as the location of Q_x_. However, the EMI intensity predicted based on the fit to the ABS and MCD data, shown also in the figure, accurately reproduces the observed data.

The "modern" assignment was originally advanced based on polarized FE data^19^, later supported by LD^21^ and MCD^22^,^23^,^24^ polarization information. The LD and FE polarizations, re-expressed on a similar scale (see SI Sect. S5), are compared to our MCD polarization in Fig. 5b. Given the significant difficulties associated with the LD and polarized FE measurement interpretations, it is clear that these data sets are in good qualitative agreement.

Finally we note that another critical experimental indicator used to support the "modern" assignment is its ability to predict the observed ratio "*B*/*D*" depicting the relative sensitivities of MCD and ABS Q_y_ spectra as a function of chlorophyllide and solvent^22^,^23^,^24^. This data is discussed in detail in SI Sect S4 where it is demonstrated that our revised assignment also provides an excellent description. Further, we show that our assignment allows this analysis to be *extended* to also describe the observed relative sensitivities for Q_x_, providing complete fundamental understanding for the origin of the MCD effect.

### Comparison of assignments with TD-DFT calculations

**Figure 6:**
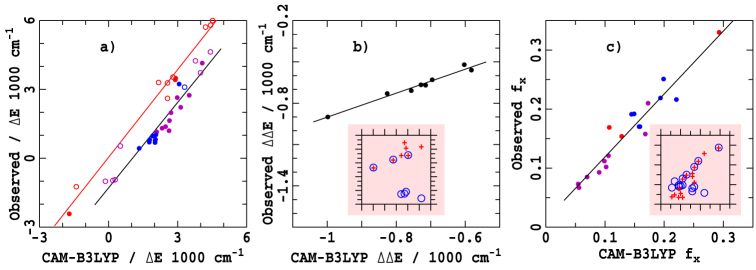
Comparison of observed and CAM-B3LYP/6-31G* Q-band properties for solvated chlorophyllides (see SI Sect. S7): filled circles- vibronic-model fit, open circles- raw observed peak locations; red- free-base molecules, blue- 5CO, purple- 6CO, black 5CO-6CO difference, for (a) unperturbed Q_x_ − Q_y_ gaps Δ*E*, (b) their changes ΔΔ*E* in going from 5CO to 6CO, and c) corresponding observed and calculated fraction *f_x_* of the *Q*-band absorption attributed to Q_x_. The inserts in (b) and (c) show the lack of correlation between calculation and experiment using the "traditional" (blue circles) or "modern" (red crosses) assignments.

In Fig. 6a, the unperturbed gaps Δ*E* extracted for 34 chlorophyllide/solvent systems using the vibronic-coupling model are compared to CAM-B3LYP TDDFT calculated values for chlorophyllide-solvent complexes (see SI Sect. S7). In general good agreement is found, but it is clear that the CAM-B3LYP calculations systematically overestimates Δ*E* by 1000 cm^−1^ for free-base molecules compared to metalated ones. A property that is less sensitive to shortcomings in the computational method is the energy change ΔΔ*E* on going from 5CO to 6CO. Calculated and observed values of ΔΔ*E* are plotted in Fig. 6b. Excellent agreement is obtained using the new vibronic-coupling assignment whereas the "traditional" and "modern" assignments (figure inserts) do not correlate with the calculations. Similarly, calculated and observed values for the fraction of absorption attributed to Q_x_, *f_x_*, plotted in Fig. 6c reveal that only the vibronic-coupling assignment is consistent with the calculations. Most significantly, the calculations verify the unanticipated experimental identification of a 5-fold variation in *f_x_* with chlorophyllide and coordination environment. The calculations were in fact performed 5-7 years ago but were irreconcilable with the data at that time.

## Discussion

Our vibronic-coupling model treats the Franck-Condon active modes in full high resolution but includes only a single model mode to depict the vibronic coupling. Clearly, experimental techniques need to be determined to measure high-resolution spectra of the vibronically active modes so that this model can be significantly improved. Also, individual treatments of the vibronic coupling strengths and mode frequencies for each chlorophyllide and environment are warranted.

As Q_x_ is shown to be responsible for a significant fraction of the absorption across the whole *Q* band of Chl-a, the presented model warrants its immediate use in all calculations of exciton transfer and quantum coherence in Chl-a and related photosystems, but it will require enhancement either by empirical modelling or via use of calculated data such as that shown in Fig. 4b. In particular, an empirical representation of the missing information in the single vibronic-mode model offers the possibility of rapid numerical evaluations or even analytical descriptions of complex photosystem phenomena. Here we develop one example of empirical enhancement to understand complex and unexplained decoherence processes associated with the Q_x_ → Q_y_ relaxation of chlorophyllides.

**Figure 7:**
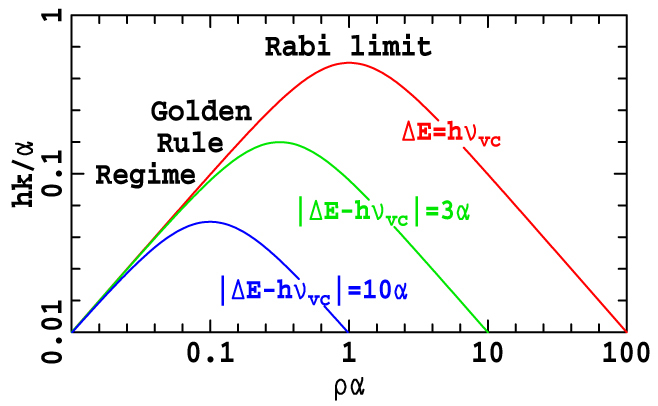
Decoherence “Volcano Diagram”^43^ showing the dimensionless representation of the dependence of the relaxation rate *k* on the Q_y_ density of states *ρ*, illustrating the Rabi Limit and Fermi-Golden-Rule regime (see text).

The Q_x_ → Q_y_ relaxation times for chlorophyllides are known to vary considerably with both chemical composition and environment^38^,^39^,^40^,^41^,^42^. These times can be determined from a complete spectral assignment. Our assignment includes properties critical to the kinetics such as the unperturbed band gap Δ*E* but *does not* include other essential effects such as the change in geometry between the Q_x_ and Q_y_ minima, Duschinsky rotation, and the nature of the many weakly-coupled vibronically active modes. However, these effects act essentially to modify the density of Q_y_ states at Δ*ν* = *ν_vc_*, allowing them to be incorporated into a single empirical parameter *ρ* taken as a universal constant independent of chlorophyllide and solvent. Further, the relaxation process is dominated by the interaction between the Q_x_ origin and the vibrational line of Q_y_ excited by one quantum of the dominant vibronic-coupling mode, leading to a two-level model for the process that is exactly solvable^43^ to yield a rate constant of 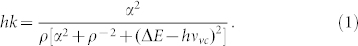
This equation is illustrated in Fig. 7 and takes the form of a "volcano diagram" in which the rate is slow if *ρ*^−1^ is either too small or too large (the "Golden-Rule limit") and is maximal at resonance (Δ*E* = *hν_vc_*) in the "Rabi limit" of *ρ* = 1/*α* where it is *hk* = *α*/2^43^. As the Q_x_ → Q_y_ relaxation is known to be extremely fast, only the Rabi limit is apt. In accordance with previously used assumptions, we take the density of states to be independent of chlorophyllide and solvent and hence choose the value *ρ* = 1/*α*; as on resonance d*k*/d*ρ* is zero, the rate constant is insensitive to changes in this density of states. The rate constant then becomes 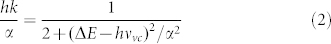
and so is highly sensitive to Δ*E*.

While the results from this simple two-level model qualitatively describe the observed phenomena, it is also straightforward to determine rate constants numerically by solving the quantum dynamics^43^ of our full vibronic-coupling model (see SI. Sect. S8). These numerically obtained results are given in Table 1.

Our vibronic-coupling assignment in the Rabi kinetics limit predicts a relaxation time of 99 fs for Chl-a in ether, in good agreement with the observed value^38^ of 100 ± 10 fs. The calculated value increases to 107, 122, 128 and 134 fs in pyridine, methanol/ethanol, 1-propanol, and 2-propanol, respectively, reproducing the observed^38^ magnitude of the increase in relaxation time as solvent polarity increases. The shortest relaxation time occurs when Δ*E* ≈ *hν_vc_* = 1500 cm^−1^, as found for say ChlZ(D1) of PS-II. This time increases to 161 fs for BChl-c, which has a much smaller band gap, and increases steeply for molecules like BChl-a with much larger band gaps. Larger band gaps also enhance the solvent dependence of the relaxation time: for BChl-a, the calculated lifetimes of 669 fs and 382 fs are determined for pyridine and ether solutions, respectively, reflecting the observed values spanning 100–400 fs in different solvents^39^,^40^, extending up to 4000 fs in reaction centres^41^. This high sensitivity comes simply from the band-gap dependence.

The observation that Chl-a in photosynthetic proteins can display maximal rates for Q_x_ decoherence may be an accident or could be a significant, previously unrecognized feature with practical consequences. More generally, the role, if any, of coherent vibrational motions in photosynthetic function remains an open question. Nevertheless, with respect to Q_x_ → Q_y_ relaxation, photosynthetic proteins incorporating various chlorophyllides still function well, despite marked changes of excitation decoherence. Coherent electronic motion caused by exciton transfer occurring on a faster timescale than vibrational decoherence is very important to the function of most photosynthetic organisms, however, and Q_x_ → Q_y_ relaxation must play some role in all Chl-a containing systems. Unfortunately, most advanced studies of quantum coherence effects in photosystems have focused on small and relatively homogeneous proteins like the FMO complex that contain BChl-a, systems for which decoherence effects are strongly muted.

In terms of the classical transition-state-theory Marcus-Hush model for electron-transfer and many other chemical reactions including most of photochemistry^44^,^45^, for BChl-a the large Δ*E* of 3200–4100 cm^−1^ makes the Q_x_ → Q_y_ relaxation highly exothermic with an associated large activation energy of Δ*E*^†^ = (−Δ*E* + *E_R_*)^2^/4*E_R_* (where *E_R_* is the relaxation energy, of order 500 cm^−1^), giving Boltzmann factors of 10^−8^–10^−32^ at room temperature. However, tunnelling *cancels* this effect for high-frequency modes with strong vibronic coupling of order *α*/*hν_vc_* = ½^46^, a value demonstrated to be appropriate for chlorophyllides by our model. Our approach thus allows the seemingly anomalous properties of internal conversion processes to be unified with standard chemical kinetics analyses.

## Methods

The methods used to fit the spectra are provided in Supporting Information. An efficient convolution method is used to generate the Huang-Rhys factors for all overtone and combination bands up to 3 quanta. As detailed in SI Section S4, the vibronic coupling spectral fits are obtained by solving the Q_x_ − Q_y_ interaction Hamiltonian 
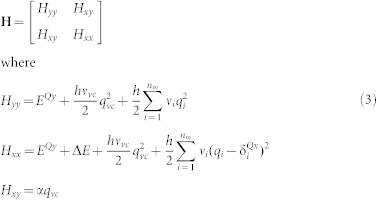
using a product harmonic-oscillator basis set truncated at 2912 levels per polarization to describe the vibronically active vibration (*ν_vc_*) and the (*n_m_* = 51) Franck-Condon allowed modes. This is done using our graphics-driven software FITMCD which uses time-dependent quantum mechanical methods to determine, in real time, the spectrum of Eqn. (3). So as to explicitly include inhomogeneous broadening effects, the final spectra are averaged over 11 different values of the Q_x_ − Q_y_ energy gap.

All TD-DFT calculations were performed at the CAM-B3LYP/6-31G* level^34^ as we implemented^47^ in a GAUSSIAN Development Version^48^; full details plus optimized Cartesian coordinates for 150 chlorophyllides, ligands, or chlorophyllide complexes with one or two solvent ligands attached are given in the SI; the solvents considered are: ether, acetone, methanol, 1-propanol, 2-propanol, water, and pyridine.

## Author Contributions

J.R.R., M.R., A.F. and E.K. designed the project, J.R.R. performed the spectral modelling, while Z.-L.C. and R.K. performed the DFT calculations.

## Supplementary Material

Supplementary Information Supplementary Information

Supplementary Information Supplementary Dataset 1

## Figures and Tables

**Table 1:** Fitted values of the unperturbed Q_x_ − Q_y_ spacing Δ*E* (1000 cm^−1^) and the corresponding fraction *f_x_* of absorption attributed to Q_x_ for chlorophyllides and related tetrapyrroles deduced from the vibronic-coupling model fit to the ABS and MCD spectra, see SI Sect. S4, as well as the inferred Q_x_ → Q_y_ relaxation times τ = 1/*k* (fs)

Sample	Δ*E*	*f_x_*	τ
BChl-a ether	4.14	0.21	669^c^^d^
Chlorin-e6 TME dioxane	3.58	0.07	477^d^
Methylpheophorbide-a dioxane	3.47	0.12	445^d^
Pheo-a EtOH/MeOH 1.7 K	3.38	0.2	419^d^
Pyromethylpheophorbide-a dioxane	3.36	0.1	414^d^
BChl-a pyridine	3.24	0.22	382^c^^d^
Ni(II)-Chl-a ether	2.75	0.07	231
Zn(II)-Chl-a ether	1.99	0.09	122
Chl-a ether	1.64	0.1	99^a^
BChl-d ether	1.38	0.1	95
ChlZ(D1) PS-II 1.7 K	1.35	0.1	95
BChl-c ether	1.15	0.16	99
Chl-a pyridine	0.97	0.17	107^b^
Chl-d MeOH/EtOH 1.7 K	0.81	0.23	119
Chl-a MeOH/EtOH 1.7 K	0.82	0.24	122^b^
Chl-a *n*-PrOH 1.8 K	0.75	0.21	128^b^
Chl-a *i*-PrOH 2.0 K 5CO	1.2	0.11	99
Chl-a *i*-PrOH 2.0 K 6CO	0.68	0.19	134^b^
BChl-d pyridine	0.71	0.17	128
BChl-c pyridine	0.44	0.17	161
tetrameso(3,5-di-*t*-butylphenyl) porphyrin	−2.42	0.33	-

^a^Obs.^38^ 100 ± 12 fs.

^b^Obs.^38^ times increase with solvent polarity, e.g., ethyl acetate 132 ± 10 fs, THF 138 ± 10 fs.

^c^Obs. 100–200 fs^39^ or 100–400 fs depending on environment^40^, 4800 fs in *Prosthecochloris aestuaril*^41^; processes with lifetimes as short as 30 fs have also been reported^42^.

^d^The calculations are likely to overestimate lifetimes at high Δ*E*, see SI. Sect. S8.
