# Supplementary material for: Assignment of the Q-Bands of the Chlorophylls: Coherence Loss via Qx − Qy Mixing
Source: Sci Rep. 2013 Sep 26;3:2761. doi: 10.1038/srep02761 (PMC3783888; doi:10.1038/srep02761)

# Assignment of the *Q*-Bands of the Chlorophylls: Coherence Loss via $Q_x$-$Q_y$ Mixing

Jeffrey R. Reimers,*[a] Zheng-Li Cai,[a] Rika Kobayashi,[b] Margus Rätsep,[c] Arvi Freiberg[c,d] and Elmars Krausz[e]

a. School of Chemistry, The University of Sydney, NSW 2006, Australia.
b. Australian National University Supercomputer Facility, Mills Rd, Canberra, ACT 0200, Australia.
c. Institute of Physics, University of Tartu, Riia 142, 51014 Tartu, Estonia.
d. Institute of Molecular and Cell Biology, University of Tartu, Riia 23, 51010 Tartu, Estonia.
e. Research School of Chemistry, The Australian National University, Canberra 2601, Australia.
* corresponding author email: Jeffrey.Reimers@Sydney.edu.au

# Supplementary Information

**Additional Supplementary Dataset:**

Cartesian_Coordinates.xls Optimized coordinates for 150 chlorophyllide clusters (see Table S6)

**Table of Contents:**

S1. Molecular structures

S2. Extraction of high-resolution properties of the $Q_y$ state of Chl-a in ether at 4.2 K from observed FE (and other) data.

S3. Comparison of the Huang-Rhys factors of Chl-a obtained from the FE data in ether and from hole-burning experiments in the natural photosystems WSCP and PSI-200.

S4. Fits to MCD and/or ABS spectra obtained by fitting vibronically coupled full $Q_x$ and $Q_y$ Franck-Condon-allowed band shapes.

S5. Conversion of observed LD to fraction $Q_x$ absorbance.

S6. CAM-B3LYP calculations of the Franck-Condon and Herzberg-Teller spectral envelopes of the $Q_y$ band.

S7. DFT calculations of spectral properties of dimeric and trimeric complexes of solvent molecules with chlorophyllides.

S8. $Q_x \rightarrow Q_y$ relaxation calculations.

S9. SI References

S10. Detailed fits to MCD and ABS spectra in Fig. 1 and Fig. S1.

## S1. Molecular structures

| Chlorophyllide | M | $R_3$ | $R_7$ | $R_8$ | $R_{12}$ | $R_{132}$ | $R_{17}$ | $R_{20}$ |
|---|---|---|---|---|---|---|---|---|
| Chlorophyll-a | Mg | $CH=CH_2$ | $CH_3$ | $CH_2CH_3$ | $CH_3$ | $COOCH_3$ | $C_2H_4COO$-phytyl[a] | H |
| Co(II)-Chlorophyll-a | Co | $CH=CH_2$ | $CH_3$ | $CH_2CH_3$ | $CH_3$ | $COOCH_3$ | $C_2H_4COO$-phytyl[a] | H |
| Ni(II)-Chlorophyll-a | Ni | $CH=CH_2$ | $CH_3$ | $CH_2CH_3$ | $CH_3$ | $COOCH_3$ | $C_2H_4COO$-phytyl[a] | H |
| Cu(II)-Chlorophyll-a | Cu | $CH=CH_2$ | $CH_3$ | $CH_2CH_3$ | $CH_3$ | $COOCH_3$ | $C_2H_4COO$-phytyl[a] | H |
| Zn(II)-Chlorophyll-a | Zn | $CH=CH_2$ | $CH_3$ | $CH_2CH_3$ | $CH_3$ | $COOCH_3$ | $C_2H_4COO$-phytyl[a] | H |
| Chlorophyll-b | Mg | $CH=CH_2$ | CHO | $CH_2CH_3$ | $CH_3$ | $COOCH_3$ | $C_2H_4COO$-phytyl[a] | H |
| Chlorophyll-c1 | Mg | $CH=CH_2$ | $CH_3$ | $CH_2CH_3$ | $CH_3$ | $COOCH_3$ | CH=CHCOOH | H |
| Chlorophyll-c2 | Mg | $CH=CH_2$ | $CH_3$ | $CH=CH_2$ | $CH_3$ | $COOCH_3$ | CH=CHCOOH | H |
| Chlorophyll-c3 | Mg | $CH=CH_2$ | $COOCH_3$ | $CH=CH_2$ | $CH_3$ | $COOCH_3$ | CH=CHCOOH | H |
| Chlorophyll-d | Mg | CHO | $CH_3$ | $CH_2CH_3$ | $CH_3$ | $COOCH_3$ | $C_2H_4COO$-phytyl[a] | H |
| Bacteriochlorophyll-c | Mg | $CHOHCH_3$ | $CH_3$ | $CH_2CH_3$[b] | $CH_2CH_3$[c] | H | $C_2H_4COO$-farnesyl[a] | $CH_3$ |
| Bacteriochlorophyll-d | Mg | $CHOHCH_3$ | $CH_3$ | $CH_2CH_3$[b] | $CH_2CH_3$[c] | H | $C_2H_4COO$-farnesyl[a] | H |
| Bacteriochlorophyll-e | Mg | $CHOHCH_3$ | CHO | $CH_2CH_3$[b] | $CH_2CH_3$[c] | H | $C_2H_4COO$-farnesyl[a] | $CH_3$ |
| Bacteriochlorophyll-f | Mg | $CHOHCH_3$ | CHO | $CH_2CH_3$[b] | $CH_2CH_3$[c] | H | $C_2H_4COO$-farnesyl[a] | H |

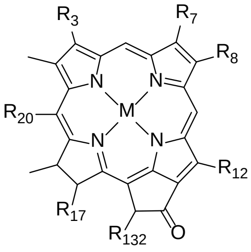


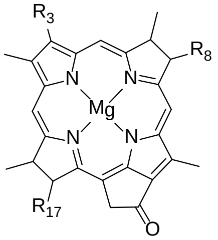


| Chlorophyllide[a] | $R_3$ | $R_7$ | $R_{132}$ |
|---|---|---|---|
| Pheophytin-a | $CH=CH_2$ | $CH_3$ | $COOCH_3$ |
| Methylpheophorbide-a | $CH=CH_2$ | $CH_3$ | $COOCH_3$ |
| Pheophytin-b | $CH=CH_2$ | CHO | $COOCH_3$ |
| Pheophytin-d | CHO | $CH_3$ | $COOCH_3$ |
| Pyromethylpheophorbide-a | $CH=CH_2$ | $CH_3$ | H |
| Mesopyromethylpheophorbide-a | $CH_2CH_3$ | $CH_3$ | H |

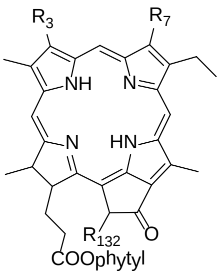


| Chlorophyllide | $R_3$ | $R_8$ | $R_{17}$ |
|---|---|---|---|
| Bacteriochlorophyll-a | $COCH_3$ | $-CH_2CH_3$ | $C_2H_4COO$-phytyl[a] |
| Bacteriochlorophyll-b | $COCH_3$ | $=CHCH_3$ | $C_2H_4COO$-phytyl[a] |
| Bacteriochlorophyll-g | $COCH_3$ | $=CH_2CH_3$ | $C_2H_4COO$-geranylgeraniol[a] |

| Chlorophyllide[d] | $R_{13}$ | $R_{15}$ |
|---|---|---|
| Chlorin-$e_6$ TME | $COOCH_3$ | $CH_2COOCH_3$ |
| Rhodochlorin DME | $COOCH_3$ | H |
| Isochlorin-$e_5$ DME | H | $CH_2COOCH_3$ |

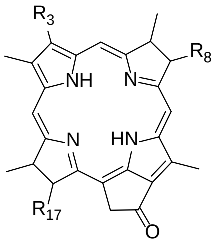


| Chlorophyllide | $R_3$ | $R_8$ | $R_{17}$ |
|---|---|---|---|
| Bacteriopheophytin-a | $COCH_3$ | $-CH_2CH_3$ | $C_2H_4COO$-phytyl[a] |
| Bacteriopheophytin-b | $COCH_3$ | $=CHCH_3$ | $C_2H_4COO$-phytyl[a] |
| Bacteriopheophytin-g | $COCH_3$ | $=CH_2CH_3$ | $C_2H_4COO$-geranylgeraniol[a] |

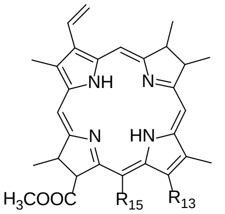


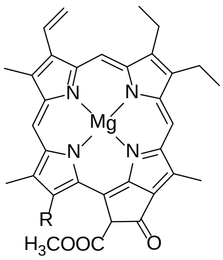


Protochlorophyll-a
R= $CH_2CH_2COO$phytyl

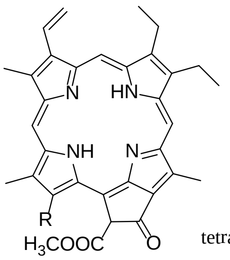


Protopheophytin-a
R= $CH_2CH_2COO$phytyl

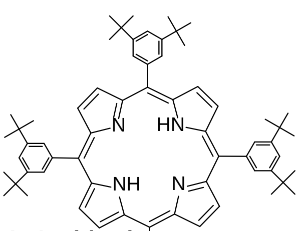


tetrameso(di-*t*-butylphenyl) porphyrin

a: calculations for methyl not phytyl (e.g., for Methylpheophorbide-a instead of Pheophytin-a)
b: can also be $C_3H_7$ or $C_4H_9$ (41)
c: can also be $CH_3$ (41)
d: TME= trimethyl ester, DME= dimethyl ester

**Chart S1.** Chlorophyllides and related macrocycles considered in this study.

**S2. Extraction of high-resolution properties of the $Q_y$ state of Chl-a in ether at 4.2 K from observed FE (and other) data.**

The observed high-resolution FE spectrum of Chl-a in ether at 4.2 K[1] contains data in the range of $\Delta\nu$ = 60-2300 $cm^{-1}$ from the $Q_y$ origin. As the origin region itself is not accessible by FE, the shapes of the zero-phonon line (ZPL) and its associated phonon side-band (PSB) are not fully discernible from this experiment, features required for the determination of the absolute magnitudes of the vibrational Huang-Rhys factors $S_i$. To determine absolute values, we inhomogeneously broaden the deduced high-resolution lines, normalizing the total $S$ value to reproduce the observed low-resolution contours observed in ABS for Chl-a in ether over the range 120 K to 295 K (see Sect. S4). However, the unobserved ZPL+PSB absorption shape is complex (see e.g. Fig. 2 insert) and requires much more detailed information to correctly reproduce. We use a standard analytical form for the ZPL and PSB shapes, expressing them as[2]

$$A'_{zpl}(\Delta\nu) = h_{zpl} \exp\frac{-\Delta\nu^2}{2\sigma_{zpl}^2} \qquad \text{(S1)}$$

and

$$A'_{psb}(\Delta\nu) = h_{psb} \exp\frac{-(\Delta\nu - \nu_{psb})^2}{2\sigma_{psb}^2} \qquad \text{(S2)}$$

if $\Delta\nu \le \nu_{psb}$ and the Lorentzian tail

$$A'_{psb}(\Delta\nu) = \frac{h_{psb}}{HWHM_{psb}^2 + (\Delta\nu - \nu_{psb})^2} \qquad \text{(S3)}$$

otherwise. The three Lorentzian parameters are fitted to the observed sharp rising background in the FE at low frequency, with no corrections applied for the inappropriateness of the Lorentzian functional form for $\Delta\nu \gg \nu_{psb}$, obtaining $h_{psb}$ = 3.1, $HWHM_{psb}$ = 96 $cm^{-1}$, and $\nu_{psb}$ = 35 $cm^{-1}$. The width of the ZPL line is fitted to the observed high-resolution vibrational line shapes as $\sigma_{zpl}$ = 3.422 $cm^{-1}$ (HWHM= 4.29 $cm^{-1}$) while the height of the ZPL line is fitted to the observed low-resolution band contours as $h_{zpl}$ = 80; all spectra are insensitive to the inner PSB Gaussian width and we arbitrarily set $\sigma_{zpl}$ = 18 $cm^{-1}$.

The observed FE contains not only high-resolution features attributed to individual *y*–polarized vibrational lines of $Q_y$ but also low-resolution data attributed to the inhomogeneously broadened $Q_x$ state and its associated vibronic coupling. Note that this inhomogeneity broadens the would-be sharp $Q_y$ vibrational bands as the vibronic coupling lowers their energy by of order $\alpha^2/(\Delta E - \nu_{vc})$, accessing the inhomogeneous broadening of the $Q_x$ state. We subtract the low-resolution *x*-polarized intensity, deduced by the MCD/ABS fit, from the observed FE before fitting the $Q_y$ Huang-Rhys factors.

An additional complication is that the sample measured by FE contained an unquantified mixture of 5CO and 6CO species (we have identified the 6CO species as being associated with water contamination[3]). By monitoring emission at 660 nm in the far high-frequency tail of the spectrum, Avarmaa and Rebane[1] aimed at measuring the spectrum of primarily the higher-energy 5CO species, having determined that its prominence increases rapidly as the monitored emission wavelength is decreased.[1,4] Given the inhomogeneous broadening at 4.2 K, the likely composition ratio, and that our fitting of the temperature dependence of the ABS and MCD spectra of Chl-a in ether indicate that the

6CO $Q_y$ origin lies 130 $cm^{-1}$ below the 5CO one (see Sect. S4), of order 5-10% of the FE signal is expected to arise from the 6CO species. As this estimate is too imprecise in nature and the expected contribution too low to fit to the experimental data, we chose to ignore any 6CO contribution to the spectrum.

The fit to the identified *y*-polarized non-ZPL contribution to the observed FE is then performed using $n_m$ = 236 individual vibrational modes (see Fig. 2). A maximum of $n_q$ =3 quanta of excitation in any individual mode is allowed (convergence of calculated spectra occurs at just 2 such quanta) an a convolution technique is used to calculate the full Franck-Condon allowed spectrum involving all possible multi-mode overtones of these individual-mode levels. This requires computer time of the order $n_m(n_q+1)$, taking for Chl-a of order 1 s to complete on a laptop computer. During this procedure, every line associated with single and multi-quanta excitations is given the same intrinsic lineshape, that of the ZPL+PSB. This procedure works by first representing the ZPL+PSB spectrum on a finite grid of points $\Delta\nu_j$ as

$$A'_{0,0}(\Delta\nu_j) = A'_{zpl}(\Delta\nu_j) + A'_{psb}(\Delta\nu_j)\,, \qquad \text{(S4)}$$

iteratively convolving in the Franck-Condon factors

$$\left\langle \chi_0^i \mid \chi_k^i \right\rangle^2 = \frac{S_i^k}{k!} e^{-S_i} \qquad \text{(S5)}$$

associated with each individual vibrational mode *i* at frequency $\nu_i$ with Huang-Rhys factor $S_i$, where $k \le n_q$ is the excitation level and $\left| \chi_k^i \right\rangle$ are the harmonic-oscillator vibrational wavefunctions, using

$$A'_{i,0}(\Delta\nu_j) = e^{-S_i} A'_{i-1,0}(\Delta\nu_j) \quad \forall \quad i, j \qquad \text{(S6)}$$

and

$$A'_{i,k}(\Delta\nu_j) = \frac{S_i^k}{k!} A'_{i,0}(\Delta\nu_j) \quad \forall \quad i, j, \qquad \text{(S7)}$$

with the final spectrum thus being given by $A'_{n_m,n_q}(\Delta\nu_j)$. Note that this analysis does not use the very commonly applied approximation[2]

$$S \approx \frac{I_{00}}{I_{00} + I_{10}} \qquad \text{(S8)}$$

which is only valid in the limit of $S \ll 1$, a limit that in general does not accurately apply for chlorophylls.

Full results for the fit to the FE data[1] for Chl-a in ether at 4.2 K are given in Table S1. This data is collapsed into 51 clearly resolved bands in Table S2, conserving the reorganization energy partitioned into each band; this process does not significantly affect the calculated low-resolution spectra. The resultant total Huang-Rhys factor is 0.278 whilst the total reorganization energy is 262 $cm^{-1}$.

**Table S1.** The full 236 lines (frequencies $\nu_i$, Huang Rhys factors $S_i$, and associated reorganization energies $\lambda_i = h\nu_i S_i$ extracted from fits to the FE spectrum of Chl-a in ether at 4.2 K[1] combined with low-resolution data in ether over the range 120 K – 295 K.

| $\nu_i$ / cm$^{-1}$ | 1000 $S_i$ | $\lambda_i$ / cm$^{-1}$ | $\nu_i$ / cm$^{-1}$ | 1000 $S_i$ | $\lambda_i$ / cm$^{-1}$ | $\nu_i$ / cm$^{-1}$ | 1000 $S_i$ | $\lambda_i$ / cm$^{-1}$ | $\nu_i$ / cm$^{-1}$ | 1000 $S_i$ | $\lambda_i$ / cm$^{-1}$ |
|---|---|---|---|---|---|---|---|---|---|---|---|
| 63.0 | 0.53 | 0.03 | 522.0 | 0.33 | 0.17 | 917.5 | 1.45 | 1.33 | 1318 | 1.56 | 2.05 |
| 70.0 | 0.00 | 0.00 | 529.0 | 0.25 | 0.13 | 925.0 | 1.52 | 1.41 | 1325 | 1.55 | 2.05 |
| 77.0 | 0.29 | 0.02 | 535.5 | 0.27 | 0.14 | 932.5 | 0.64 | 0.60 | 1331.5 | 1.75 | 2.33 |
| 85.0 | 0.43 | 0.04 | 542.5 | 0.11 | 0.06 | 940.0 | 0.59 | 0.55 | 1338.5 | 2.32 | 3.10 |
| 93.0 | 1.12 | 0.10 | 549.5 | 0.02 | 0.01 | 947.0 | 0.76 | 0.72 | 1345 | 3.31 | 4.45 |
| 100.0 | 1.32 | 0.13 | 556.5 | 0.11 | 0.06 | 954.5 | 1.41 | 1.34 | 1352 | 1.52 | 2.06 |
| 106.5 | 1.41 | 0.15 | 563.0 | 1.34 | 0.75 | 962.0 | 2.18 | 2.10 | 1358.5 | 1.42 | 1.94 |
| 113.5 | 1.80 | 0.20 | 570.0 | 2.82 | 1.61 | 969.5 | 3.47 | 3.36 | 1365.5 | 1.76 | 2.40 |
| 120.0 | 1.34 | 0.16 | 576.0 | 0.51 | 0.29 | 976.5 | 4.44 | 4.34 | 1373 | 2.36 | 3.25 |
| 126.5 | 1.26 | 0.16 | 581.0 | 0.56 | 0.33 | 984.0 | 6.60 | 6.49 | 1379.5 | 1.51 | 2.08 |
| 133.0 | 1.31 | 0.17 | 586.0 | 0.76 | 0.45 | 991.0 | 3.63 | 3.60 | 1385 | 1.28 | 1.77 |
| 140.0 | 1.77 | 0.25 | 591.0 | 1.40 | 0.83 | 998.0 | 2.14 | 2.13 | 1390 | 0.83 | 1.15 |
| 146.5 | 2.77 | 0.41 | 600.0 | 0.63 | 0.38 | 1005.0 | 2.15 | 2.16 | 1395 | 1.32 | 1.85 |
| 153.0 | 1.65 | 0.25 | 607.0 | 0.39 | 0.24 | 1011.5 | 1.73 | 1.75 | 1401.5 | 1.54 | 2.16 |
| 160.0 | 1.21 | 0.19 | 614.0 | 0.36 | 0.22 | 1017.5 | 1.66 | 1.69 | 1408.5 | 1.07 | 1.50 |
| 166.5 | 0.71 | 0.12 | 621.0 | 0.27 | 0.16 | 1024.0 | 1.49 | 1.53 | 1415 | 1.44 | 2.04 |
| 173.5 | 0.68 | 0.12 | 628.0 | 0.30 | 0.19 | 1030.0 | 1.48 | 1.53 | 1422 | 1.58 | 2.24 |
| 180.0 | 0.34 | 0.06 | 635.0 | 0.23 | 0.14 | 1037.5 | 1.61 | 1.67 | 1428.5 | 1.09 | 1.56 |
| 186.5 | 0.44 | 0.08 | 643.0 | 0.51 | 0.33 | 1045.0 | 1.13 | 1.18 | 1435.5 | 1.08 | 1.55 |
| 193.5 | 0.62 | 0.12 | 649.0 | 0.50 | 0.32 | 1051.0 | 0.65 | 0.69 | 1442 | 1.00 | 1.44 |
| 199.5 | 0.50 | 0.10 | 656.0 | 0.28 | 0.19 | 1057.0 | 0.85 | 0.90 | 1449 | 1.04 | 1.50 |
| 206.0 | 0.31 | 0.06 | 663.0 | 0.26 | 0.17 | 1063.0 | 0.93 | 0.99 | 1455.5 | 0.85 | 1.24 |
| 249.5 | 0.62 | 0.16 | 670.0 | 0.14 | 0.09 | 1069.0 | 0.86 | 0.92 | 1462.5 | 0.67 | 0.98 |
| 256.5 | 1.50 | 0.38 | 676.0 | 0.26 | 0.17 | 1075.5 | 1.80 | 1.94 | 1469.5 | 0.58 | 0.86 |
| 263.5 | 2.82 | 0.74 | 682.0 | 0.38 | 0.26 | 1083.0 | 1.06 | 1.15 | 1476 | 0.57 | 0.84 |
| 269.5 | 1.77 | 0.48 | 688.0 | 0.56 | 0.39 | 1089.5 | 0.89 | 0.96 | 1483 | 0.61 | 0.90 |
| 276.0 | 0.98 | 0.27 | 695.5 | 0.94 | 0.66 | 1096.0 | 0.59 | 0.64 | 1489.5 | 0.58 | 0.87 |
| 284.0 | 1.42 | 0.40 | 703.0 | 0.77 | 0.54 | 1103.0 | 0.72 | 0.79 | 1496.5 | 1.41 | 2.11 |
| 290.0 | 1.04 | 0.30 | 710.5 | 1.00 | 0.71 | 1111.0 | 0.89 | 0.99 | 1503 | 2.42 | 3.64 |
| 297.0 | 0.73 | 0.22 | 717.5 | 0.70 | 0.51 | 1116.5 | 1.28 | 1.43 | 1510 | 3.04 | 4.59 |
| 304.0 | 0.61 | 0.19 | 725.0 | 0.89 | 0.65 | 1122.5 | 0.79 | 0.89 | 1516.5 | 2.41 | 3.65 |
| 311.0 | 0.43 | 0.13 | 732.5 | 2.69 | 1.97 | 1129.0 | 0.63 | 0.71 | 1523.5 | 1.95 | 2.97 |
| 318.0 | 0.45 | 0.14 | 739.5 | 3.81 | 2.82 | 1135.0 | 2.04 | 2.31 | 1530 | 1.90 | 2.90 |
| 325.0 | 1.49 | 0.49 | 745.5 | 4.48 | 3.34 | 1141.5 | 0.64 | 0.73 | 1536.5 | 2.20 | 3.38 |
| 332.5 | 0.39 | 0.13 | 752.5 | 2.30 | 1.73 | 1148.0 | 0.73 | 0.83 | 1544 | 1.61 | 2.49 |
| 340.5 | 1.58 | 0.54 | 760.0 | 1.99 | 1.51 | 1155.0 | 0.78 | 0.90 | 1551.5 | 1.27 | 1.97 |
| 348.0 | 4.23 | 1.47 | 768.0 | 1.80 | 1.38 | 1161.5 | 1.24 | 1.44 | 1558.5 | 1.00 | 1.56 |
| 355.5 | 1.53 | 0.55 | 773.0 | 1.15 | 0.89 | 1168.0 | 1.74 | 2.04 | 1565.5 | 0.72 | 1.13 |
| 363.0 | 1.08 | 0.39 | 778.0 | 1.29 | 1.00 | 1175.0 | 1.36 | 1.60 | 1572.5 | 0.69 | 1.09 |
| 370.0 | 1.80 | 0.67 | 783.0 | 1.02 | 0.80 | 1181.5 | 1.13 | 1.33 | 1579.5 | 0.62 | 0.97 |
| 376.5 | 1.30 | 0.49 | 788.0 | 1.43 | 1.13 | 1188.5 | 1.09 | 1.29 | 1586.5 | 0.63 | 0.99 |

| | | | | | | | | | | | |
|---|---|---|---|---|---|---|---|---|---|---|---|
| 383.5 | 1.26 | 0.48 | 793.0 | 0.87 | 0.69 | 1196.0 | 1.54 | 1.85 | 1594 | 0.55 | 0.88 |
| 390.0 | 1.57 | 0.61 | 798.5 | 1.23 | 0.99 | 1203.5 | 1.22 | 1.47 | 1601 | 0.44 | 0.70 |
| 397.0 | 1.14 | 0.45 | 805.5 | 1.12 | 0.90 | 1209.0 | 0.53 | 0.64 | 1608 | 0.41 | 0.66 |
| 403.5 | 0.72 | 0.29 | 812.0 | 1.11 | 0.90 | 1214.0 | 0.55 | 0.67 | 1615 | 0.33 | 0.53 |
| 410.5 | 0.93 | 0.38 | 819.0 | 0.86 | 0.71 | 1219.0 | 1.55 | 1.89 | 1622 | 0.29 | 0.48 |
| 417.5 | 0.45 | 0.19 | 825.5 | 0.78 | 0.64 | 1225.5 | 1.74 | 2.13 | 1629 | 0.32 | 0.52 |
| 424.0 | 0.39 | 0.17 | 832.5 | 0.77 | 0.64 | 1229.0 | 1.51 | 1.86 | 1636.5 | 0.21 | 0.35 |
| 431.0 | 0.27 | 0.12 | 839.5 | 0.59 | 0.49 | 1235.5 | 3.87 | 4.79 | 1643.5 | 0.16 | 0.27 |
| 437.5 | 0.09 | 0.04 | 847.0 | 0.80 | 0.68 | 1242.0 | 3.80 | 4.72 | 1650.5 | 0.20 | 0.33 |
| 444.5 | 0.09 | 0.04 | 853.0 | 0.49 | 0.42 | 1250.0 | 6.40 | 8.01 | 1657.5 | 0.24 | 0.39 |
| 465.0 | 0.34 | 0.16 | 859.5 | 0.51 | 0.44 | 1256.5 | 2.11 | 2.65 | 1664.5 | 0.30 | 0.50 |
| 472.0 | 0.33 | 0.16 | 866.0 | 0.26 | 0.22 | 1262.5 | 2.25 | 2.84 | 1671.5 | 0.24 | 0.40 |
| 479.5 | 0.38 | 0.18 | 872.0 | 0.34 | 0.30 | 1269.0 | 2.01 | 2.56 | 1679 | 0.19 | 0.31 |
| 486.5 | 0.14 | 0.07 | 878.0 | 0.70 | 0.61 | 1275.0 | 2.73 | 3.48 | 1686 | 0.13 | 0.22 |
| 493.5 | 0.06 | 0.03 | 884.0 | 0.80 | 0.70 | 1282.0 | 2.30 | 2.94 | 1693 | 0.10 | 0.18 |
| 500.5 | 0.32 | 0.16 | 890.0 | 0.81 | 0.72 | 1289.0 | 1.94 | 2.50 | 1700 | 0.13 | 0.22 |
| 508.0 | 0.24 | 0.12 | 896.5 | 0.99 | 0.89 | 1296.5 | 1.71 | 2.22 | **total** | **278** | **262** |
| 515.0 | 0.05 | 0.02 | 903.5 | 1.07 | 0.97 | 1303.5 | 1.36 | 1.77 | | | |
| | | | 910.0 | 1.59 | 1.45 | 1310.5 | 1.49 | 1.96 | | | |

**Table S2.** The fitted 236 lines from Table S1 are collapsed into 51 resolved bands: peak frequencies $\nu_i$ and band frequency ranges ($cm^{-1}$), effective Huang Rhys factors $S_i$, and associated conserved band reorganization energies $\lambda_i = h\nu_i S_i$ ($cm^{-1}$).

| **$\nu_i$ peak** | **$S_i$ *1000** | **$\lambda_i$** | ν min | ν max | **$\nu_i$ peak** | **$S_i$ *1000** | **$\lambda_i$** | ν min | ν max |
|---|---|---|---|---|---|---|---|---|---|
| 74.0 | 1.3 | 0.1 | 63.0 | 85.0 | 812.0 | 5.1 | 4.1 | 798.5 | 825.5 |
| 110.0 | 8.3 | 0.9 | 93.0 | 126.5 | 846.5 | 3.2 | 2.7 | 832.5 | 859.5 |
| 150.0 | 9.3 | 1.4 | 133.0 | 166.5 | 878.0 | 2.9 | 2.6 | 866.0 | 890.0 |
| 190.0 | 2.9 | 0.5 | 173.5 | 206.0 | 903.5 | 3.7 | 3.3 | 896.5 | 910.0 |
| 263.0 | 7.7 | 2.0 | 249.5 | 276.0 | 925.0 | 3.6 | 3.3 | 917.5 | 932.5 |
| 290.5 | 3.2 | 0.9 | 284.0 | 297.0 | 965.5 | 23.3 | 22.5 | 940.0 | 991.0 |
| 314.5 | 3.0 | 0.9 | 304.0 | 325.0 | 1008.0 | 7.7 | 7.7 | 998.0 | 1017.5 |
| 344.0 | 7.8 | 2.7 | 332.5 | 355.5 | 1034.0 | 5.7 | 5.9 | 1024.0 | 1045.0 |
| 370.0 | 4.2 | 1.5 | 363.0 | 376.5 | 1069.5 | 7.1 | 7.6 | 1051.0 | 1089.5 |
| 390.0 | 4.0 | 1.5 | 383.5 | 397.0 | 1106.5 | 3.5 | 3.9 | 1096.0 | 1116.5 |
| 410.5 | 2.1 | 0.9 | 403.5 | 417.5 | 1132.0 | 4.1 | 4.6 | 1122.5 | 1141.5 |
| 434.5 | 0.8 | 0.4 | 424.0 | 444.5 | 1165.0 | 7.0 | 8.1 | 1148.0 | 1181.5 |
| 465.0 | 0.3 | 0.2 | 465.0 | 465.0 | 1196.0 | 3.9 | 4.6 | 1188.5 | 1203.5 |
| 476.0 | 0.7 | 0.3 | 472.0 | 479.5 | 1268.0 | 11.4 | 14.5 | 1209.0 | 1544.0 |
| 493.5 | 0.5 | 0.3 | 486.5 | 500.5 | 1253.0 | 14.5 | 18.2 | 1242.0 | 1262.5 |
| 515.0 | 0.6 | 0.3 | 508.0 | 522.0 | 1286.0 | 12.0 | 15.5 | 1269.0 | 1303.5 |
| 535.5 | 0.6 | 0.3 | 529.0 | 542.5 | 1331.5 | 13.5 | 18.0 | 1310.5 | 1352.0 |
| 563.0 | 4.8 | 2.7 | 549.5 | 576.0 | 1369.0 | 7.1 | 9.7 | 1358.5 | 1379.5 |
| 586.0 | 2.7 | 1.6 | 581.0 | 591.0 | 1393.0 | 5.0 | 6.9 | 1385.0 | 1401.5 |
| 610.5 | 1.6 | 1.0 | 600.0 | 621.0 | 1415.0 | 4.1 | 5.8 | 1408.5 | 1422.0 |
| 639.0 | 1.5 | 1.0 | 628.0 | 649.0 | 1445.5 | 5.7 | 8.3 | 1428.5 | 1462.5 |
| 669.5 | 1.3 | 0.9 | 656.0 | 682.0 | 1493.0 | 11.7 | 17.5 | 1469.5 | 1516.5 |
| 703.0 | 4.0 | 2.8 | 688.0 | 717.5 | 1530.0 | 6.1 | 9.3 | 1523.5 | 1536.5 |
| 739.0 | 14.2 | 10.5 | 725.0 | 752.5 | 1586.5 | 6.9 | 11.0 | 1551.5 | 1622.0 |
| 770.0 | 6.2 | 4.8 | 760.0 | 778.0 | 1664.5 | 2.2 | 3.7 | 1629.0 | 1700.0 |
| 788.0 | 3.3 | 2.6 | 783.0 | 793.0 | **total** | **278** | **262** | | |

**S3. Comparison of the Huang-Rhys factors of Chl-a obtained from the FE data in ether and from hole-burning experiments in the natural photosystems WSCP and PSI-200.**

The hole burning spectra obtained for Chl-a in various photosystems differ significantly from each other,[5] indicating significant dependence of both the total magnitude of the Huang-Rhys factor and its vibrational distribution upon local environment. Large differences between chlorophyll in situ and in solvents has also been noted.[2] For example, in Table S3 high-resolution data for Chl-a in ether (this work, from FE) is compared to that obtained from hole burning experiments on the WSCP[5] and PSI-200[6] photosystems, with the total Huang-Rhys factors being $S$= 0.278 in ether, 0.79 in WSCP, and 0.57 in PS1-200; more crudely estimated values in other solvents (Table S4) are $S$= 0.28 in pyridine, 0.42 in 1-propanol (0.39 previously[2] using a similar scaling method), and 0.38 in 2-propanol. These results suggest that this effect in not controlled primarily by magnesium coordination, and indeed a large difference is also found between the Huang-Rhys factor of 0.45 deduced for Pheo-a in EtOH/MeOH and 0.20 deduced for its methylated derivative methylpheophorbide-a in dioxane (Table S4).

**Table S3.** Comparison of individual Huang-Rhys factors $S_i$ obtained for Chl-a in ether (this work, based on FE data at 4.2 K combined with low-resolution band contours for 120 – 295 K) to those extracted from hole-burning experiments on the WSCP[5] and PSI-200[6] photosystems.

| **WSCP** | | | **PS-I-200** | | | **FE data** | | |
|---|---|---|---|---|---|---|---|---|
| $\nu_i$ / cm$^{-1}$ | 1000 $S_i$ | $\lambda_i$ / cm$^{-1}$ | $\nu_i$ / cm$^{-1}$ | 1000 $S_i$ | $\lambda_i$ / cm$^{-1}$ | $\nu_i$ / cm$^{-1}$ | 1000 $S_i$ | $\lambda_i$ / cm$^{-1}$ |
| | | | | | | 74 | 1.3 | 0.1 |
| | | | | | | 110 | 8.3 | 0.9 |
| 162 | 2 | 0.3 | | | | 150 | 9.3 | 1.4 |
| 178 | 4 | 0.7 | | | | | | |
| 192 | 6 | 1.2 | | | | 190 | 2.9 | 0.6 |
| 207 | 10 | 2.1 | | | | | | |
| 232 | 11 | 2.6 | | | | | | |
| 266 | 6 | 1.6 | 262 | 11.9 | 3.1 | 263 | 7.7 | 2.0 |
| 280 | 4 | 1.1 | 283 | 3.9 | 1.1 | 291 | 3.2 | 0.9 |
| 304 | 6 | 1.8 | | | | 315 | 3.0 | 0.9 |
| 327 | 23 | 7.5 | | | | | | |
| 352 | 15 | 5.3 | | | | 344 | 7.8 | 2.7 |
| 360 | 17 | 6.1 | | | | | | |
| 371 | 3 | 1.1 | | | | 370 | 4.2 | 1.6 |
| 385 | 14 | 5.4 | 390 | 15.1 | 5.9 | 390 | 4.0 | 1.6 |
| 406 | 7 | 2.8 | | | | 411 | 2.1 | 0.9 |
| 429 | 10 | 4.3 | 425 | 7.1 | 3.0 | 435 | 0.8 | 0.3 |
| 469 | 11 | 5.2 | 469 | 19.0 | 8.9 | 465 | 0.3 | 0.1 |
| 482 | 4 | 1.9 | | | 0.0 | 476 | 0.7 | 0.3 |
| 494 | 3 | 1.5 | 501 | 7.0 | 3.5 | 494 | 0.5 | 0.2 |
| 520 | 23 | 12.0 | 521 | 17.1 | 8.9 | 515 | 0.6 | 0.3 |
| | | | 541 | 9.1 | 4.9 | 534 | 0.6 | 0.3 |
| 572 | 12 | 6.9 | 574 | 25.1 | 14.4 | 563 | 4.8 | 2.7 |
| 580 | 10 | 5.8 | 588 | 4.9 | 2.9 | 586 | 2.7 | 1.6 |
| 604 | 14 | 8.5 | 607 | 12.0 | 7.3 | 611 | 1.6 | 1.0 |
| | | | 638 | 9.0 | 5.7 | 640 | 1.5 | 1.0 |
| | | | | | | 670 | 1.3 | 0.9 |
| 685 | 18 | 12.3 | 692 | 15.0 | 10.4 | 703 | 4.0 | 2.8 |
| 709 | 33 | 23.4 | 714 | 9.9 | 7.1 | | | |
| 734 | 13 | 9.5 | | | | 739 | 14.2 | 10.5 |
| 740 | 27 | 20.0 | | | | | | |
| 747 | 16 | 12.0 | 746 | 44.1 | 32.9 | | | |
| 755 | 17 | 12.8 | | | | | | |
| 766 | 9 | 6.9 | | | | | | |
| 776 | 13 | 10.1 | 771 | 7.0 | 5.4 | 770 | 6.2 | 4.8 |
| 783 | 14 | 11.0 | | | | | | |
| 797 | 15 | 12.0 | 791 | 14.1 | 11.2 | 788 | 3.3 | 2.6 |
| 803 | 13 | 10.4 | 805 | 12.0 | 9.7 | | | |
| 816 | 13 | 10.6 | 819 | 5.0 | 4.1 | 812 | 5.1 | 4.1 |
| 833 | 14 | 11.7 | 855 | 9.0 | 7.7 | 847 | 3.2 | 2.7 |

| | | | | | | | | |
|---|---|---|---|---|---|---|---|---|
| 877 | 23 | 20.2 | 864 | 7.0 | 6.0 | 878 | 2.9 | 2.5 |
| | | | 874 | 7.0 | 6.1 | | | 0.0 |
| | | | 896 | 13.0 | 11.6 | 904 | 3.7 | 3.3 |
| 920 | 47 | 43.2 | 932 | 25.1 | 23.4 | 925 | 3.6 | 3.3 |
| 984 | 39 | 38.4 | 994 | 28.1 | 27.9 | 966 | 23.3 | 22.5 |
| 1000 | 11 | 11.0 | | | | | | |
| 1005 | 6 | 6.0 | 1009 | 5.0 | 5.0 | 1008 | 7.7 | 7.8 |
| 1032 | 32 | 33.0 | | | | 1034 | 5.7 | 5.9 |
| 1054 | 15 | 15.8 | | | | | | |
| 1072 | 24 | 25.7 | 1075 | 12.0 | 12.9 | 1070 | 7.1 | 7.6 |
| 1106 | 16 | 17.7 | 1114 | 9.0 | 10.0 | 1107 | 3.5 | 3.9 |
| 1122 | 19 | 21.3 | | | | | | |
| 1136 | 39 | 44.3 | | | | 1132 | 4.1 | 4.6 |
| 1176 | 14 | 16.5 | 1178 | 18.1 | 21.3 | 1165 | 7.0 | 8.2 |
| 1184 | 5 | 5.9 | | | | | | |
| 1196 | 3 | 3.6 | 1203 | 12.0 | 14.5 | 1196 | 3.9 | 4.7 |
| 1228 | 3 | 3.7 | | | | | | |
| 1243 | 13 | 16.2 | 1259 | 40.9 | 51.5 | 1253 | 14.5 | 18.2 |
| 1265 | 2 | 2.5 | | | | 1268 | 11.4 | 14.5 |
| 1280 | 4 | 5.1 | | | | | | 0.0 |
| 1286 | 2 | 2.6 | 1285 | 11.0 | 14.1 | 1286 | 12.0 | 15.4 |
| 1296 | 2 | 2.6 | | | | | | |
| 1314 | 3 | 3.9 | | | | | | |
| 1320 | 2 | 2.6 | 1340 | 11.0 | 14.7 | 1332 | 13.5 | 18.0 |
| 1350 | 10 | 13.5 | 1364 | 32.0 | 43.7 | 1369 | 7.1 | 9.7 |
| 1378 | 6 | 8.3 | 1390 | 18.1 | 25.1 | 1393 | 5.0 | 7.0 |
| 1422 | 3 | 4.3 | 1411 | 5.0 | 7.1 | 1415 | 4.1 | 5.8 |
| 1433 | 3 | 4.3 | 1433 | 9.0 | 12.9 | 1446 | 5.7 | 8.2 |
| | | | 1455 | 5.9 | 8.6 | | | |
| 1495 | 5 | 7.5 | 1465 | 6.1 | 8.9 | 1493 | 11.7 | 17.5 |
| 1517 | 9 | 13.7 | 1504 | 9.9 | 15.0 | | | |
| 1543 | 2 | 3.1 | 1524 | 32.0 | 48.8 | 1530 | 6.1 | 9.3 |
| | | | | | | 1587 | 6.9 | 11.0 |
| | | | | | | 1665 | 2.2 | 3.7 |
| **Total** | **792**[a] | **645** | | **574** | **547** | | **278** | **262** |

a: in good agreement with other estimates;[7] from ΔFLN, $S$=0.80.[8]

**S4. Fits to MCD and/or ABS spectra obtained by fitting vibronically coupled full $Q_x$ and $Q_y$ Franck-Condon-allowed band shapes.**

The potential-energy surfaces of the $Q_y$ and $Q_x$ states, respectively, are represented in terms of $n_m$ = 51 dimensionless normal coordinates of the $Q_y$ state $q_i$, coupled by a single vibronically active mode $q_{vc}$ as

$$\mathbf{H}=\begin{bmatrix} E^{Qy}+\frac{h\nu_{vc}}{2}q_{vc}^2+\frac{h}{2}\sum_{i=1}^{n_m}\nu_i q_i^2 & \alpha q_{vc} \\ \alpha q_{vc} & E^{Qy}+\Delta E+\frac{h\nu_{vc}}{2}q_{vc}^2+\frac{h}{2}\sum_{i=1}^{n_m}\nu_i(q_i-\delta_i^{Qx})^2 \end{bmatrix} \quad \text{(S9)}$$

where $\delta_i^{Qx}$ are the coordinate displacements between the minima of the $Q_y$ and $Q_x$ states; many alternate forms of expressing this equation are available[9-16] but they are all equivalent.[17] This electronic matrix is then expressed in terms of a vibronic basis set formed as a product of harmonic-oscillator wavefunctions containing $k_i$ quanta of excitation $\left|\chi_{k_i}\right\rangle$ for each mode, truncating the maximum number of vibrational quanta in the Franck-Condon displaced modes at $\sum_{i=1}^{n_m} k_i$ = 2 and the number of quanta in the vibronically active mode at 4. Calculations performed with larger basis sets indicate that convergence is obtained at these truncations. This generates 1456 basis states $\left|\psi_{\mathbf{k}k_\alpha}^{Qy}\right\rangle$ for the $Q_y$ state plus an equivalent set $\left|\psi_{\mathbf{k}k_\alpha}^{Qx}\right\rangle$ for $Q_x$, where $\mathbf{k}$ is a vector of the quanta $k_i$ in each Franck-Condon mode and $k_\alpha$ is the number of quanta in the vibronically active mode. The matrix elements are obtained as the product of Franck-Condon overlap integrals and vibronic-coupling terms as

$$\begin{aligned} \left\langle\psi_{\mathbf{k}k_\alpha}^{Qy}\right|\mathbf{H}\left|\psi_{\mathbf{k}'k'_\alpha}^{Qy}\right\rangle &= \delta_{\mathbf{k},\mathbf{k}'}\delta_{k_\alpha,k'_\alpha}\left[E^{Qy}+(k_\alpha+1/2)h\nu_{vc}+\sum_{i=1}^{n_m}(k_i+1/2)h\nu_i\right], \\ \left\langle\psi_{\mathbf{k}k_\alpha}^{Qx}\right|\mathbf{H}\left|\psi_{\mathbf{k}'k'_\alpha}^{Qx}\right\rangle &= \delta_{\mathbf{k},\mathbf{k}'}\delta_{k_\alpha,k'_\alpha}\left[E^{Qy}+\Delta E+(k_\alpha+1/2)h\nu_{vc}+\sum_{i=1}^{n_m}(k_i+1/2)h\nu_i\right], \quad \text{(S10)} \\ \left\langle\psi_{\mathbf{k}k_\alpha}^{Qy}\right|\mathbf{H}\left|\psi_{\mathbf{k}'k'_\alpha}^{Qx}\right\rangle &= \delta_{|k_\alpha-k'_\alpha|,1}\sqrt{\frac{\max(k_\alpha,k'_\alpha)}{2}}\left[\prod_{i=1}^{n_m}\left\langle\chi_{k_i}(0)\,|\,\chi_{k'_i}(\delta_i^{Qx})\right\rangle\right]. \end{aligned}$$

Simple diagonalization of this Hamiltonian matrix would yield the eigenstates of the vibronically coupled band system and hence the ABS spectrum given the unperturbed $Q_y$ and $Q_x$ transition moment matrices:

$$\begin{aligned} \left\langle\psi_{00}^{GS}\right|\mathbf{M}\left|\psi_{\mathbf{k}k_\alpha}^{Qy}\right\rangle &= \delta_{k_\alpha,0}\left[\prod_{i=1}^{n_m}\left\langle\chi_0(\delta_i^{GS})\,|\,\chi_{k_i}(0)\right\rangle\right]\hat{\mathbf{y}} \\ \left\langle\psi_{00}^{GS}\right|\mathbf{M}\left|\psi_{\mathbf{k}k_\alpha}^{Qx}\right\rangle &= \delta_{k_\alpha,0}\left[\prod_{i=1}^{n_m}\left\langle\chi_0(\delta_i^{GS})\,|\,\chi_{k_i}(\delta_i^{Qx})\right\rangle\right]\hat{\mathbf{x}}, \end{aligned} \quad \text{(S11)}$$

where $\hat{\mathbf{x}}$ and $\hat{\mathbf{y}}$ are orthogonal unit vectors in the molecular coordinate frame and $\delta_i^{GS}$ are the differences in geometry between the $Q_y$ state and the ground state (GS) , with the total Huang-Rhys factor of the $Q_y$ state given by

$$S = \frac{1}{2}\sum_{i=1}^{n_m}(\delta_i^{GS})^2 . \tag{S12}$$

However, such an approach is very computationally intensive and instead a numerically equivalent time-dependent procedure is utilized. Initial wavefunctions representing Franck-Condon absorption from the zero-point level of the ground state to the $Q_y$ and $Q_x$ states are then written from Eqn. (S11) as

$$\begin{aligned} \left|\Psi_y(0)\right\rangle &= \left[\prod_{i=1}^{n_m}\left\langle \chi_0(\delta_i^{GS}) \mid \chi_{k_i}(0)\right\rangle \left|\chi_{k_i}(0)\right\rangle\right]\left|\chi_{0_\alpha}(0)\right\rangle, \\ \left|\Psi_x(0)\right\rangle &= \left[\prod_{i=1}^{n_m}\left\langle \chi_0(\delta_i^{GS}) \mid \chi_{k_i}(\delta_i^{Qx})\right\rangle \left|\chi_{k_i}(0)\right\rangle\right]\left|\chi_{0_\alpha}(0)\right\rangle. \end{aligned} \tag{S13}$$

These non-stationary states are then propagated forward in time $t$ using the appropriate time-dependent Schrödinger equations

$$\begin{aligned} i\hbar\frac{\mathrm{d}\left|\Psi_y(t)\right\rangle}{\mathrm{d}t} &= \mathbf{H}\left|\Psi_y(t)\right\rangle \quad \text{and} \\ i\hbar\frac{\mathrm{d}\left|\Psi_x(t)\right\rangle}{\mathrm{d}t} &= \mathbf{H}\left|\Psi_x(t)\right\rangle, \end{aligned} \tag{S14}$$

operations that require only repeated matrix-vector products to evaluate. From these, the autocorrelation functions

$$\begin{aligned} c_y(t) &= \left\langle \Psi_y(0) \mid \Psi_y(t)\right\rangle \quad \text{and} \\ c_x(t) &= \left\langle \Psi_x(0) \mid \Psi_x(t)\right\rangle \end{aligned} \tag{S15}$$

are deduced and converted to ABS spectra by Fourier-Laplace transform

$$\begin{aligned} \sigma_y(\nu) &= \mathrm{Re}\left[\int_0^\infty c_y(t)w(t)e^{2\pi i\nu t}\mathrm{d}t\right] \quad \text{and} \\ \sigma_x(\nu) &= \mathrm{Re}\left[\int_0^\infty c_x(t)w(t)e^{2\pi i\nu t}\mathrm{d}t\right], \end{aligned} \tag{S16}$$

where $w(t)$ is the Fourier Transform of the spectral lineshape function comprising the inhomogeneous broadening of the $Q_y$ state, the zero-phonon lineshape, and the phonon-side band, multiplied by the Fourier transform of the Gaussian inhomogeneous-broadening function. Only this function is used to include thermal effects on the spectrum, but this method can be readily extended to include explicitly thermally populated vibrational levels if necessary.[18]

This procedure automatically includes the inhomogeneous broadening of the $Q_y$ state but that for $Q_x$ is much larger owing to the stronger interaction of $Q_x$ with its environment. This additional contribution is included by integrating over the associated inhomogeneity $\Delta E'$ in the $Q_x$-$Q_y$ gap $\Delta E$

$$\sigma_y(\nu) = \frac{1}{2\sqrt{2\pi}(\sigma_{Qx} - \sigma_{Qy})} \int_{-\infty}^{\infty} \sigma_y(\nu;\Delta E') e^{\frac{-(\Delta E')^2}{2(\sigma_{Qx}-\sigma_{Qy})^2}} \mathrm{d}\Delta E'$$

$$\sigma_x(\nu) = \frac{1}{2\sqrt{2\pi}(\sigma_{Qx} - \sigma_{Qy})} \int_{-\infty}^{\infty} \sigma_x(\nu;\Delta E') e^{\frac{-(\Delta E')^2}{2(\sigma_{Qx}-\sigma_{Qy})^2}} \mathrm{d}\Delta E' \quad \text{(S17)}$$

implemented by just adding $\Delta E'$ to $\Delta E$ in Eqn. S9, etc.; here $\sigma_{Qy}$ and $\sigma_{Qx}$ are the inhomogeneous broadenings of $Q_y$ and $Q_x$, respectively. In the program FITMCD, these integrations are performed using an 11-point Simpson's Rule quadrature, but the averaging is actually performed for the autocorrelation functions rather than for the final spectra. A total of 22 2912×2912 matrices **H** are thus constructed per molecular component, but the spectra are determined quickly enough to facilitate interactive mouse-driven adjustment of the model parameters by our program FITMCD.

The ABS and MCD spectra in terms of these normalized bandshape functions are then expressed as

$$A'(\nu) = A(\nu)/\nu = D_x\sigma_x(\nu) + D_y\sigma_y(\nu) \quad \text{(S18)}$$

and

$$\Delta\Delta A'(\nu) = \frac{\Delta A(\nu,H) - \Delta A(\nu,0)}{H\nu} = B_x\sigma_x(\nu) + B_y\sigma_y(\nu) \quad \text{(S19)}$$

respectively, where *H* is the applied magnetic-field strength, *D* are absorption dipole strengths, and *B* and MCD susceptibilities (only this MCD "B" term[19] is usually included in the analysis of chlorophyllides).

For spectra arising from just a single molecular component, 12 adjustable parameters naturally arise in our vibronic coupling model: the two vibronic-coupling parameters $\nu_{vc}$ and α, the two unperturbed origin frequencies, the two ABS dipole strengths $D_x$ and $D_y$, the associated MCD sensitivities $B_x$ and $B_y$, the associated inhomogeneous-broadening FWHM, and two parameters associated with the Franck-Condon Huang-Rhys factors *S* of $Q_x$ and $Q_y$. Other parameters in the model such as those specifying the zero-phonon-line shape and the phonon-side-band are not adjusted. The relative Huang-Rhys factors $S_i = (\delta_i^{GS})^2/2$ for each of the 51 vibrational lines of the $Q_y$ state relative to the ground state are frozen and only its total magnitude *S* that is adjusted for each chlorophyllide. This is a somewhat crude approximation but one which exposes the intrinsic similarities of the properties of the different chlorophyllides as well as the important feature that the geometry change between the ground state and $Q_y$ is quite sensitive to both the nature of the chlorophyllide and the solvation environment. Replacing this approximation by a more realistic one requires the measurement and accurate interpretation of high-resolution spectra for each individual chlorophyllide in each solvation environment.

For most chlorophyllides, it is assumed that the Franck-Condon factors for the $Q_x$ state are the same as those for $Q_y$. This is a much poorer approximation than the previous one as the two states are expected to have significantly different geometries. As Eqns. S11 and S13 indicate, (unsigned) Huang-Rhys factors alone are insufficient to describe the spectral properties of two vibronically coupled states, the actual (signed) displacements $\delta$ being required instead. Even if high-resolution

spectra for $Q_x$ were available, the determination of this sign information would be a seriously difficult task. As it stands, only information concerning the $Q_x$ band contour is available, but this highly inhomogeneously broadened band contour must also be extracted from underneath the much more intense $Q_y$ band for many chlorophyllides. For those molecules for which $Q_x$ is well resolved, the Huang-Rhys factors for $Q_x$ appear similar to those for $Q_y$ and so it appears reasonable to take them as being equal. For Chl-a and its close analogues like Chl-d, BChl-c and BChl-d, the observed MCD signal shows much more intensity in the region of $Q_x$ + 500 $cm^{-1}$ than does $Q_y$, however, so we introduce an arbitrary rescaling of the displacements for these modes, leading to the prescription

$$\begin{aligned} \delta_i^{Qx} &= -2\delta_i^{GS} \quad \text{if} \quad \nu_i < 700\,\text{cm}^{-1} \\ &= 0 \qquad\quad \text{otherwise.} \end{aligned} \tag{S20}$$

The same molecular vibration frequencies are used for all states of all molecules. The effect of the above rescaling is to increase the spectral reorganization energy associated with absorption from the ground state from 262 $cm^{-1}$ for $Q_y$ to 380 $cm^{-1}$ for $Q_x$.

In practical terms, fitting the vibronic coupling model to the observed ABS and MCD spectra involves the determination of 7 non-trivial parameters: the unperturbed $Q_x$-$Q_y$ gap $\Delta E$, the fraction of absorption $f_x = D_x/(D_x + D_y)$ with $x$ polarization, the ratios $B_x/D_x$ and $B_y/D_y$, the two inhomogeneous-broadening FWHM, and the total Huang-Rhys factor $S$. Appropriate values for the FWHM and for $S$ are usually clear from the experimental data whilst the deduced values for the other 4 parameters strongly reflect the appropriateness of the vibronic-coupling model itself. The deduced values for the most critical parameters $\Delta E$ and $f_x$ are discussed in detail in the main text.

In addition, the rules for MCD spectroscopy place internal constraints on the deduced values of $B_x/D_x$ and $B_y/D_y$, focusing on, in particular, their dependence on chlorophyllide and solvation environment. If the unperturbed gap $\Delta E$ is sufficiently large so that perturbation theory may be applied to express the MCD *B*-term response, then a critical feature is that $B_y/D_y$ scales with the reciprocal energy gap so that $\Delta E \times B_y/D_y$ should be a universal constant. Exploitation of this feature has historically played a significant role in the justification of the "modern" assignment for the spectrum of Chl-a.[20] However, this result is only expected if the $Q_x$ transition is formally forbidden (i.e., $f_x$ = 0), and the related constraints affecting $B_x/D_x$ are rarely considered. In a more general approach we introduce the quantities

$$\eta_x = \frac{-B_x \Delta E}{D_x + D_y} \quad \text{and} \quad \eta_y = \frac{B_y \Delta E}{D_x + D_y} \tag{S21}$$

to represent the fitted MCD susceptibilities.

Our description of the Q-band spectra explicitly assumes that no other spectroscopic transitions are involved. This is a good approximation in that the next highest energy transitions, the Soret band, is well removed from the Q band for all molecules considered. Under such circumstances, standard MCD theory[19,21-23] indicates that $B_x = -B_y$ (or equivalently $\eta_x = \eta_y$). However, interactions with the distant Soret bands invalidate this relationship and hence we assume that these are independent quantities. We have also developed an independent *analytical* method for analysing the MCD data which verifies this key aspect of our fitting procedure.[24]

We analyse many spectra taken from the previous 50 years' literature plus in addition some new measured spectra at low temperature for Chl-a. Magnetic Circular Dichroism and Absorption

measurements were made simultaneously using the system as previously described,[25] consisting of a Spex 0.75 monchromator and an Oxford Instruments SM4 superconducting magnet cryostat and a highly stabilized quartz halogen light source. Samples were rapidly quenched in a helium gas environment (30 sec) to 5 K in a 2 mm path-length cylindrical cell having fused quartz windows. The cell windows were bonded to a thin-wall stainless steel or titanium body, similar to designs previously described.[26] This construction minimized strain in the sample and avoided fracturing the quartz windows. Spectra were taken in the absence and presence of a 5 Tesla applied magnetic field, so as to account for CD baselines and artefacts. The concentration of Chl-a (sourced from *Anacystis nidulans* algae, Aldrich) in the various solvents was adjusted to provide a peak $Q_y$ absorption of ~0.2 in a 2 mm cell. This largely eliminated aggregation effects, which would otherwise be obvious from the MCD and CD spectra.[27-29] Reagent grade solvents/solvent mixtures were used except diethyl-ether, which was rigorously dried and distilled under vacuum. Dry ether samples were rapidly transferred to the sample cell and immediately quenched to 5 K in the sample cryostat. The wet ether sample was prepared by adding one drop of water to 0.5 ml of the dry diethyl-ether/Chl-a mixture and stirring for 5 minutes.

Table S4 gives the 7 non-trivial fitted parameters for the 12 systems discussed in Fig. 1 of the main text, as well as those for 17 other systems shown in Fig. S1. For Chl-a in ether at low temperature, the observed spectra include contributions from both 5CO and 6CO components. For these samples, two sets of 7 parameters are required, along with a 15$^{th}$ parameter indicating the relative fraction of the absorption attributed to the 6CO species. A 16$^{th}$ parameter, the difference between the 6CO and 5CO $Q_y$ origins also arises, but for all samples we constrain the 6CO origin to be 130 cm$^{-1}$ lower in energy than that for 5CO. Quantification of this energy difference is difficult as the perceived value is dependent on assumptions made concerning the inhomogeneous broadening, but 130 cm$^{-1}$ is consistent with a wide range of data including detailed high-resolution FE measurements.[1,4,30] A value of 140 cm$^{-1}$ results in a less consistent representation of the other parameters while a value much less than this would seem inconsistent with the qualitative picture depicted by the FE. For samples containing mixed species, all parameters except the composition are frozen, allowing the observed temperature and concentration dependence to be represented using just a single sensitive parameter (other parameters such as the overall *Q*-band band location and intensity do vary slightly as well). Mixed species are also found in 2-propanol at low temperature, and for this system 6CO $Q_y$ is fitted to be 95 cm$^{-1}$ lower in energy that 5CO. Other samples of 2-propanol at low temperature[2] have revealed just a single component (5CO), and it is clear that composition is strongly dependent on formation conditions in these glasses.

From Table S4 we see that the energy-gap scaled MCD susceptibilities can show marked variations amongst the chlorophyllides, with $\eta_y$ ranging from 0.25 T$^{-1}$ cm$^{-1}$ for Ni(II)-Chl-a in ether to 2.63 T$^{-1}$ cm$^{-1}$ for methylpheophorbide-a in dioxane; hence the expectation[20,31,32] based on the assumption that the magnetic and electronic transition moments that combine to provide the MCD *B*-term response[19,20,31,32] are invariant to solvation and to variations in the macrocycle is not valid. However, the variation found between similar species is much less, with for example $\eta_y$ = 0.75–0.9 T$^{-1}$ cm$^{-1}$ for Chl-a and BChl-a, ~1.1 T$^{-1}$ cm$^{-1}$ for Pheo-a and BChl-c, and ~1.2 T$^{-1}$ cm$^{-1}$ for BChl-d. Also, the naïve expectation that $\eta_y$ should be a universal constant also leads[19,21-23] to the approximation $\eta_x = \eta_y$ but from Table S4 we see that for Chl-a and related species $\eta_x$ is 10-20 % larger than $\eta_y$, for some chlorophyllides it can be up to five times larger. This analysis has been independently verified.[24]

The results shown in Fig. 1 of the main text indicate that the vibronic-coupling model depicts the major qualitative effects controlling the observed ABS and MCD spectra of the chlorophyllides.

There are, however, distinct quantitative shortcomings. Form Figs. 1a and 1d for BChl-a, it is clear that the Huang-Rhys factors for this molecule are proportionately larger at $Q_y(0,0)+500\ cm^{-1}$, but the vibronically stolen intensity at $+1000\ cm^{-1}$ is well reproduced, indicating that the magnitude of the vibronic coupling is very similar for Chl-a and BChl-a. For Pheo-a in Fig 1b the strength of the vibronic coupling appears to have increased slightly, while for Chlorin-e6 in Fig S1c the magnitude of the vibronic coupling constant appears to double. Similarly, Zn(II)-Chl-a (Fig. 1e) appears to have similar vibronic coupling to Chl-a whilst it is clearly much larger for Ni(II)-Chl-a (Fig. S1g).

More significantly, the one-mode vibronic-coupling model quantitatively fails to predict the correct magnitude of *x*-polarization in the $+1500\ cm^{-1}$ region for 5CO Chl-a, BChl-c, and BChl-d, correspondingly also failing to reproduce the correct width of the band at this location for 6CO species (Fig. 1j-l). These deficiencies should be corrected if a full multi-mode treatment of the vibronic coupling is implemented, but at the moment insufficient high-resolution data is available to construct such a model authoritatively.

The spectra of Chl-b and protochlorophyll-a appear to depict dramatically reduced vibronic coupling compared to Chl-a. Further experimental work re-examining the MCD spectra, as well as computational work investigating the vibronic coupling, is warranted.

**Table S4.** Critical parameters fitted to the ABS and MCD spectra of chlorophyllides using the vibronic-coupling model: $\Delta E$ is the unperturbed $Q_x$-$Q_y$ energy gap, $f_x$ is the fraction of absorption with *x*-polarization, the MCD sensitivities are expressed through energy-gap scaled parameters $\eta$ (Eqn. S21), and the identified fraction of 6-coordinate species in mixtures; for ether/eater mixtures, the 6CO $Q_y$ band origin is always at 130 $cm^{-1}$ below the corresponding 5CO origin, while this is 95 $cm^{-1}$ below for 2-propanol.[a]

| **Sample** | **CO** | ***S*** | $\Delta E$ **1000 cm$^{-1}$** | $f_x$ | **HWHM$_y$ 1000 cm$^{-1}$** | **HWHM$_x$ 1000 cm$^{-1}$** | $\eta_y$ **T$^{-1}$ cm$^{-1}$** | $\eta_x$ **T$^{-1}$ cm$^{-1}$** | **fract. 6CO** |
|---|---|---|---|---|---|---|---|---|---|
| BChl-a ether | 5 | 0.200 | 4.14 | 0.21 | 0.24 | 0.49 | 0.81 | 1.33 | |
| Pheo-a EtOH/MeOH 1.7 K | | 0.450 | 3.38 | 0.20 | 0.15 | 0.25 | 1.09 | 1.78 | |
| Pyromethylpheophorbide-a dioxane | | 0.200 | 3.36 | 0.10 | 0.20 | 0.29 | 2.11 | 9.85 | |
| BChl-a pyridine | 6 | 0.240 | 3.24 | 0.22 | 0.25 | 0.45 | 0.86 | 1.37 | |
| Zn(II)-Chl-a ether[b] | 5 | 0.278* | 1.99 | 0.09 | 0.19 | 0.43 | 0.37 | 0.63 | |
| Chl-a ether | 5 | 0.278* | 1.64 | 0.10 | 0.18 | 0.36 | 0.79 | 1.16 | |
| BChl-d ether | 5 | 0.330 | 1.38 | 0.10 | 0.14 | 0.38 | 1.26 | 1.84 | |
| BChl-c ether | 5 | 0.310 | 1.15 | 0.15 | 0.14 | 0.32 | 1.10 | 1.42 | |
| ChlZ(D1) PS-II 1.7 K | 5 | 0.278* | 1.35 | 0.10 | 0.11 | 0.31 | 0.82 | 1.25 | |
| Chl-a pyridine | 6 | 0.278* | 0.97 | 0.17 | 0.20 | 0.31 | 0.94 | 1.16 | |
| BChl-d pyridine | 6 | 0.278* | 0.71 | 0.17 | 0.17 | 0.33 | 1.21 | 1.49 | |
| BChl-c pyridine | 6 | 0.278* | 0.44 | 0.17 | 0.17 | 0.33 | 1.11 | 1.28 | |
| Rhodochlorin DME dioxane | | 0.200 | 3.69 | 0.07 | 0.19 | 0.28 | 2.10 | 4.64 | |
| Isochlorin-e5 DME dioxane | | 0.200 | 3.59 | 0.04 | 0.17 | 0.23 | 2.25 | 3.58 | |
| Chlorin-e6 TME dioxane | | 0.120 | 3.58 | 0.07 | 0.20 | 0.33 | 1.48 | 6.52 | |
| Methylpheophorbide-a dioxane | | 0.200 | 3.47 | 0.13 | 0.19 | 0.24 | 2.63 | 8.71 | |
| Meso-pyromethylpheophorbide-a dioxane | | 0.200 | 3.29 | 0.14 | 0.15 | 0.24 | 3.29 | 5.83 | |
| Pheo-d MeOH/EtOH 1.7 K | | 0.450 | 3.48 | 0.15 | 0.15 | 0.21 | 1.04 | 1.67 | |
| Ni(II)-Chl-a ether[b] | 5 | 0.350 | 2.75 | 0.07 | 0.25 | 0.60 | 0.25 | 0.56 | |
| Cu(II)-Chl-a ether[b] | 5 | 0.350 | 2.64 | 0.07 | 0.28 | 0.63 | 0.20 | 0.36 | |
| Co(II)-Chl-a ether[b] | 5 | 0.350 | 2.23 | 0.09 | 0.50 | 0.83 | 0.27 | 0.27 | |
| Chl-a ether[b] | 5 | 0.278* | 1.65 | 0.09 | 0.18 | 0.41 | 0.67 | 0.99 | |
| Chl-b MBBA+EBBA+DAB | 5 | 0.320 | 1.31 | 0.18 | 0.28 | 0.28 | 1.07 | 2.11 | |
| Chl-d MeOH/EtOH 1.7 K | 5 | 0.278* | 0.81 | 0.23 | 0.18 | 0.38 | 0.75 | 0.89 | |
| Chl-a MeOH/EtOH 1.7 K | 6 | 0.420 | 0.82 | 0.24 | 0.12 | 0.19 | 0.89 | 1.06 | |
| Chl-a *n*-PrOH 1.8 K | 6 | 0.42 | 0.752 | 0.21 | 0.133 | 0.227 | 0.864 | 0.967 | |
| Chl-a *n*-PrOH 160 K | 6 | 0.42 | 0.798 | 0.11 | 0.175 | 0.315 | 0.867 | 0.978 | |
| Chl-a *i*-PrOH 1.8 K | 5 | 0.38 | 1.20 | 0.11 | 0.18 | 0.323 | 0.818 | 0.993 | 0.62 |
| | 6 | 0.38 | 0.68 | 0.19 | 0.15 | 0.259 | 0.691 | 0.833 | |
| Protochlorophyll-a ether | 5 | 0.320 | 0.68 | 0.22 | 0.16 | 0.20 | 0.57 | 1.23 | |

a: the experimental data and completed fits are shown in Fig. 1 of the main text or in Fig. S1, with detailed decompositions provided in the associated file "Detailed_analyses.pdf"; experimental data sources are listed with the figure captions.
b: MCD intensities from Nonomura et al.[33] are rescaled to align results for Chl-a with other measurements.
*: $Q_x$ modes < 700 $cm^{-1}$ are rescaled, see Eqn. S20.

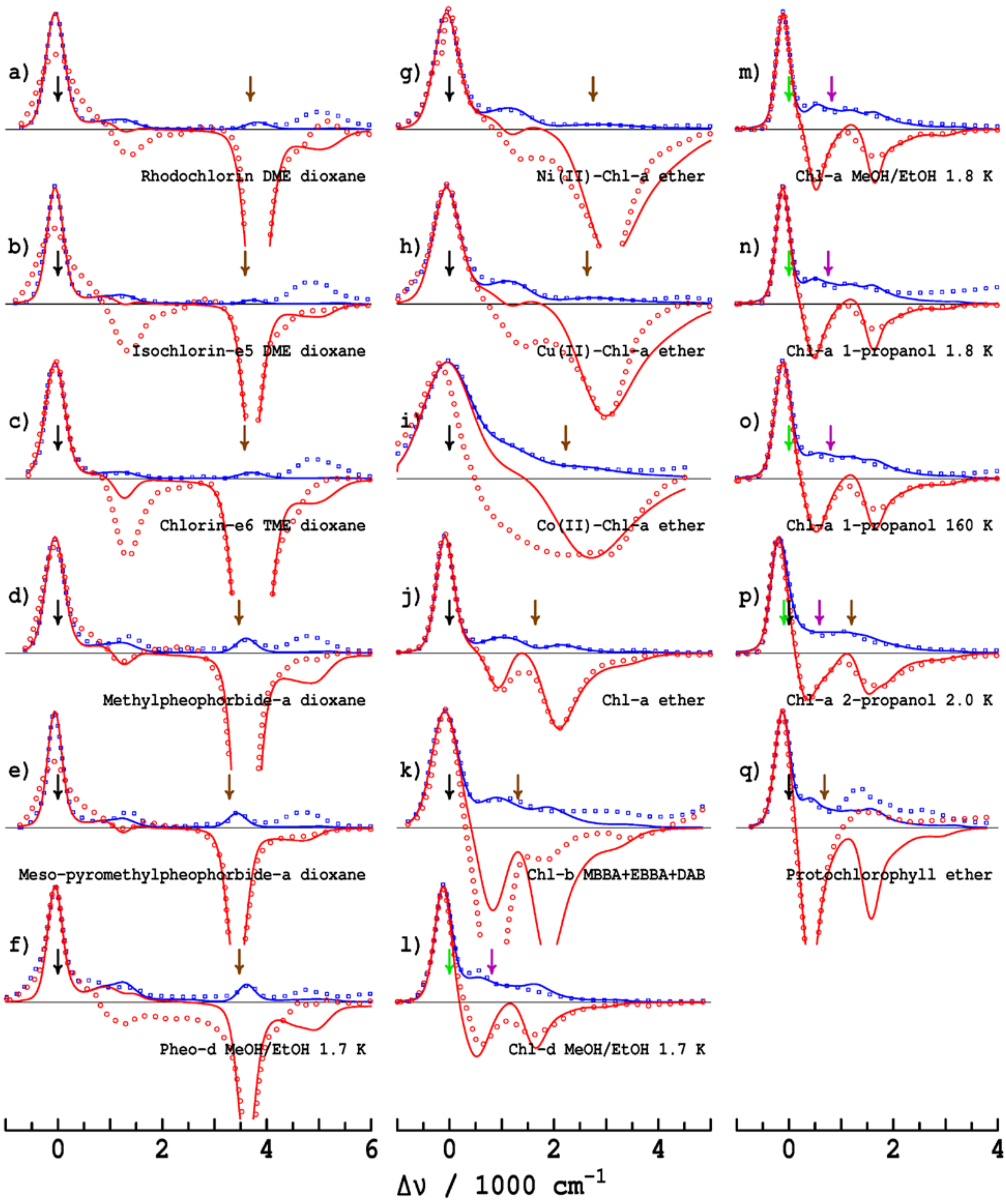


**Fig. S1.** Observed ABS ( $A'$, blue dots) and MCD ( $\Delta A'$, red dots) spectra and their fits (lines) obtained using a vibronic coupling model with $\nu_{vc}$ =1500 cm$^{-1}$ and α=750 cm$^{-1}$. Solvents are as indicated, measurements were made at room temperature unless otherwise noted. Unperturbed origins are indicated by arrows: black- free-base and 5CO $Q_y$, brown- free-base and 5CO $Q_x$, green- 6CO $Q_y$, purple- 6CO $Q_x$. Key fitted parameters are listed in Table S4. All spectra are broadened using a Gaussian function of HWHM= 47 cm$^{-1}$ to reduce noise, obtained from: a-e Briat;[34] f- Razeghifard;[35] g-j- Nonomura;[33] k- Frackowiak;[36] l-q this work; q- Weiss.[37]

## S5. Conversion of observed LD to fraction $Q_x$ absorbance.

Fragata et al.[38,39] observed the LD polarized absorption of Chl-a in an aligned lamellar phase of glycerylmonooctanoate/$H_2O$. They found that the $Q_y$ transition was polarized at an angle of 70° to that of $Q_x$. As a result, intensity from the $Q_y$ band is recorded amongst the $Q_x$ absorption. We correct for this effect using

$$A_x^{pure} = A_x - \cos(70°) A_y \qquad \text{(S22)}$$

which eliminates the *x*-polarized intensity that tracks the $Q_y$ band shape.

Avarmaa and Suisala[4] measured the polarized FE spectrum of Chl-a in ether at 4.2 K by exciting an isotropically aligned sample with linearly polarized light, detecting the resulting polarized emission. The concentrations used are too low to permit exciton energy transfer and so only unimolecular relaxation processes are allowed. Under these conditions, the observed anisotropy $r$ is related to the angle β between the absorbing dipole (which may contain some mixture of $Q_x$ and $Q_y$ absorption) and the emitting dipole (assumed to be pure $Q_y$):[40]

$$r = \frac{1}{5}\left(3\cos^2\beta - 1\right) \qquad \text{(S23)}$$

but under the experimental conditions the observed polarization (Ref.[4] Fig. 1) is related to the anisotropy by[40]

$$p = \frac{3r}{2+r} \qquad \text{(S24)}$$

so that the polarization in the *x*-*y* plane is given by

$$p_{xy} = \frac{A_y - A_x}{A_y + A_x} = \frac{7p-1}{3-p} \ . \qquad \text{(S25)}$$

This quantity, which is unbiased with respect to *x* and *y*, is shown in Fig. 4b.

In Fig. 4b, the polarizations extracted from the LD, polarized FE and MCD experiments are qualitatively similar to each other and in particular show the same types of variations in going from 5CO to 6CO species. Monitoring at 665 nm, the polarized FE is most likely to arise from Chl-a molecules with over 80% 5CO, whereas monitoring at 675 nm is likely to sample a more equal proportion of 5CO and 6CO species. Unfortunately, details of the composition cannot be accurately determined as the water content[3] of the sample is unknown and the emission band contour has not been quantitatively apportioned into contributions from 5CO and 6CO species.

## S6. CAM-B3LYP calculations of the Franck-Condon and Herzberg-Teller spectral envelopes of the $Q_y$ band.

The general properties of spectral calculation methods for chlorophyllides and other molecules has recently been extensively reviewed.[41] Here, the Franck-Condon absorption envelope for the $Q_y$ band of Chl-a (Fig. 3) was determined from the DFT-optimized structure of the ground state and the TD-DFT optimized structure and normal coordinates of $Q_y$. These calculations were performed by GAUSSIAN-09[42] using the 6-31G* basis set[43] and the CAM-B3LYP[44] density-functional. The fully optimized Cartesian displacements between the two adiabatic minima were projected onto the normal modes using curvilinear internal coordinates using the DUSHIN program[45] and the dimensionless normal-mode displacements $\delta_i^{GS}$ thus obtained. A similar approach has been shown to quantitatively describe the significant high-resolution asymmetry seen between the ABS and EMI spectra of BChl-a.[46] The ABS spectrum is synthesized using the convolution techniques described in Sect. S2 but the results obtained are identical to those that would have been obtained by evaluating Eqn. S11 directly.

The corresponding Herzberg-Teller ($x$-polarized) component of the $Q_y$ spectrum shown in Fig. 3 of the main text was evaluated by first calculating the vibronic-coupling constants $\alpha_i$ by numerical differentiation of the perpendicular component of the ground-state to $Q_y$ transition-moment vector $\mathbf{M}_y$ with respect to displacements in the normal modes $q_i$ of $Q_y$:[14,16]

$$\alpha_i = \frac{\Delta E}{\left|\mathbf{M}_x(0)\right| q_i}\left|\mathbf{M}_y(q_i) - \mathbf{M}_y(0) - \left(\mathbf{M}_y(q_i) - \mathbf{M}_y(0)\right)\cdot\frac{\mathbf{M}_y(0)}{\left|\mathbf{M}_y(0)\right|}\right| \qquad \text{(S26)}$$

Always the transition moment was taken to be the geometrical mean of the length-formalism and velocity-formalism transition moments reported by GAUSSIAN.[47] Note that this approach utilizes also the $Q_x$ transition-moment vector predicted by CAM-B3LYP as well as the associated energy gap. Once the vibronic-coupling constants are determined, the spectrum is simulated by application of the standard zero-phonon-line and phonon-side-band shapes and expanded to include Franck-Condon progressions based on each Herzberg-Teller origin. The intensity of each Herzberg-Teller origin is obtained from perturbation theory (rather than full solution of **H**) as

$$S_i' = \left(\frac{\alpha_i}{\Delta E - h\nu_i}\right)^2 . \qquad \text{(S27)}$$

The calculated Franck-Condon displacements, Huang-Rhys factors, and reorganization energies, as well as the corresponding Herzberg-teller vibronic-coupling constants, Huang-Rhys factors, and reorganization energies are given in Table S5. For the Franck-Condon term, the calculated reorganization energy is 505 $cm^{-1}$, nearly double the observed value (Table S2) of 262 $cm^{-1}$, while the Herzberg-Teller term is 615 $cm^{-1}$ (reducing to 374 $cm^{-1}$ after damping in a condensed media of modes < 30 $cm^{-1}$ is included) compared to $750^2/1500/2=188$ $cm^{-1}$ used in the vibronic-coupling model fits. While closer agreement for experiment has been found for BChl-a,[46] the values are all actually very small making accurate calculation difficult, with reasonable computational methods often being in error by an order of magnitude.[46]

**Table S5.** CAM-B3LYP/6-31G* calculated Franck-Condon displacements δ, Huang-Rhys factors *S*, and reorganization energies λ ($cm^{-1}$), as well as the corresponding Herzberg-Teller vibronic-coupling constants α ($cm^{-1}$), Huang-Rhys factors, and reorganization energies ($cm^{-1}$) for Chl-a in the gas phase.

| | Franck-Condon | | | Herzberg-Teller | | | | Franck-Condon | | | Herzberg-Teller | | |
|---|---|---|---|---|---|---|---|---|---|---|---|---|---|
| ν | δ | *S* | λ | α | *S* | λ | ν | δ | *S* | λ | α | *S* | λ |
| 1775 | -0.028 | 0.0004 | 0.7 | 0 | 0.0000 | 0.0 | 897 | -0.114 | 0.0065 | 5.8 | 78 | 0.0038 | 3.4 |
| 1762 | 0.026 | 0.0003 | 0.6 | 5 | 0.0000 | 0.0 | 895 | 0.097 | 0.0047 | 4.2 | 66 | 0.0027 | 2.4 |

| | | | | | | | | | | | | | |
|---|---|---|---|---|---|---|---|---|---|---|---|---|---|
| 1758 | -0.023 | 0.0003 | 0.4 | 36 | 0.0002 | 0.4 | 890 | -0.032 | 0.0005 | 0.5 | 48 | 0.0014 | 1.3 |
| 1659 | -0.032 | 0.0005 | 0.8 | 26 | 0.0001 | 0.2 | 884 | 0.072 | 0.0026 | 2.3 | 20 | 0.0003 | 0.2 |
| 1617 | 0.092 | 0.0042 | 6.8 | 27 | 0.0001 | 0.2 | 856 | 0.016 | 0.0001 | 0.1 | 25 | 0.0004 | 0.4 |
| 1590 | 0.120 | 0.0072 | 11.4 | 55 | 0.0006 | 1.0 | 846 | 0.008 | 0.0000 | 0.0 | 10 | 0.0001 | 0.1 |
| 1586 | 0.002 | 0.0000 | 0.0 | 97 | 0.0019 | 3.0 | 839 | -0.063 | 0.0020 | 1.7 | 26 | 0.0005 | 0.4 |
| 1568 | -0.097 | 0.0047 | 7.4 | 55 | 0.0006 | 1.0 | 831 | 0.020 | 0.0002 | 0.2 | 45 | 0.0015 | 1.2 |
| 1562 | -0.036 | 0.0006 | 1.0 | 30 | 0.0002 | 0.3 | 806 | 0.049 | 0.0012 | 1.0 | 20 | 0.0003 | 0.2 |
| 1538 | 0.179 | 0.0160 | 24.6 | 106 | 0.0024 | 3.6 | 789 | -0.030 | 0.0004 | 0.3 | 62 | 0.0031 | 2.4 |
| 1522 | -0.003 | 0.0000 | 0.0 | 81 | 0.0014 | 2.1 | 786 | 0.018 | 0.0002 | 0.1 | 8 | 0.0001 | 0.0 |
| 1501 | -0.054 | 0.0014 | 2.2 | 251 | 0.0140 | 21.0 | 783 | 0.009 | 0.0000 | 0.0 | 5 | 0.0000 | 0.0 |
| 1489 | 0.052 | 0.0014 | 2.0 | 5 | 0.0000 | 0.0 | 779 | 0.035 | 0.0006 | 0.5 | 18 | 0.0003 | 0.2 |
| 1486 | -0.139 | 0.0096 | 14.3 | 22 | 0.0001 | 0.2 | 769 | 0.035 | 0.0006 | 0.5 | 21 | 0.0004 | 0.3 |
| 1481 | 0.118 | 0.0070 | 10.3 | 49 | 0.0005 | 0.8 | 764 | 0.056 | 0.0016 | 1.2 | 11 | 0.0001 | 0.1 |
| 1479 | -0.002 | 0.0000 | 0.0 | 27 | 0.0002 | 0.2 | 757 | 0.065 | 0.0021 | 1.6 | 25 | 0.0005 | 0.4 |
| 1479 | 0.024 | 0.0003 | 0.4 | 34 | 0.0003 | 0.4 | 755 | -0.028 | 0.0004 | 0.3 | 4 | 0.0000 | 0.0 |
| 1478 | 0.022 | 0.0002 | 0.3 | 9 | 0.0000 | 0.0 | 749 | -0.030 | 0.0004 | 0.3 | 7 | 0.0000 | 0.0 |
| 1472 | -0.012 | 0.0001 | 0.1 | 32 | 0.0002 | 0.3 | 739 | 0.119 | 0.0071 | 5.3 | 12 | 0.0001 | 0.1 |
| 1471 | 0.051 | 0.0013 | 1.9 | 46 | 0.0005 | 0.7 | 737 | 0.168 | 0.0140 | 10.3 | 12 | 0.0001 | 0.1 |
| 1470 | 0.047 | 0.0011 | 1.6 | 8 | 0.0000 | 0.0 | 720 | -0.090 | 0.0040 | 2.9 | 28 | 0.0008 | 0.6 |
| 1470 | -0.006 | 0.0000 | 0.0 | 91 | 0.0019 | 2.8 | 716 | -0.050 | 0.0013 | 0.9 | 8 | 0.0001 | 0.0 |
| 1468 | -0.006 | 0.0000 | 0.0 | 12 | 0.0000 | 0.0 | 712 | 0.080 | 0.0032 | 2.3 | 20 | 0.0004 | 0.3 |
| 1467 | 0.003 | 0.0000 | 0.0 | 3 | 0.0000 | 0.0 | 707 | -0.019 | 0.0002 | 0.1 | 9 | 0.0001 | 0.1 |
| 1465 | -0.024 | 0.0003 | 0.4 | 1 | 0.0000 | 0.0 | 693 | 0.041 | 0.0008 | 0.6 | 12 | 0.0001 | 0.1 |
| 1465 | -0.063 | 0.0020 | 2.9 | 42 | 0.0004 | 0.6 | 686 | -0.038 | 0.0007 | 0.5 | 20 | 0.0004 | 0.3 |
| 1461 | -0.006 | 0.0000 | 0.0 | 97 | 0.0022 | 3.2 | 680 | -0.056 | 0.0015 | 1.1 | 5 | 0.0000 | 0.0 |
| 1459 | -0.005 | 0.0000 | 0.0 | 9 | 0.0000 | 0.0 | 668 | 0.047 | 0.0011 | 0.7 | 35 | 0.0014 | 0.9 |
| 1459 | 0.007 | 0.0000 | 0.0 | 41 | 0.0004 | 0.6 | 657 | 0.043 | 0.0009 | 0.6 | 51 | 0.0030 | 2.0 |
| 1458 | 0.081 | 0.0033 | 4.8 | 1 | 0.0000 | 0.0 | 639 | 0.057 | 0.0016 | 1.0 | 22 | 0.0006 | 0.4 |
| 1444 | 0.026 | 0.0003 | 0.5 | 4 | 0.0000 | 0.0 | 624 | 0.052 | 0.0014 | 0.9 | 14 | 0.0002 | 0.2 |
| 1441 | 0.087 | 0.0037 | 5.4 | 10 | 0.0000 | 0.0 | 593 | -0.091 | 0.0041 | 2.4 | 50 | 0.0035 | 2.1 |
| 1435 | -0.009 | 0.0000 | 0.1 | 59 | 0.0008 | 1.2 | 582 | -0.008 | 0.0000 | 0.0 | 2 | 0.0000 | 0.0 |
| 1432 | -0.001 | 0.0000 | 0.0 | 121 | 0.0036 | 5.1 | 573 | 0.027 | 0.0004 | 0.2 | 7 | 0.0001 | 0.0 |
| 1429 | -0.005 | 0.0000 | 0.0 | 67 | 0.0011 | 1.6 | 561 | 0.029 | 0.0004 | 0.2 | 24 | 0.0009 | 0.5 |
| 1422 | -0.074 | 0.0028 | 3.9 | 38 | 0.0004 | 0.5 | 558 | -0.068 | 0.0023 | 1.3 | 30 | 0.0014 | 0.8 |
| 1402 | -0.025 | 0.0003 | 0.5 | 8 | 0.0000 | 0.0 | 525 | 0.031 | 0.0005 | 0.2 | 25 | 0.0011 | 0.6 |
| 1396 | -0.081 | 0.0033 | 4.6 | 36 | 0.0003 | 0.5 | 513 | -0.075 | 0.0028 | 1.4 | 3 | 0.0000 | 0.0 |
| 1388 | -0.031 | 0.0005 | 0.7 | 4 | 0.0000 | 0.0 | 501 | 0.110 | 0.0060 | 3.0 | 16 | 0.0005 | 0.2 |
| 1384 | -0.018 | 0.0002 | 0.2 | 2 | 0.0000 | 0.0 | 487 | -0.001 | 0.0000 | 0.0 | 45 | 0.0042 | 2.1 |
| 1379 | 0.012 | 0.0001 | 0.1 | 22 | 0.0001 | 0.2 | 470 | 0.111 | 0.0062 | 2.9 | 35 | 0.0028 | 1.3 |
| 1378 | 0.018 | 0.0002 | 0.2 | 0 | 0.0000 | 0.0 | 455 | 0.085 | 0.0036 | 1.6 | 43 | 0.0044 | 2.0 |
| 1375 | 0.018 | 0.0002 | 0.2 | 10 | 0.0000 | 0.0 | 443 | -0.010 | 0.0001 | 0.0 | 58 | 0.0085 | 3.8 |
| 1374 | -0.006 | 0.0000 | 0.0 | 76 | 0.0015 | 2.1 | 434 | 0.038 | 0.0007 | 0.3 | 15 | 0.0006 | 0.3 |
| 1365 | 0.052 | 0.0013 | 1.8 | 29 | 0.0002 | 0.3 | 425 | 0.038 | 0.0007 | 0.3 | 24 | 0.0015 | 0.7 |
| 1362 | -0.017 | 0.0001 | 0.2 | 29 | 0.0002 | 0.3 | 402 | -0.011 | 0.0001 | 0.0 | 20 | 0.0012 | 0.5 |
| 1348 | -0.021 | 0.0002 | 0.3 | 43 | 0.0005 | 0.7 | 391 | -0.040 | 0.0008 | 0.3 | 9 | 0.0003 | 0.1 |
| 1338 | 0.096 | 0.0046 | 6.1 | 45 | 0.0006 | 0.8 | 388 | -0.131 | 0.0085 | 3.3 | 5 | 0.0001 | 0.0 |
| 1331 | -0.007 | 0.0000 | 0.0 | 146 | 0.0060 | 8.0 | 370 | 0.003 | 0.0000 | 0.0 | 16 | 0.0009 | 0.3 |
| 1324 | 0.186 | 0.0172 | 22.8 | 126 | 0.0046 | 6.0 | 357 | 0.074 | 0.0028 | 1.0 | 8 | 0.0002 | 0.1 |
| 1321 | -0.254 | 0.0322 | 42.6 | 115 | 0.0038 | 5.0 | 345 | -0.089 | 0.0040 | 1.4 | 32 | 0.0043 | 1.5 |
| 1312 | -0.041 | 0.0008 | 1.1 | 7 | 0.0000 | 0.0 | 339 | 0.001 | 0.0000 | 0.0 | 12 | 0.0006 | 0.2 |
| 1288 | -0.036 | 0.0007 | 0.9 | 69 | 0.0014 | 1.8 | 316 | 0.024 | 0.0003 | 0.1 | 3 | 0.0000 | 0.0 |
| 1287 | -0.074 | 0.0028 | 3.5 | 129 | 0.0050 | 6.4 | 312 | -0.014 | 0.0001 | 0.0 | 18 | 0.0017 | 0.5 |
| 1286 | -0.004 | 0.0000 | 0.0 | 17 | 0.0001 | 0.1 | 306 | 0.058 | 0.0017 | 0.5 | 8 | 0.0004 | 0.1 |
| 1283 | 0.019 | 0.0002 | 0.2 | 11 | 0.0000 | 0.0 | 298 | -0.061 | 0.0018 | 0.5 | 4 | 0.0001 | 0.0 |

| | | | | | | | | | | | | | |
|---|---|---|---|---|---|---|---|---|---|---|---|---|---|
| 1273 | -0.017 | 0.0001 | 0.2 | 68 | 0.0014 | 1.8 | 294 | -0.074 | 0.0027 | 0.8 | 3 | 0.0001 | 0.0 |
| 1262 | -0.130 | 0.0084 | 10.6 | 35 | 0.0004 | 0.5 | 288 | -0.019 | 0.0002 | 0.0 | 16 | 0.0015 | 0.4 |
| 1259 | -0.002 | 0.0000 | 0.0 | 62 | 0.0012 | 1.5 | 283 | -0.045 | 0.0010 | 0.3 | 4 | 0.0001 | 0.0 |
| 1255 | 0.096 | 0.0046 | 5.8 | 7 | 0.0000 | 0.0 | 280 | -0.033 | 0.0005 | 0.2 | 11 | 0.0007 | 0.2 |
| 1221 | -0.203 | 0.0206 | 25.1 | 181 | 0.0110 | 13.5 | 267 | 0.082 | 0.0034 | 0.9 | 2 | 0.0000 | 0.0 |
| 1211 | -0.238 | 0.0284 | 34.4 | 8 | 0.0000 | 0.0 | 253 | 0.026 | 0.0003 | 0.1 | 22 | 0.0037 | 0.9 |
| 1203 | -0.033 | 0.0006 | 0.7 | 128 | 0.0057 | 6.8 | 249 | -0.016 | 0.0001 | 0.0 | 26 | 0.0053 | 1.3 |
| 1192 | 0.096 | 0.0046 | 5.5 | 167 | 0.0098 | 11.7 | 244 | -0.114 | 0.0065 | 1.6 | 25 | 0.0051 | 1.2 |
| 1184 | 0.032 | 0.0005 | 0.6 | 59 | 0.0012 | 1.5 | 236 | 0.052 | 0.0014 | 0.3 | 7 | 0.0004 | 0.1 |
| 1179 | 0.008 | 0.0000 | 0.0 | 18 | 0.0001 | 0.1 | 230 | 0.048 | 0.0011 | 0.3 | 11 | 0.0011 | 0.3 |
| 1178 | 0.004 | 0.0000 | 0.0 | 14 | 0.0001 | 0.1 | 222 | -0.085 | 0.0036 | 0.8 | 18 | 0.0031 | 0.7 |
| 1174 | -0.004 | 0.0000 | 0.0 | 15 | 0.0001 | 0.1 | 211 | 0.077 | 0.0029 | 0.6 | 8 | 0.0008 | 0.2 |
| 1167 | -0.137 | 0.0094 | 11.0 | 88 | 0.0028 | 3.3 | 202 | 0.007 | 0.0000 | 0.0 | 21 | 0.0055 | 1.1 |
| 1149 | -0.143 | 0.0102 | 11.7 | 10 | 0.0000 | 0.0 | 200 | -0.071 | 0.0025 | 0.5 | 22 | 0.0060 | 1.2 |
| 1145 | -0.014 | 0.0001 | 0.1 | 34 | 0.0004 | 0.5 | 193 | -0.072 | 0.0026 | 0.5 | 49 | 0.0320 | 6.2 |
| 1145 | -0.022 | 0.0002 | 0.3 | 55 | 0.0012 | 1.3 | 188 | -0.025 | 0.0003 | 0.1 | 19 | 0.0049 | 0.9 |
| 1143 | 0.029 | 0.0004 | 0.5 | 68 | 0.0018 | 2.0 | 184 | -0.019 | 0.0002 | 0.0 | 10 | 0.0016 | 0.3 |
| 1136 | -0.051 | 0.0013 | 1.5 | 161 | 0.0100 | 11.4 | 177 | 0.041 | 0.0009 | 0.2 | 3 | 0.0001 | 0.0 |
| 1131 | 0.125 | 0.0078 | 8.8 | 64 | 0.0016 | 1.8 | 173 | -0.059 | 0.0017 | 0.3 | 18 | 0.0052 | 0.9 |
| 1122 | -0.046 | 0.0010 | 1.2 | 99 | 0.0039 | 4.4 | 168 | 0.059 | 0.0018 | 0.3 | 4 | 0.0003 | 0.1 |
| 1114 | -0.059 | 0.0017 | 1.9 | 139 | 0.0078 | 8.7 | 154 | -0.241 | 0.0290 | 4.5 | 16 | 0.0053 | 0.8 |
| 1110 | 0.002 | 0.0000 | 0.0 | 170 | 0.0118 | 13.1 | 149 | -0.001 | 0.0000 | 0.0 | 7 | 0.0012 | 0.2 |
| 1102 | -0.052 | 0.0014 | 1.5 | 306 | 0.0386 | 42.5 | 144 | -0.094 | 0.0044 | 0.6 | 8 | 0.0016 | 0.2 |
| 1093 | -0.028 | 0.0004 | 0.4 | 120 | 0.0060 | 6.6 | 141 | 0.016 | 0.0001 | 0.0 | 1 | 0.0000 | 0.0 |
| 1074 | -0.014 | 0.0001 | 0.1 | 65 | 0.0019 | 2.0 | 136 | 0.129 | 0.0083 | 1.1 | 7 | 0.0014 | 0.2 |
| 1063 | -0.007 | 0.0000 | 0.0 | 93 | 0.0038 | 4.0 | 135 | 0.162 | 0.0132 | 1.8 | 18 | 0.0094 | 1.3 |
| 1059 | 0.050 | 0.0013 | 1.3 | 23 | 0.0002 | 0.3 | 125 | -0.062 | 0.0019 | 0.2 | 9 | 0.0025 | 0.3 |
| 1048 | 0.010 | 0.0001 | 0.1 | 81 | 0.0030 | 3.1 | 114 | -0.063 | 0.0020 | 0.2 | 3 | 0.0003 | 0.0 |
| 1042 | -0.019 | 0.0002 | 0.2 | 286 | 0.0376 | 39.2 | 106 | -0.064 | 0.0020 | 0.2 | 2 | 0.0001 | 0.0 |
| 1040 | -0.052 | 0.0014 | 1.4 | 138 | 0.0088 | 9.2 | 99 | -0.159 | 0.0126 | 1.2 | 8 | 0.0034 | 0.3 |
| 1039 | -0.009 | 0.0000 | 0.0 | 27 | 0.0003 | 0.4 | 93 | -0.339 | 0.0574 | 5.3 | 28 | 0.0457 | 4.3 |
| 1039 | -0.021 | 0.0002 | 0.2 | 7 | 0.0000 | 0.0 | 90 | -0.016 | 0.0001 | 0.0 | 10 | 0.0056 | 0.5 |
| 1035 | -0.084 | 0.0035 | 3.7 | 4 | 0.0000 | 0.0 | 89 | -0.129 | 0.0083 | 0.7 | 10 | 0.0064 | 0.6 |
| 1028 | 0.028 | 0.0004 | 0.4 | 119 | 0.0067 | 6.9 | 78 | 0.007 | 0.0000 | 0.0 | 9 | 0.0071 | 0.6 |
| 1024 | 0.057 | 0.0016 | 1.6 | 71 | 0.0024 | 2.5 | 75 | -0.086 | 0.0037 | 0.3 | 15 | 0.0192 | 1.4 |
| 1015 | -0.086 | 0.0037 | 3.7 | 3 | 0.0000 | 0.0 | 64 | -0.337 | 0.0569 | 3.6 | 20 | 0.0470 | 3.0 |
| 990 | 0.021 | 0.0002 | 0.2 | 59 | 0.0018 | 1.8 | 61 | -0.074 | 0.0028 | 0.2 | 13 | 0.0225 | 1.4 |
| 986 | -0.089 | 0.0040 | 3.9 | 107 | 0.0059 | 5.8 | 54 | -0.146 | 0.0107 | 0.6 | 9 | 0.0140 | 0.8 |
| 977 | 0.282 | 0.0398 | 38.8 | 60 | 0.0019 | 1.8 | 47 | 0.109 | 0.0060 | 0.3 | 7 | 0.0118 | 0.6 |
| 963 | -0.104 | 0.0054 | 5.2 | 35 | 0.0007 | 0.6 | 41 | 0.618 | 0.1910 | 7.8 | 19 | 0.1068 | 4.4 |
| 960 | 0.012 | 0.0001 | 0.1 | 24 | 0.0003 | 0.3 | 38 | 0.394 | 0.0774 | 2.9 | 8 | 0.0205 | 0.8 |
| 957 | -0.049 | 0.0012 | 1.1 | 48 | 0.0013 | 1.2 | 27 | -0.290 | 0.0421 | 1.1 | 44 | 1.3359 | 36.1 |
| 935 | 0.127 | 0.0081 | 7.5 | 10 | 0.0001 | 0.1 | 22 | -0.035 | 0.0006 | 0.0 | 33 | 1.1402 | 25.1 |
| 917 | -0.010 | 0.0001 | 0.0 | 5 | 0.0000 | 0.0 | 18 | -0.069 | 0.0024 | 0.0 | 20 | 0.6468 | 11.6 |
| 908 | 0.146 | 0.0106 | 9.6 | 37 | 0.0008 | 0.8 | 16 | 0.467 | 0.1092 | 1.7 | 57 | 6.2382 | 99.8 |
| 898 | 0.003 | 0.0000 | 0.0 | 76 | 0.0036 | 3.2 | 13 | -0.408 | 0.0832 | 1.1 | 42 | 5.2698 | 68.5 |
| | | | | | | | **Total** | | **1.19** | **505** | | **15.4** | **615** |

**S7. DFT Calculations of the spectral properties of dimeric and trimeric complexes of solvent molecules with chlorophyllides.**

Figure 5 of the main text compares the experimentally deduced unperturbed $Q_x$-$Q_y$ energy gaps $\Delta E$ and total fraction of *x*-polarized Q-band absorptions $f_x$ to values calculated by CAM-B3LYP. The calculated values were obtained considering only clusters of chlorophyllides with either one or two solvent molecules ligated to the central metal, or, for free-base molecules, considering only the molecule itself. This treatment ignores long-range solvation effects, but such effects are known to be relatively small for the properties of interest and to vary only slightly with solvent.[48] The B3LYP density-functional[49] was used to optimize all geometries in conjunction with the 6-31G* basis set[43] for C, H, N, O, and Mg and LANL2DZ[50] for Co, Ni, Cu, and Zn, while CAM-B3LYP[44] single-point excited-state energies are reported at these geometries; all calculations were performed using a GAUSSIAN Development Version[47] extended by us[51] to include CAM-B3LYP. The ground-state optimized geometries for the molecules and clusters listed in Table S6 are provided in associated file "Cartersian_Coodrinates.zip".

Calculated vertical excitation energy differences (for clusters in the gas phase) $\Delta E$ and observed values (in solution) are compared in Table S7 and graphed in Fig. 5a. Agreement is generally good but a systematic error between free-base and metallated species is apparent. The correlations drawn in Fig. 5a have very similar slopes of 1.27 and 1.24 for free-base and metallated species, but the two lines are offset by 1330 $cm^{-1}$.

Note that not all of observed values are taken from our vibronic-coupling assignments, however, with insufficient experimental data being available to facilitate this approach for many important samples. Hence some data taken from observed sub-band maxima in the limit of large $\Delta E$ are also included; application of the vibronic-coupling correction for these systems would increase the deduced experimental value for $\Delta E$ by a small amount. In Fig. 5, raw data is represented by open circles whilst vibronically corrected data is represented by filled circles. An improved vibronic-coupling approach in which individual vibronic-coupling parameters are fitted to every individual chlorophyllide would also result in some small changes to the experimental values.

To improve the comparison between observed and calculated data, comparisons of quantities that negate systematic errors in both sets of data are required. A significant spectroscopic signature of interest is the change from 5CO to 6CO in the induced $Q_x$-$Q_y$ gap $\Delta\Delta E$, a property that is much less sensitive to both experimental and computational errors. Observed and calculated values are compared in Table S8 and in Fig. 5b, with the observed data presented as either the "traditional" or "modern" assignments based on sub-band maxima or else our full vibronic-coupling assignment. The vibronic-coupling assignment is in excellent agreement with the calculated data while the "traditional" and "modern" assignments agree only for molecules with large $\Delta E$. This data includes results for 6CO chlorophyllides in ether at low temperature for which an observed signal is only obtained when trace amounts of water are present in the solvent and hence these samples are modelled as water complexes rather than as ether complexes.[3]

Also, Table S9 gives the fraction of *Q*-band intensity apportioned to $Q_x$ as calculated by CAM-B3LYP and as deduced from the vibronic-coupling model (this data is graphed in Fig. 5c). The factor-of-five variation in $Q_x$ intensity deduced from the spectra is paralleled by the calculations. Going from 5CO to 6CO, the largest observed change in $Q_x$ intensity is for Chl-a in ether for which the intensity doubles. The calculations for the both the doubly hydrated species Chl-a.water.water and the monohydrate Chl-a.ether.water reproduce the observed effect.[3] Note that the $Q_x$ oscillator strength is

found to be very sensitive to the angle between the symmetry axis of the water and the Mg-O vector, with results obtained for a tilted angle expected if the water molecule hydrogen-bonded to its environment[3] being used in Fig. 5c.

**Table S6.** Molecules and molecular clusters for which B3LYP/6-31G* optimized gas-phase coordinates are given in SI dataset "Cartesian_Coordinates.xls"; prop= 1-propanol, iprop= 2-propanol.

| | | | |
|---|---|---|---|
| acetone | BChl-d_prop | Chl-a_Co_diethylether | Chl-c2_2diethylether |
| Bchl-a | BChl-d_pyridine | Chl-a_Cu | Chl-c2_2prop |
| BChl-a_2acetone | BChl-e | Chl-a_Cu_2diethylether | Chl-c2_2pyridine |
| BChl-a_2diethylether | BChl-e_2diethylether | Chl-a_Cu_diethylether | Chl-c2_diethylether |
| BChl-a_2iprop | BChl-e_2prop | Chl-a_diethylether | Chl-c2_prop |
| BChl-a_2prop | BChl-e_2pyridine | Chl-a_ethanol | Chl-c2_pyridine |
| BChl-a_2pyridine | BChl-e_diethylether | Chl-a_iprop | Chl-c3 |
| BChl-a_2water | BChl-e_prop | Chl-a_Ni | Chl-c3_2diethylether |
| BChl-a_acetone | BChl-e_pyridine | Chl-a_Ni_2diethylether | Chl-c3_2prop |
| BChl-a_diethylether | BChl-f | Chl-a_Ni_diethylether | Chl-c3_2pyridine |
| BChl-a_iprop | BChl-f_2diethylether | Chl-a_prop | Chl-c3_diethylether |
| BChl-a_prop | BChl-f_2prop | Chl-a_pyridine | Chl-c3_prop |
| BChl-a_pyridine | BChl-f_2pyridine | Chl-a_water | Chl-c3_pyridine |
| BChl-a_water | BChl-f_diethylether | Chl-a_water_diethylether | Chl-d |
| BChl-a_water_diethylether | BChl-f_prop | Chl-a_water_diethylether_tilted | Chl-d_2diethylether |
| BChl-b | BChl-f_pyridine | Chl-a_Zn | Chl-d_2methanol |
| BChl-b_2diethylether | BChl-g | Chl-a_Zn_2acetone | Chl-d_2prop |
| BChl-b_2prop | BChl-g_2diethylether | Chl-a_Zn_2diethylether | Chl-d_2pyridine |
| BChl-b_2pyridine | BChl-g_2prop | Chl-a_Zn_acetone | Chl-d_diethylether |
| BChl-b_diethylether | BChl-g_2pyridine | Chl-a_Zn_acetone_2 | Chl-d_methanol |
| BChl-b_prop | BChl-g_diethylether | Chl-a_Zn_diethylether | Chl-d_prop |
| BChl-b_pyridine | BChl-g_prop | Chl-b | Chl-d_pyridine |
| BChl-c_2acetone | BChl-g_pyridine | Chl-b_2diethylether | diethylether |
| BChl-c_2diethylether | BPheo-a | Chl-b_2ethanol | ethanol |
| BChl-c_2prop | BPheo-b | Chl-b_2prop | iprop |
| BChl-c_2pyridine | BPheo-g | Chl-b_2pyridine | Pheo-a |
| BChl-c_acetone | Chl-a | Chl-b_diethylether | Pheo-b |
| BChl-c_diethylether | Chl-a_2acetone | Chl-b_ethanol | Pheo-d |
| BChl-c_prop | Chl-a_2diethylether | Chl-b_prop | porphyrin |
| BChl-c_pyridine | Chl-a_2ethanol | Chl-b_pyridine | prop |
| BChl-c1 | Chl-a_2iprop | Chl-c1 | Protochla |
| BChl-d | Chl-a_2prop | Chl-c1_2diethylether | Protochla_2diethylether |
| BChl-d_2acetone | Chl-a_2pyridine | Chl-c1_2prop | Protochla_diethylether |
| BChl-d_2diethylether | Chl-a_2water | Chl-c1_2pyridine | ProtoPheo-a |
| BChl-d_2prop | Chla_2water_tilted | Chl-c1_diethylether | ProtoPheoa_usual_H |
| BChl-d_2pyridine | Chl-a_acetone | Chl-c1_prop | pyridine |
| BChl-d_acetone | Chl-a_Co | Chl-c1_pyridine | |
| BChl-d_diethylether | Chl-a_Co_2diethylether | Chl-c2 | |

**Table S7.** CAM-B3LYP calculated gas-phase $Q_x$-$Q_y$ energy gaps $\Delta E$ , in 1000 $cm^{-1}$, for 5CO and 6CO clusters compared to the "traditional", "modern", and our vibronic-coupling assignments in solution, plus some raw unassigned peak maxima, observed in solution (plotted in Fig. 3a).

| Cluster (solution) | CAM-B3LYP | Trad. | Modern | Vib. Coup. | Raw Peak |
|---|---|---|---|---|---|
| Pheo-a (EtOH/MeOH 1.7 K)[a] | 2.900 | 3.606 | 3.606 | 3.38 | |
| Pheo-b (benzene)[b] | 2.561 | | | | 2.61 |
| Pheo-d (EtOH/MeOH 1.7 K)[a] | 2.909 | 3.71 | 3.71 | 3.48 | |
| BPheo-a (ether)[cm] | 4.207 | | | | 5.71 |
| BPheo-b (ether)[c] | 4.513 | | | | 5.89 |
| BPheo-g (ether)[d] | 4.430 | | | | 5.80 |
| Protopheophytin-a (ether)[n] | -1.300 | | | | -1.24 |
| Protochlorophyll-a.ether (ether)[n] | 0.410 | | | | 0.53 |
| Tetrameso(3,5-di-*t*-butyl) porphyrin (ether)[e] | -1.855 | | | | -2.90 |
| Chl-a.ether (ether)[f] | 2.637 | 2.169 | 1.042 | 1.64 | |
| Chl-b.ether (MBBA+EBBA+DAB)[g] | 2.325 | 0.945 | 0.945 | 1.31 | |
| Chl-c1.ether (1% pyridine in ether)[o] | 0.280 | | | | -0.94 |
| Chl-c2.ether (1% pyridine in ether)[o] | 0.178 | | | | -0.97 |
| Chl-c3.ether (1% pyridine in ether)[o] | -0.137 | | | | -1.00 |
| BChl-a.ether (ether)[h] | 4.069 | 4.323 | 4.323 | 4.14 | |
| BChl-a.1-propanol (1-propanol)[i] | 3.994 | | | | 3.73 |
| BChl-a.2(1-propanol) (1-propanol)[i] | 3.298 | | | | 3.10 |
| BChl-b.ether (ether)[c] | 3.776 | | | | 4.25 |
| BChl-c.ether (ether)[f] | 2.083 | 1.756 | 0.903 | 1.15 | |
| BChl-d.ether (ether)[j] | 2.477 | 1.968 | 1.010 | 1.38 | |
| BChl-g.1-propanol (hexane/1-propanol) | 4.423 | | | | 4.64 |
| Chl-a.pyridine (pyridine)[f] | 1.909 | 1.65 | 0.697 | 0.97 | |
| Chl-a.water.? (wet ether 156 K) | 2.055 | | | 1.02 | |
| Chl-a 1-propanol 1.8 K 6CO[k] | 2.643 | | | 0.82 | |
| Chl-a 1-propanol 1.8 K 5CO[k] | 2.623 | | | 1.20 | |
| Chl-a 1-propanol 1.8 K 6CO[k] | 2.020 | | | 0.68 | |
| Chl-d.1-propanol (EtOH/MeOH 1.7 K)[kp] | 1.778 | 1.533 | 0.72 | 0.81 | |
| BChl-a.2pyridine (pyridine)[h] | 3.071 | 3.445 | 3.445 | 3.24 | |
| BChl-c.2pyridine (pyridine)[f] | 1.328 | 1.538 | 0.273 | 0.44 | |
| BChl-d.2pyridine (pyridine)[j] | 1.762 | 1.227 | 0.539 | 0.71 | |
| Ni(II)-Chl-a.ether (ether)[l] | 3.511 | 1.40 | 3.10 | 2.75 | |
| Co(II)-Chl-a.ether (ether)[l] | 3.136 | 0.91 | 2.87 | 2.23 | |
| Cu(II)-Chl-a.ether (ether)[l] | 2.982 | 1.41 | 3.02 | 2.64 | |
| Zn(II)-Chl-a.ether (ether)[l] | 2.719 | 1.19 | 2.42 | 1.99 | |

a: Razeghifard,[35] calculations for 1-propanol; b: Watanabe,[52] c: Oelze;[53] d: Kunieda;[54] e: Cai[55,56] (from MCD at 65 K, $\alpha$~ 620 $cm^{-1}$); f: Umetsu;[20] g: Frackowiak;[36] h: Umetsu;[32] i: Bellacchio;[57] j: Umetsu;[31] k: this work; l: Nonomura;[33] m: Hartwich;[58] n: Houssier,[59] the negative value for protopheophytin-a arises from inner-hydrogen rotation, see supplied Cartesian Coordinates; o: Helfrich[60] revised assignment with $Q_x$ & $Q_y$ at 608 nm & 575 nm, 608 nm & 574 nm, 618 & 582 nm for Chl-c1, Chl-c2, and Chl-c3, respectively.; p: calculations for 1-propanol.

**Table S8.** Comparison of CAM-B3LYP calculated changes $\Delta\Delta E$ in $Q_x$-$Q_y$ energy gaps, in 1000 $cm^{-1}$, between 5CO and 6CO species, with those from the traditional, modern, and vibronic-coupling assignments (see Fig. 3b).

| chlorophyllide | 5CO | 6CO | CAM-B3LYP | Trad. | Modern | Vib. Coup. |
|---|---|---|---|---|---|---|
| Chl-a | ether | pyridine | -0.728 | -1.47 | -0.35 | -0.67 |
| BChl-a | ether | pyridine | -0.998 | -0.90 | -0.90 | -0.90 |
| BChl-c | ether | pyridine | -0.756 | -1.48 | -0.63 | -0.71 |
| BChl-d | ether | pyridine | -0.715 | -1.43 | -0.47 | -0.67 |
| Chl-a | ether | ether.water | -0.582 | -1.57 | -0.44 | -0.56 |
| Chl-a | 2-propanol | 2-propanol | -0.603 | | | -0.52 |
| BChl-a | pyridine | pyridine | -0.826 | -0.73 | -0.73 | -0.73[a] |
| BChl-a | 1-propanol | 1-propanol | -0.696 | -0.63 | -0.63 | -0.63[b] |

a: Evans;[61] b: Bellacchio.[57]

**Table S9.** Comparison of CAM-B3LYP calculated fraction of $Q$-band absorption attributed to $Q_x$, $f_x$ for gas-phase clusters and results observed in solution based on the traditional, modern, and vibronic-coupling assignments (plotted in Fig. 3c).

| Cluster | CAM-B3LYP | Trad. | Modern | Vib. Coup. |
|---|---|---|---|---|
| Chl-a.ether | 0.11 | 0.097 | 0.037 | 0.12 |
| Chl-a.H2O tilted at 30° (~$sp^3$) | 0.17 | | | |
| Chl-a.2H2O optimized flat | 0.14 | | | |
| Chl-a.2H2O tilted at 30° (~$sp^3$) | 0.20 | | | 0.25 |
| Chl-a.2pyridine | 0.16 | 0.065 | 0.158 | 0.17 |
| Chl-a.2(1-propanol) | 0.15 | | | 0.19 |
| Chl-a.(2-propanol) | 0.10 | | | 0.11 |
| Chl-a.2(2-propanol) | 0.15 | | | 0.19 |
| BChl-a.ether | 0.17 | 0.19 | 0.19 | 0.21 |
| BChl-a.2pyridine | 0.19 | 0.22 | 0.22 | 0.22 |
| BChl-c.ether | 0.17 | 0.089 | 0.113 | 0.16 |
| BChl-c.2pyridine | 0.22 | 0.057 | 0.260 | 0.22 |
| BChl-d.ether | 0.10 | 0.093 | 0.057 | 0.10 |
| BChl-d.2pyridine | 0.16 | 0.082 | 0.143 | 0.17 |
| tetrameso-(di-*t*-butylphenyl)porphyrin | 0.29 | 0.29 | 0.29 | 0.33 |
| Pheo-a | 0.11 | 0.13 | 0.13 | 0.13 |
| Pheo-d | 0.13 | 0.15 | 0.15 | 0.15 |
| Ni(II)-Chl-a.ether | 0.056 | 0.084 | 0.040 | 0.067 |
| Co(II)-Chl-a.ether | 0.070 | 0.116 | 0.050 | 0.085 |
| Cu(II)-Chl-a.ether | 0.092 | 0.089 | 0.080 | 0.073 |
| Zn(II)-Chl-a.ether | 0.090 | 0.097 | 0.037 | 0.093 |

## S8. $Q_x \rightarrow Q_y$ relaxation calculations.

The method used to estimate the rate of $Q_x \rightarrow Q_y$ relaxation follows the general ansatz of Reimers and Hush.[62] Intramolecular relaxation on $Q_y$ after transfer of a coherent wavepacket from $Q_x$ is modelled using a single parameter $\rho$ representing the Franck-Condon weighted coupled density of states at the energy of the $Q_y$ origin plus that of the coupling mode $\nu_{vc}$. An imaginary energy contribution of $i/\rho$ is then added to every level of the $Q_y$ state in the vibronic-coupling Hamiltonian **H** (Eq. S10) that has vibrational excitation in the vibronically coupled mode. The time-dependent Schrodinger equation is then solved for an initial wavefunction representing excitation to the unperturbed $Q_x$ origin, yielding $\left|\Psi(t)\right\rangle$. The norm of this wavefunction decays with time owing to the vibrational relaxation that occurs on $Q_y$, and the lifetime for this process is determined as[62]

$$\tau = \int_0^\infty \left\langle \Psi(t) \middle| \Psi(t) \right\rangle \mathrm{d}t \, .$$

## S9. SI References


1 Avarmaa, R. A. & Rebane, K. K. High-resolution optical spectra of chlorophyll molecules. *Spectrochim. Acta A* **41**, 1365-1380 (1985).

2 Rätsep, M., Linnanto, J. & Freiberg, A. Mirror symmetry and vibrational structure in optical spectra of chlorophyll a. *J. Chem. Phys.* **130**, 194501 (2009).

3 Reimers, J. R. *et al.* Formation of water-chlorophyll clusters in dilute samples of chlorophyll-a in ether at low temperature. *PCCP* **submitted** (2013).

4 Avarmaa, R. A. & Suisalu, A. *Optics Spectrosc.* **56**, 54-59 (1984).

5 Hughes, J. L., Conlon, B., Wydrzynski, T. & Krausz, E. The assignment of Qy(1,0) vibrational structure and Qx for chlorophyll a. *Phys. Procedia* **3**, 1591-1599 (2010).

6 Gillie, J. K., Small, G. J. & Golbeck, J. H. Nonphotochemical hole burning of the native antenna complex of photosystem I (PSI-200). *J. Phys. Chem.* **93**, 1620-1627 (1989).

7 Pieper, J. *et al.* Excitonic energy level structure and pigment−protein interactions in the recombinant water-soluble chlorophyll protein. II. Spectral hole-burning experiments. *J. Phys. Chem. B* **115**, 4053-4065 (2011).

8 Pieper, J. *et al.* Excitonic energy level structure and pigment−protein interactions in the recombinant water-soluble chlorophyll protein. I. Difference fluorescence fine-narrowing. *J. Phys. Chem. B* **115**, 4042-4052 (2011).

9 Fulton, R. L. & Gouterman, M. Vibronic Coupling. I. Mathematical Treatment for Two Electronic States. *J. Chem. Phys.* **35**, 1059-1071 (1961).

10 Fulton, R. L. & Gouterman, M. Vibronic Coupling. II. Spectra of Dimers. *J. Chem. Phys.* **41**, 2280 (1964).

11 Ziegler, L. & Albrecht, A. C. Vibronic calculations in benzene by CNDO/S. *The Journal of Chemical Physics* **60**, 3558-3561 (1974).

12 Ross, I. G. VIBRATIONAL ELECTRONIC COUPLING AND A CLOSE LOOK AT A SEVERE CASE. *Israel Journal of Chemistry* **14**, 118-123 (1975).

13 Piepho, S. B., Krausz, E. R. & Schatz, P. N. Vibronic coupling model for calculation of mixed valence absorption profiles. *J. Am. Chem. Soc.* **100**, 2996-3005 (1978).

14 Chappell, P. J., Fischer, G., Reimers, J. R. & Ross, I. G. Electronic spectrum of 1,5-naphthyridine: theoretical calculation of vibronic coupling. *J. Molec. Spectrosc.* **87**, 316 (1981).

15 Köppel, H., Domcke, W. & Cederbaum, L. S. *Advances in Chemical Physics* **57**, 59-246

(1984).
16 Fischer, G. *Vibronic Coupling*. (Academic Press, 1984).
17 Reimers, J. R. & Hush, N. S. Hamiltonian operators including both symmetric and antisymmetric vibrational modes for vibronic-coupling and intervalence charge-transfer applications. *Chem. Phys.* **299**, 79 (2004).
18 Reimers, J. R., Wilson, K. R. & Heller, E. J. Complex time dependent wave packet technique for thermal equilibrium systems: Electronic spectra. *J. Chem. Phys.* **79**, 4749 (1983).
19 Buckingham, A. D. & Stephens, P. J. Magnetic Optical Activity. *Ann. Rev. Phys. Chem.* **17**, 399-432 (1966).
20 Umetsu, M., Wang, Z.-Y., Kobayashi, M. & Nozawa, T. Interaction of photosynthetic pigments with various organic solvents: magnetic circular dichroism approach and application to chlorosomes. *Biochim. Biophys. Acta, Bioenerg.* **1410**, 19-31 (1999).
21 Schatz, P. N. & McCaffery, A. J. The Faraday effect. *Quarterly Reviews, Chemical Society* **23**, 552-584 (1969).
22 Henry, C. H., Schnatterly, S. E. & Slichter, C. P. Effect of Applied Fields on the Optical Properties of Color Centers. *Physical Review Letters* **13**, 130-132 (1964).
23 Stephens, P. J. Magnetic Circular Dichroism. *Annual Review of Physical Chemistry* **25**, 201-232 (1974).
24 Reimers, J. R. & Krausz, E. An analytical data inversion method for Magnetic Circular Dichroism spectra dominated by the "B-term". *PCCP* **submitted** (2013).
25 Stranger, R., Dubicki, L. & Krausz, E. Magneto-optical investigation of the exchange-coupled dimer Cs3Mo2Br9. *Inorg. Chem.* **35**, 4218-4226 (1996).
26 Hughes, J. L. & Krausz, E. in *Application of Physical Methods to Inorganic and Bioinorganic Chemistry* (eds Robert A. Scott & Charles M. Lukehart) (Wiley, 2007).
27 Linnanto, J., Oksanen, J. A. I. & Korppi-Tommola, J. E. I. Exciton interactions in self-organised bacteriochlorophyll a - aggregates. *Physical Chemistry Chemical Physics* **4**, 3061-3070 (2002).
28 Linnanto, J. *et al.* Exciton Interactions and Femtosecond Relaxation in Chlorophyll a−Water and Chlorophyll a−Dioxane Aggregates. *The Journal of Physical Chemistry A* **102**, 4337-4349 (1998).
29 Hughes, J. L., Pace, R. J. & Krausz, E. The exciton contribution to the Faraday B term MCD of molecular dimers. *Chem. Phys. Lett.* **385**, 116-121 (2004).
30 Rebane, K. K. & Avarmaa, R. A. Sharp line vibronic spectra of chlorophyll and its derivatives in solid solutions. *Chem. Phys.* **68**, 191-200 (1982).
31 Umetsu, M., Wang, Z.-Y. & Nozawa, T. A new approach of magnetic circular dichroism to the electronic state analysis of intact photosynthetic pigments. *Recent Res. Dev. Phys. Chem.* **5**, 185-208 (2001).
32 Umetsu, M., Wang, Z.-Y., Yoza, K., Kobayashi, M. & Nozawa, T. Interaction of photosynthetic pigments with various organic solvents 2. Application of magnetic circular dichroism to bacteriochlorophyll a and light-harvesting complex 1. *Biochimica. Biophysica. Acta Bioenerg.* **1457**, 106-117 (2000).
33 Nonomura, Y., Igarashi, S., Yoshioka, N. & Inoue, H. Spectroscopic properties of chlorophylls and their derivatives. Influence of molecular structure on the electronic state. *Chem. Phys.* **220**, 155-166 (1997).
34 Briat, B., Schooley, D. A., Records, R., Bunnenberg, E. & Djerassi, C. Magnetic circular dichroism studies. III. Investigation of some optically active chlorins. *J. Am. Chem. Soc.* **89**, 6170-6177 (1967).
35 Razeghifard, M. R. *et al.* Spectroscopic Studies of Photosystem II in Chlorophyll d-Containing Acaryochloris marina. *Biochemistry* **44**, 11178-11187 (2005).

36 Frackowiak, D., Bauman, D., Manikowski, H., Browett, W. R. & Stillman, M. J. Circular dichroism and magnetic circular dichroism spectra of chlorophylls a and b in nematic liquid crystals. II. Magnetic circular dichroism spectra. *Biophys. Chem.* **28**, 101-114 (1987).
37 Weiss, C., Jr. π-Electron structure and absorption spectra of chlorophylls in solution. *J. Mol. Spectrosc.* **44**, 37-80 (1972).
38 Norden, B., Fragata, M. & Kurucsev, T. X- and Y-polarized spectra of chlorophyll a and pheophytin a in the red region: resolution enhancement and Gaussian deconvolution. *Aust. J. Chem.* **45**, 1559-1570 (1992).
39 Fragata, M., Norden, B. & Kurucsev, T. Linear dichroism of Chl-a and Pheo-a oriented in a lamellar phase: characterization of electronic transitions. *Photochem. Photobio.* **47**, 133-143 (1988).
40 Lakowicz, J. R. *Principles of Fluorescence Spectroscopy*. Vol. 1 (Springer, 2006).
41 König, C. & Neugebauer, J. Quantum Chemical Description of Absorption Properties and Excited-State Processes in Photosynthetic Systems. *ChemPhysChem* **13**, 386-425 (2012).
42 Frisch, M. J. *et al. Gaussian 09, Revision A.02*. (Gaussian, Inc., Pittsburgh PA, 2009).
43 Hehre, W. J., Ditchfield, R. & Pople, J. A. Self-consistent molecular orbital methods. XII. Further extensions of gaussian-type basis sets for use in molecular orbital studies of organic molecules *J. Chem. Phys.* **56**, 2257-2261 (1972).
44 Yanai, T., Tew, D. P. & Handy, N. C. A new hybrid exchange-correlation functional using the Coulomb-attenuating method (CAM-B3LYP). *Chem. Phys. Lett.* **393**, 51-57 (2004).
45 Reimers, J. R. A practical method for the use of curvilinear coordinates in calculations of normal-mode projected displacements and Duschinsky rotation matrices for large molecules. *J. Chem. Phys.* **115**, 9103-9109 (2001).
46 Rätsep, M., Cai, Z.-L., Reimers, J. R. & Freiberg, A. Demonstration and interpretation of significant asymmetry in the low-resolution and high-resolution Qy fluorescence and absorption spectra of bacteriochlorophyll a. *J. Chem. Phys.* **134**, 024506/024501-024515 (2011).
47 Frisch, M. J. *et al. GAUSSIAN Development Version Rev. E.02*. (Gaussian Inc., 2004).
48 Qu, Z.-w., Zhu, H., May, V. & Schinke, R. Time-Dependent Density Functional Theory Study of the Electronic Excitation Spectra of Chlorophyllide a and Pheophorbide a in Solvents. *J. Phys. Chem. B* **113**, 4817-4825 (2009).
49 Becke, A. D. Density-functional thermochemistry. III. The role of exact exchange. *J. Chem. Phys.* **98**, 5648-5652 (1993).
50 Hay, P. J. & Wadt, W. R. *J. Chem. Phys.* **82**, 270 284 299 (1985).
51 Kobayashi, R. & Amos, R. D. The application of CAM-B3LYP to the charge-transfer band problem of the zincbacteriochlorin-bacteriochlorin complex. *Chem. Phys. Lett.* **420**, 106-109 (2006).
52 Watanabe, T. *et al.* Preparation of chlorophylls and pheophytins by isocratic liquid chromatography. *Anal. Chem.* **56**, 251-256 (1984).
53 Oelze, J. Analysis of bacteriochlorophylls. *Methods Microbiol.* **18**, 257-284 (1985).
54 Kunieda, M., Mizoguchi, T. & Tamiaki, H. Syntheses and optical properties of stable 8-alkylidene-bacteriochlorins mimicking the molecular structures of natural bacteriochlorophylls-b and g. *Tetrahedron* **60**, 11349-11357 (2004).
55 Cai, Z.-L., Sendt, K. & Reimers, J. R. Failure of time-dependent density-functional theory for large extended pi systems. *J. Chem. Phys.* **117**, 5543-5549 (2002).
56 Sendt, K. *et al.* Switchable electronic coupling in oligoporphyrin molecular wires examined through the measurement and assignment of electronic absorption spectra. *J. Am. Chem. Soc.* **124**, 9299-9309 (2002).
57 Bellacchio, E. & Sauer, K. Temperature dependence of optical spectra of bacteriochlorophyll *a*

in solution and in low-temperature glass. *J. Phys. Chem. B* **103**, 2279-2290 (1999).

58 Hartwich, G. *et al.* Metal-substituted bacteriochlorophylls. 1. Preparation and influence of metal and coordination on spectra. *J. Am. Chem. Soc.* **120**, 3675-3683 (1998).

59 Houssier, C. & Sauer, K. Circular dichroism and magnetic circular dichroism of the chlorophyll and protochlorophyll pigments. *J. Amer. Chem. Soc.* **92**, 779-791 (1970).

60 Helfrich, M. *et al.* Chlorophylls of the c family: absolute configuration and inhibition of NADPH:protochlorophyllide oxidoreductase. *Biochim. Biophys. Acta Bioenerg.* **1605**, 97-103 (2003).

61 Evans, T. A. & Katz, J. J. Evidence for 5- and 6-coordinated magnesium in bacteriochlorophyll a from visible absorption spectroscopy. *Biochim. Biophys. Acta, Bioenerg.* **396**, 414-426 (1975).

62 Reimers, J. R. & Hush, N. S. Electron and energy transfer through bridged systems. I. Formalism. *Chem. Phys.* **134**, 323 (1989).

**S10. Detailed fits to MCD and ABS spectra in Fig.1 and Fig. S1.**

The following figures, one per page, show using dots the observed MCD (top) and ABS (bottom) spectra and their fit to sums of spectral components: black- total fit, colours- each individual component. The pages are, in order, for Fig. 1a-l and then for Fig. S1a-q.

Fig. 1a: BChl-a ether

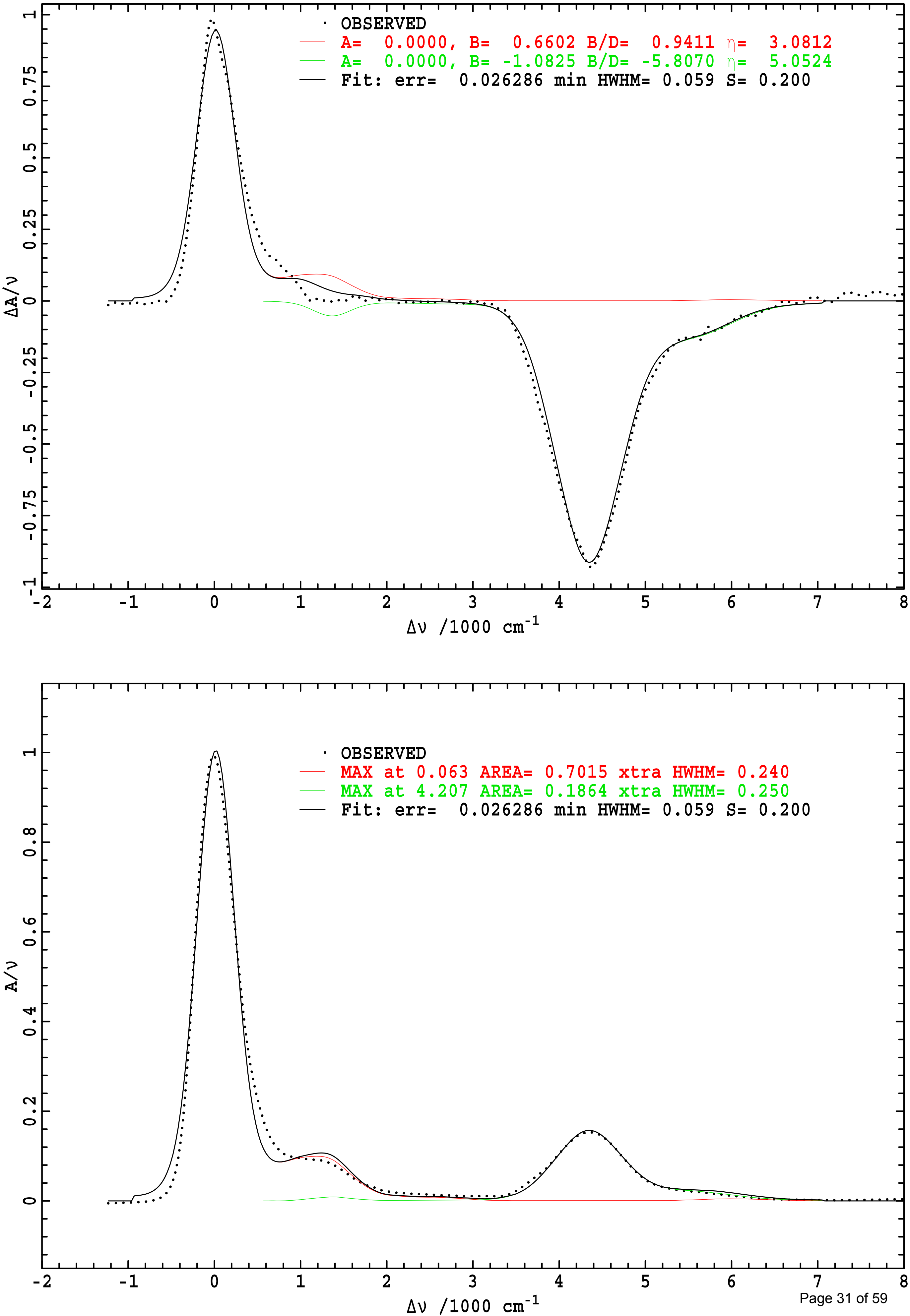

Fig. 1b: Pheo-a EtOH/MeOH 1.7 K

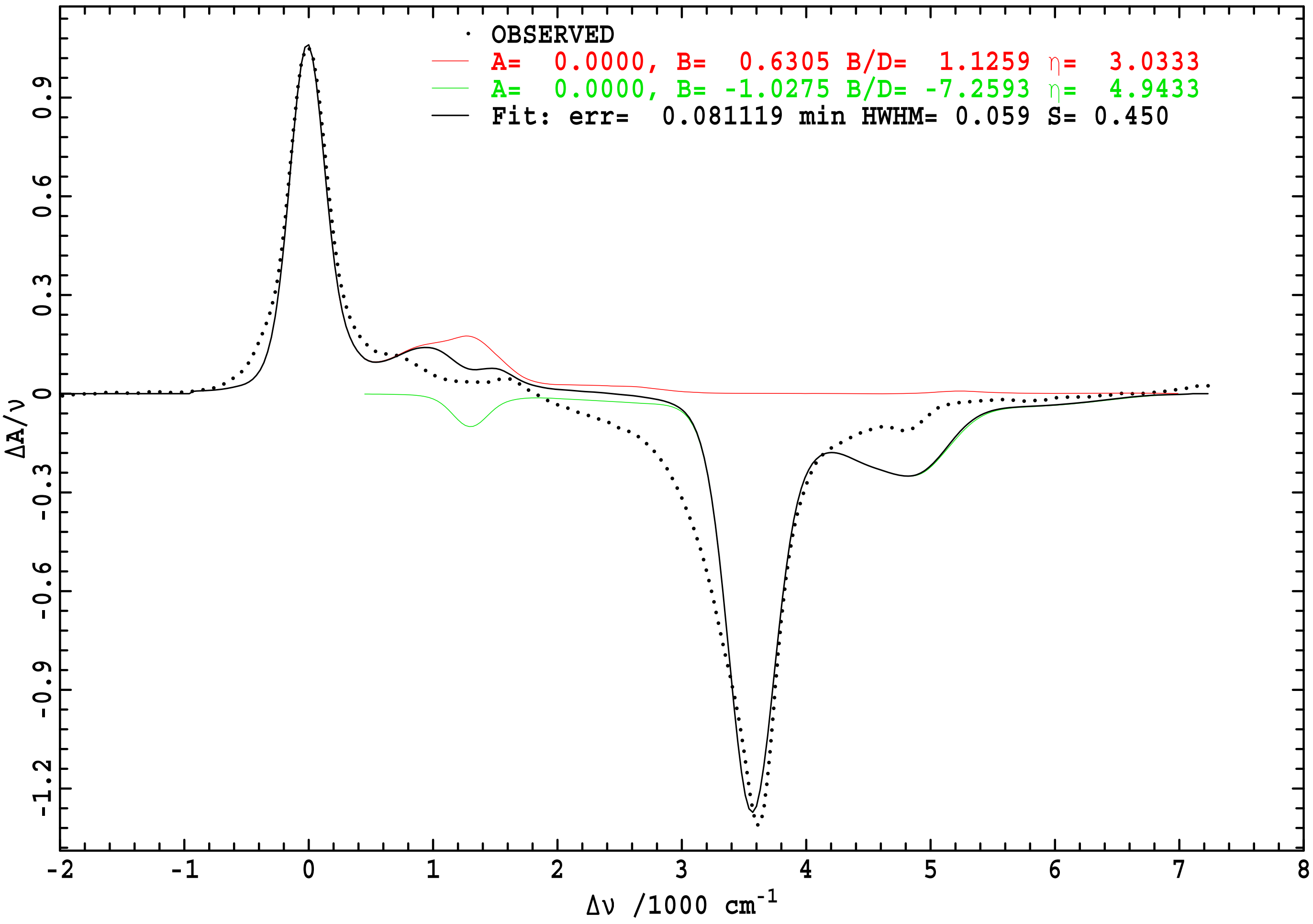


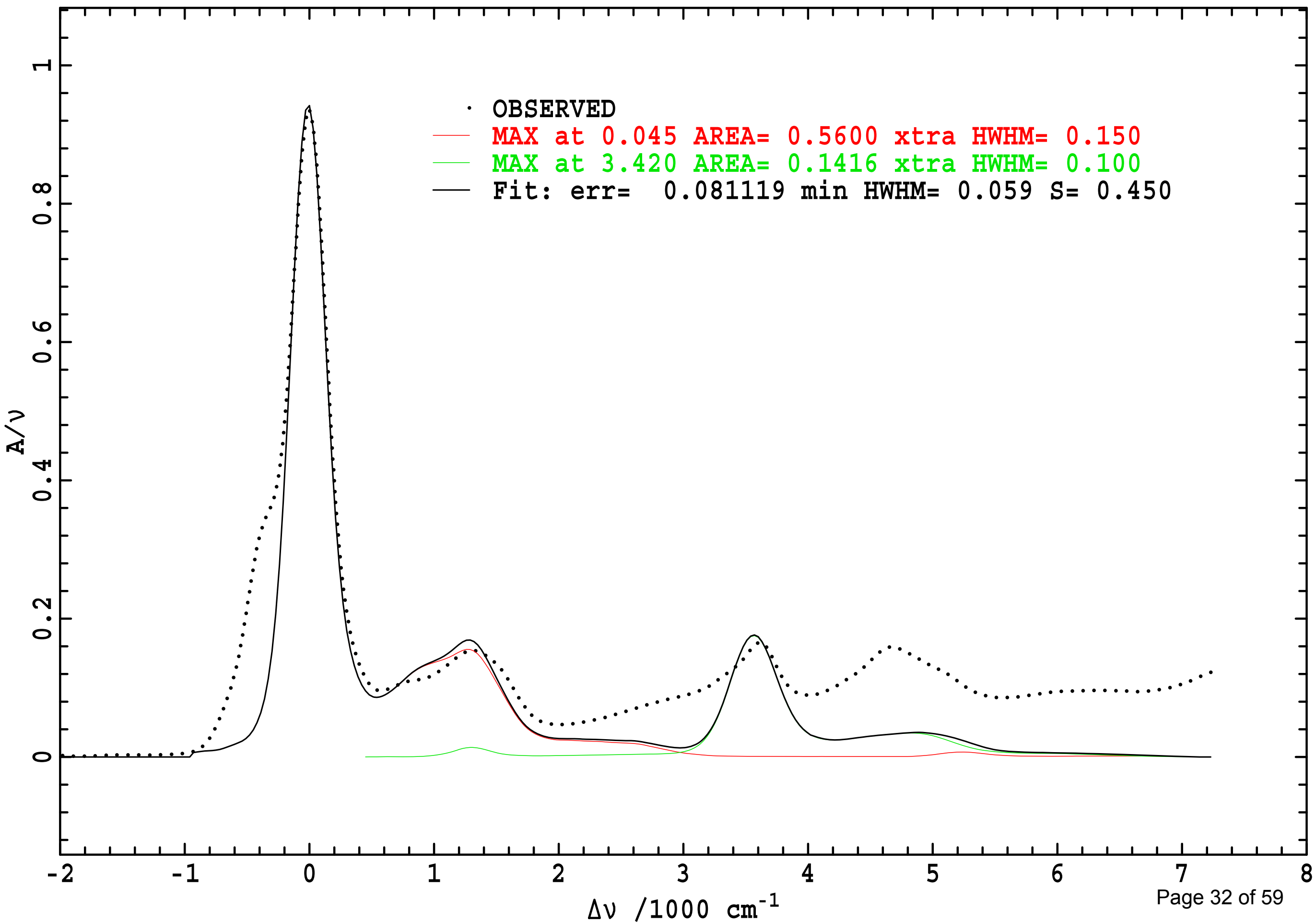

Fig. 1c: Pyromethylpheophorbide-a dioxane

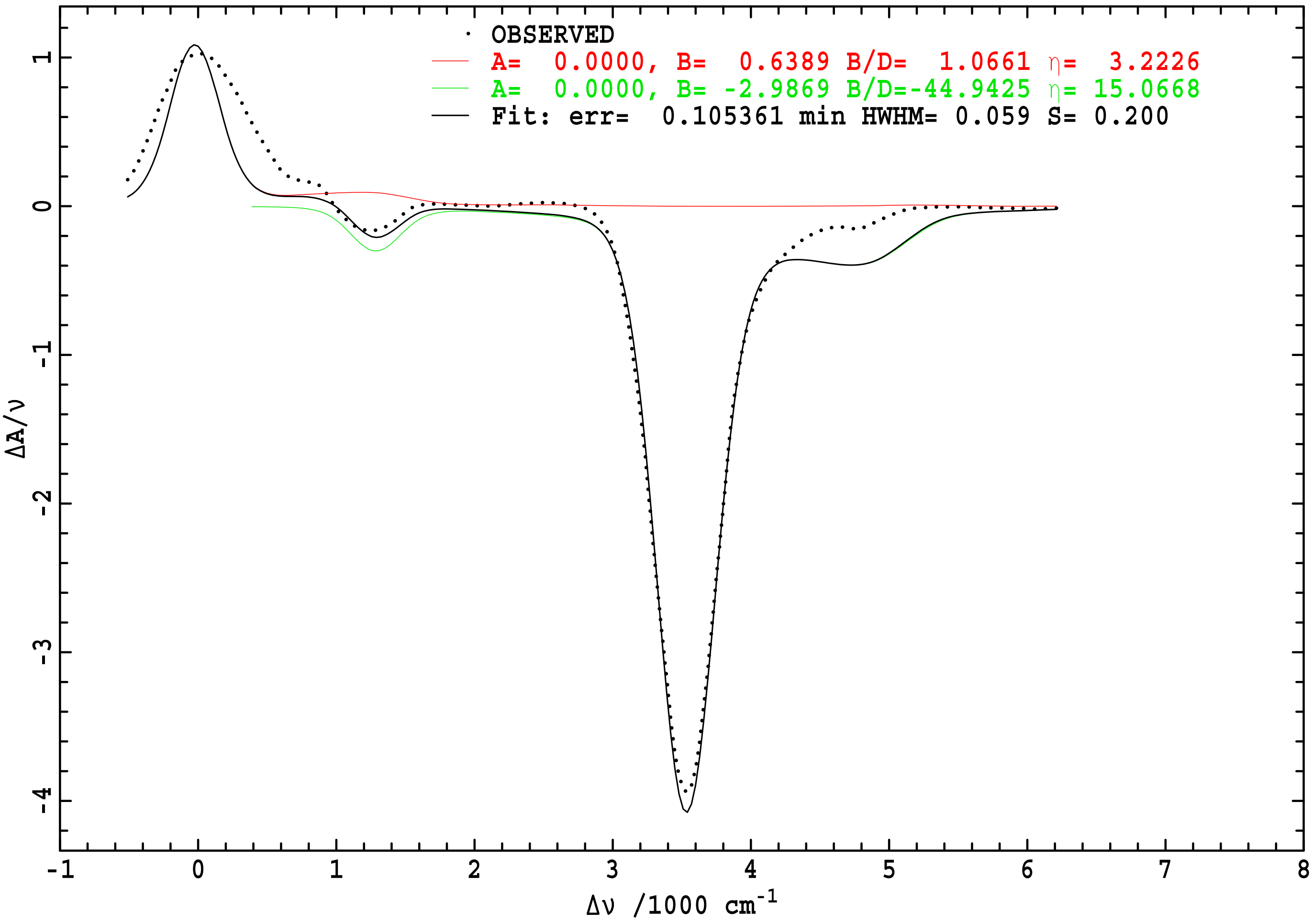


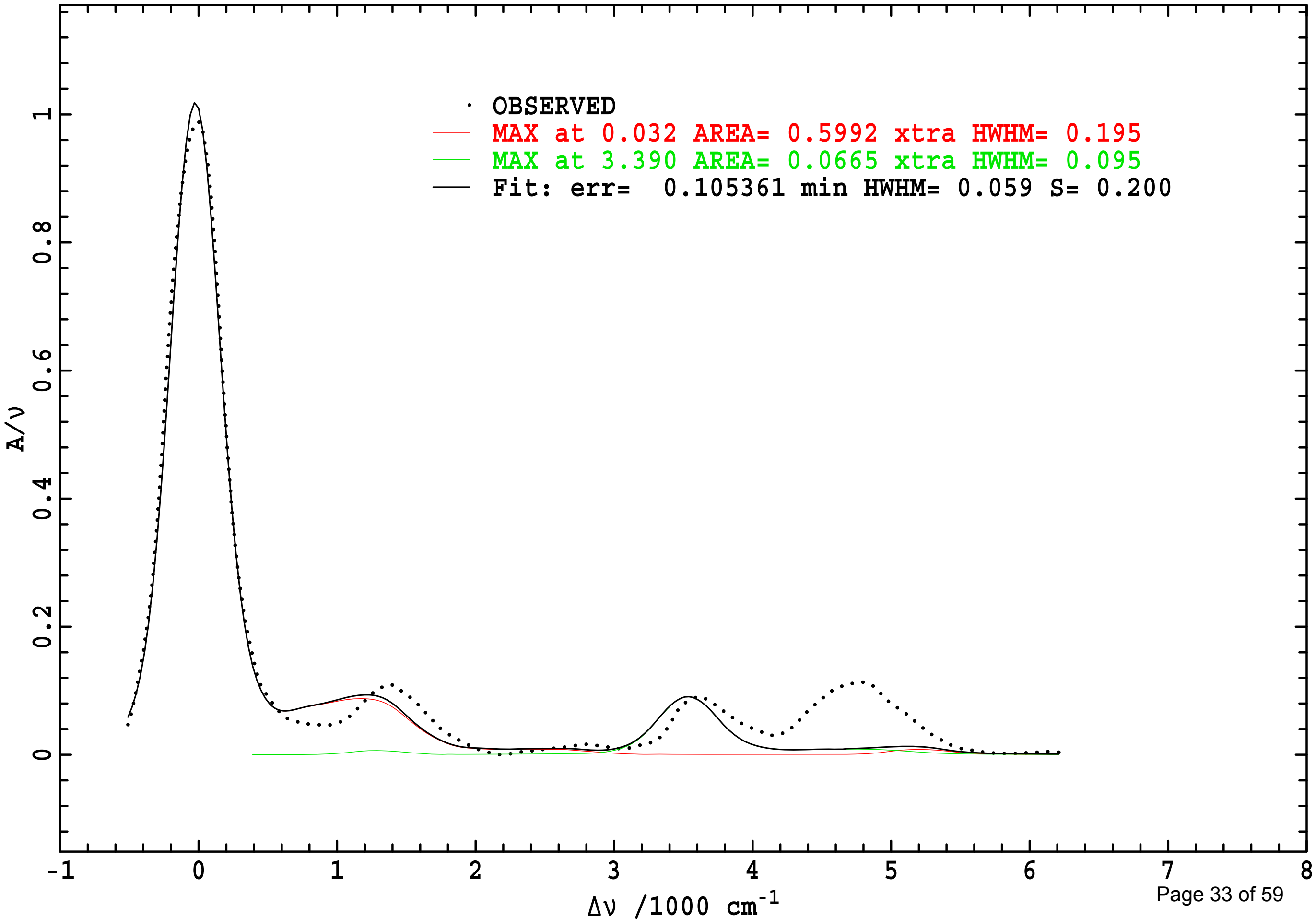

# Fig. 1d: BChl-a pyridine

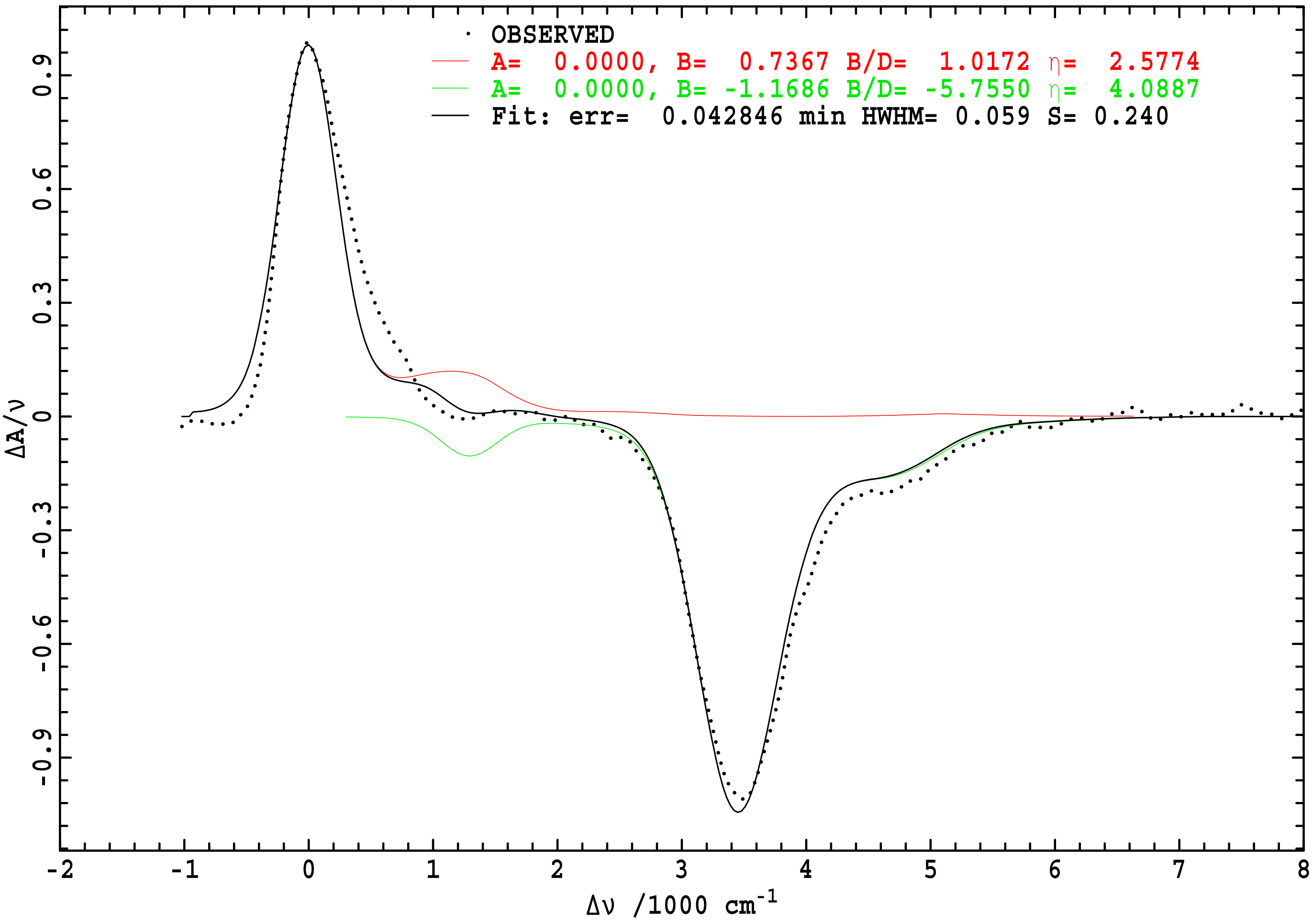


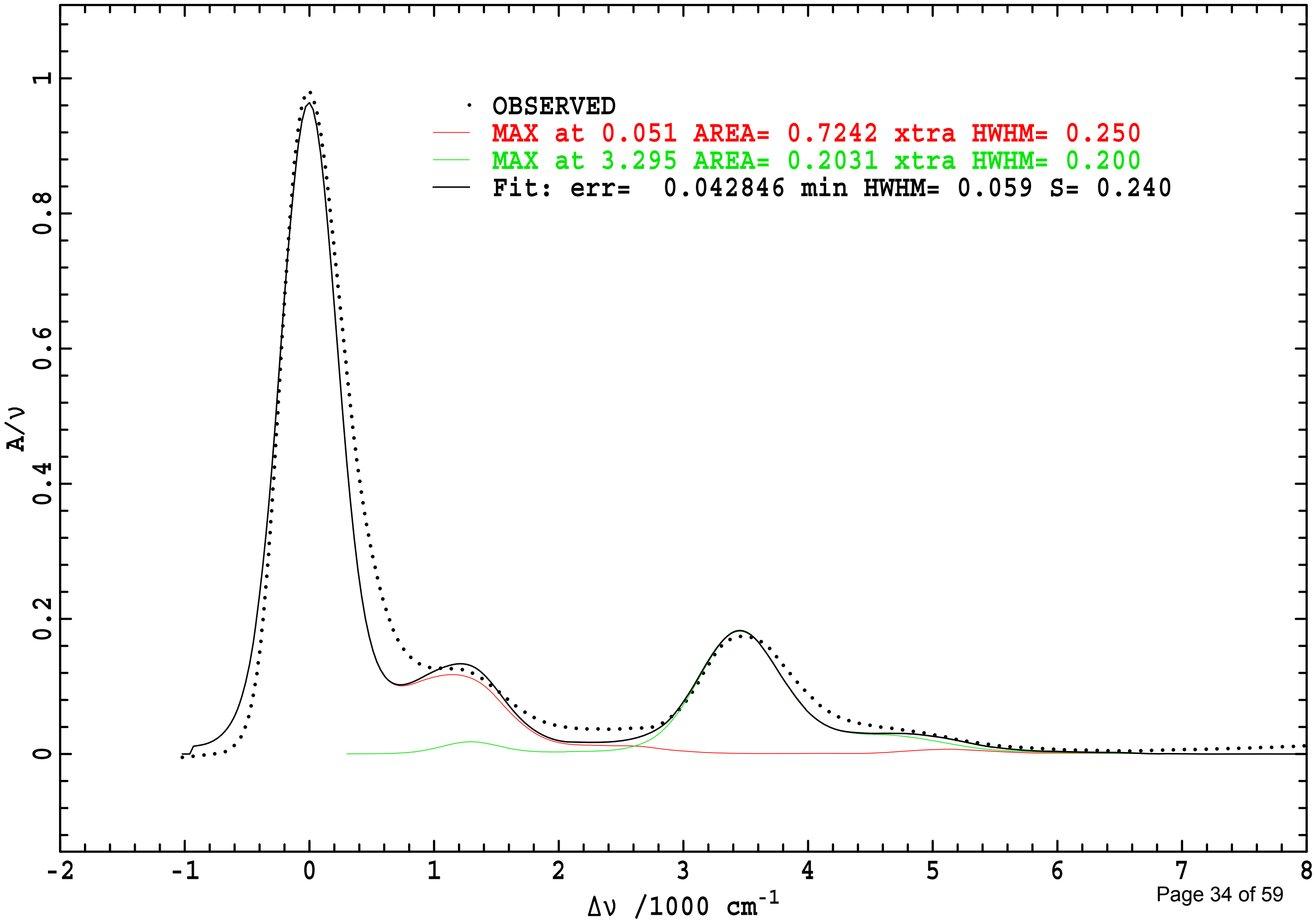

# Fig. 1e: Zn(II)-Chl-a ether

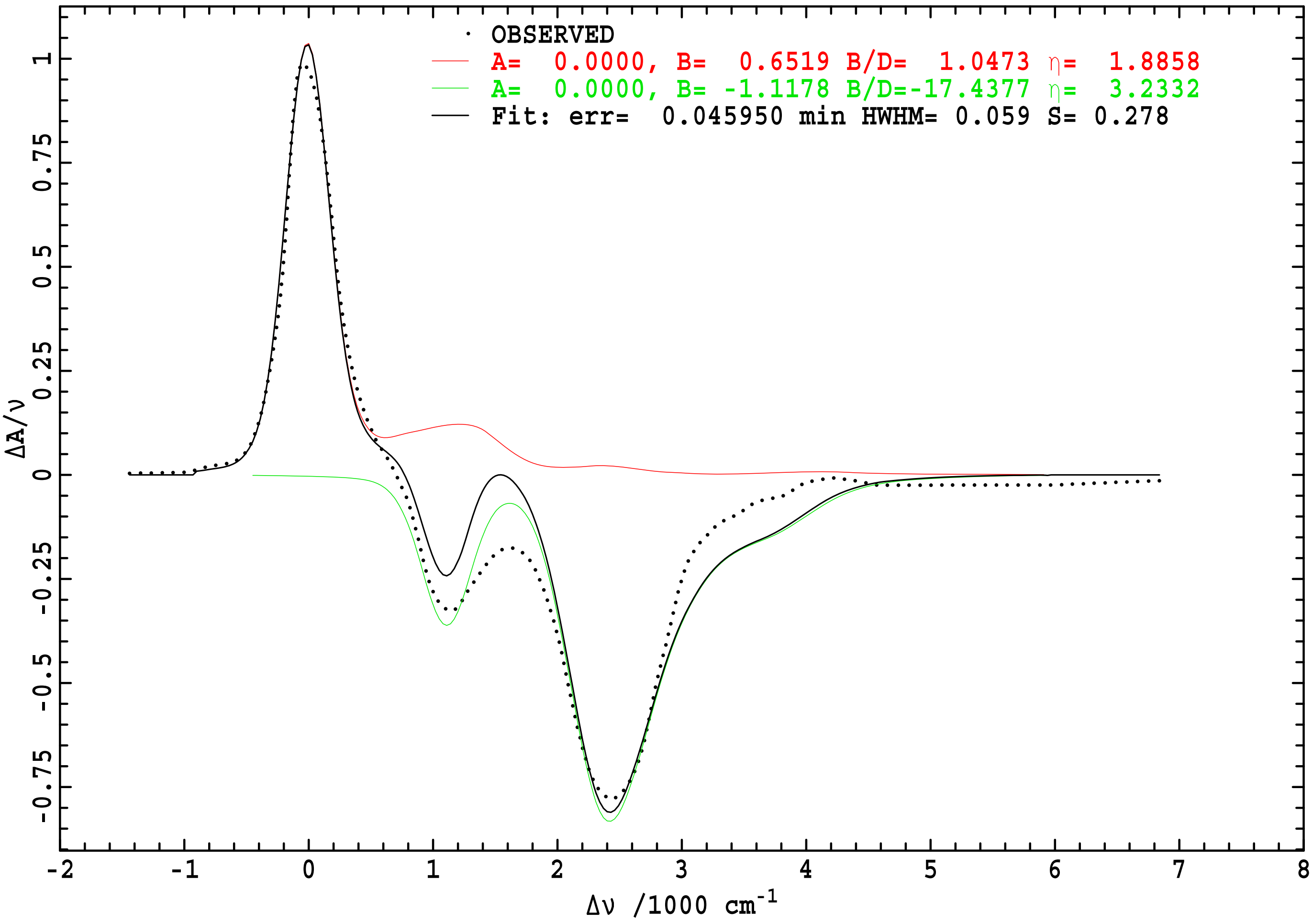


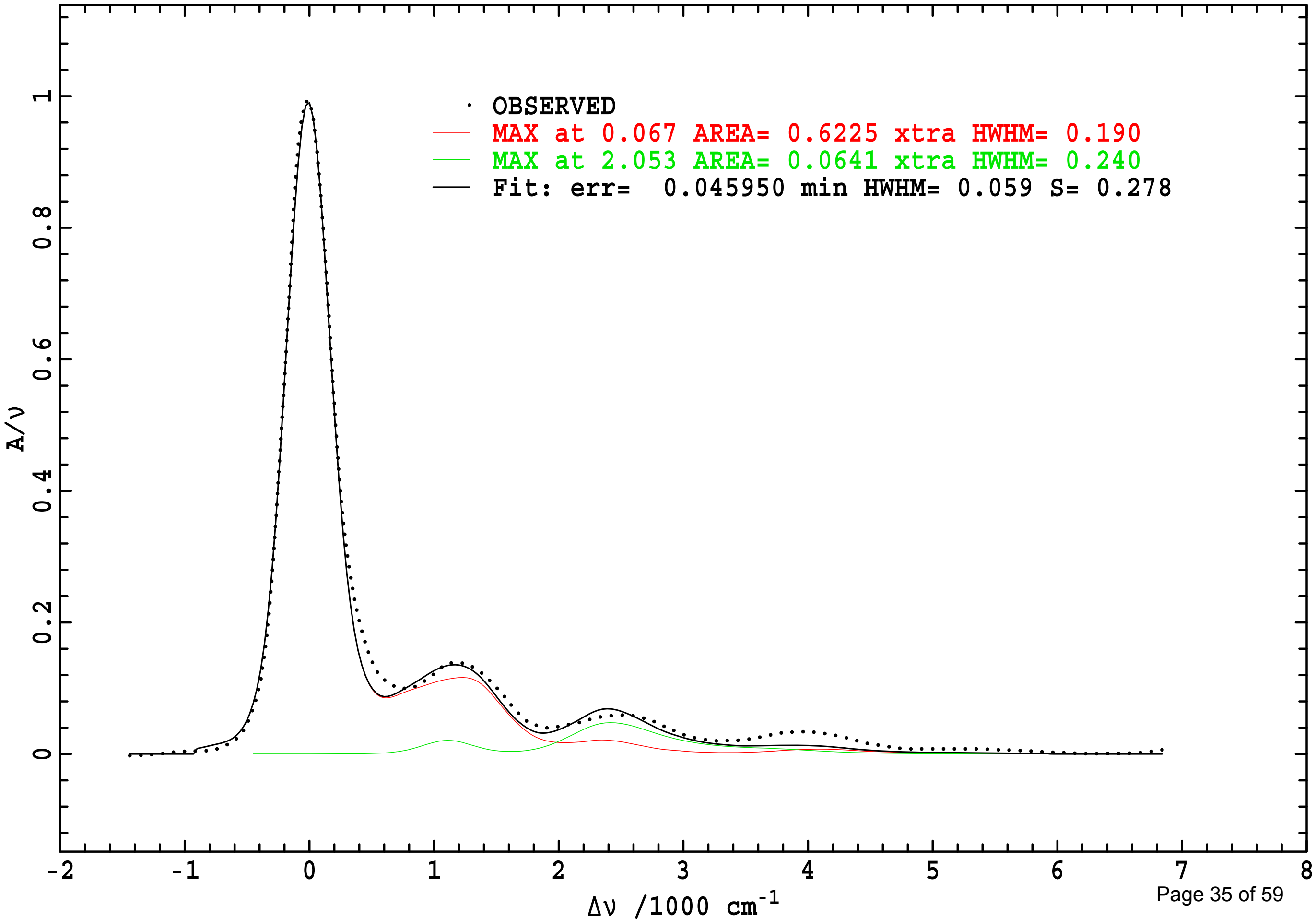

# Fig. 1f: Chl-a ether

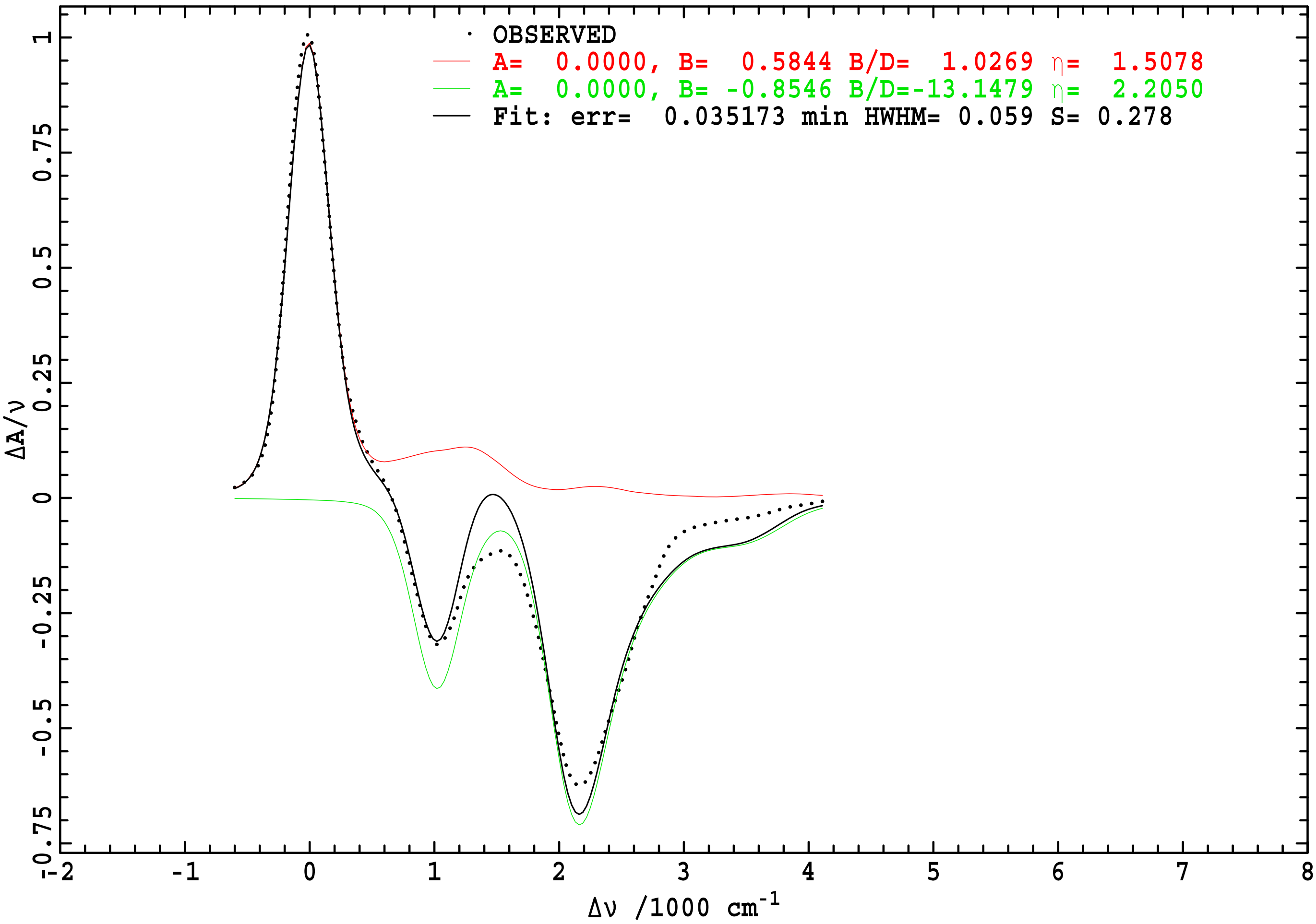


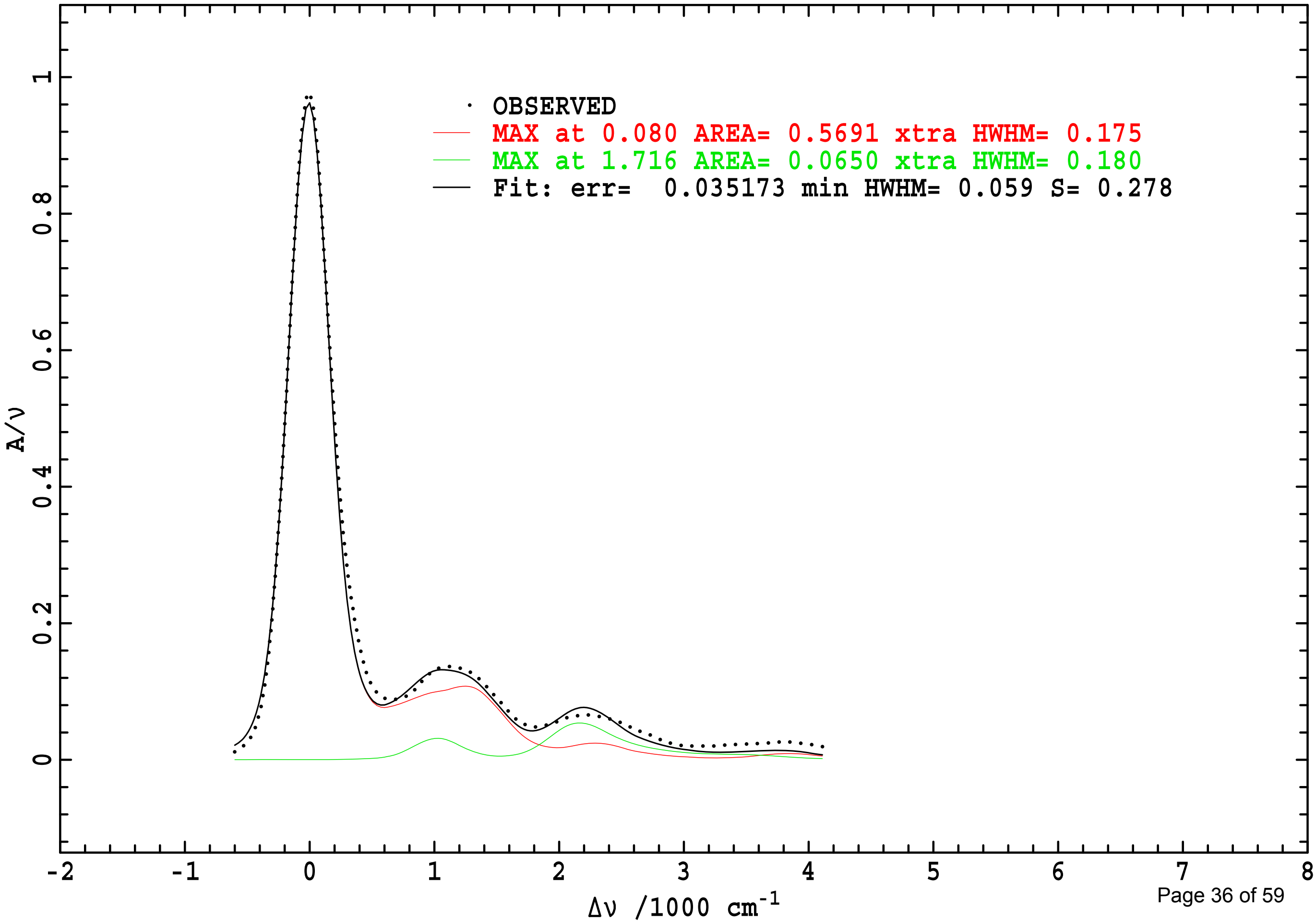

# Fig. 1g: BChl-d ether

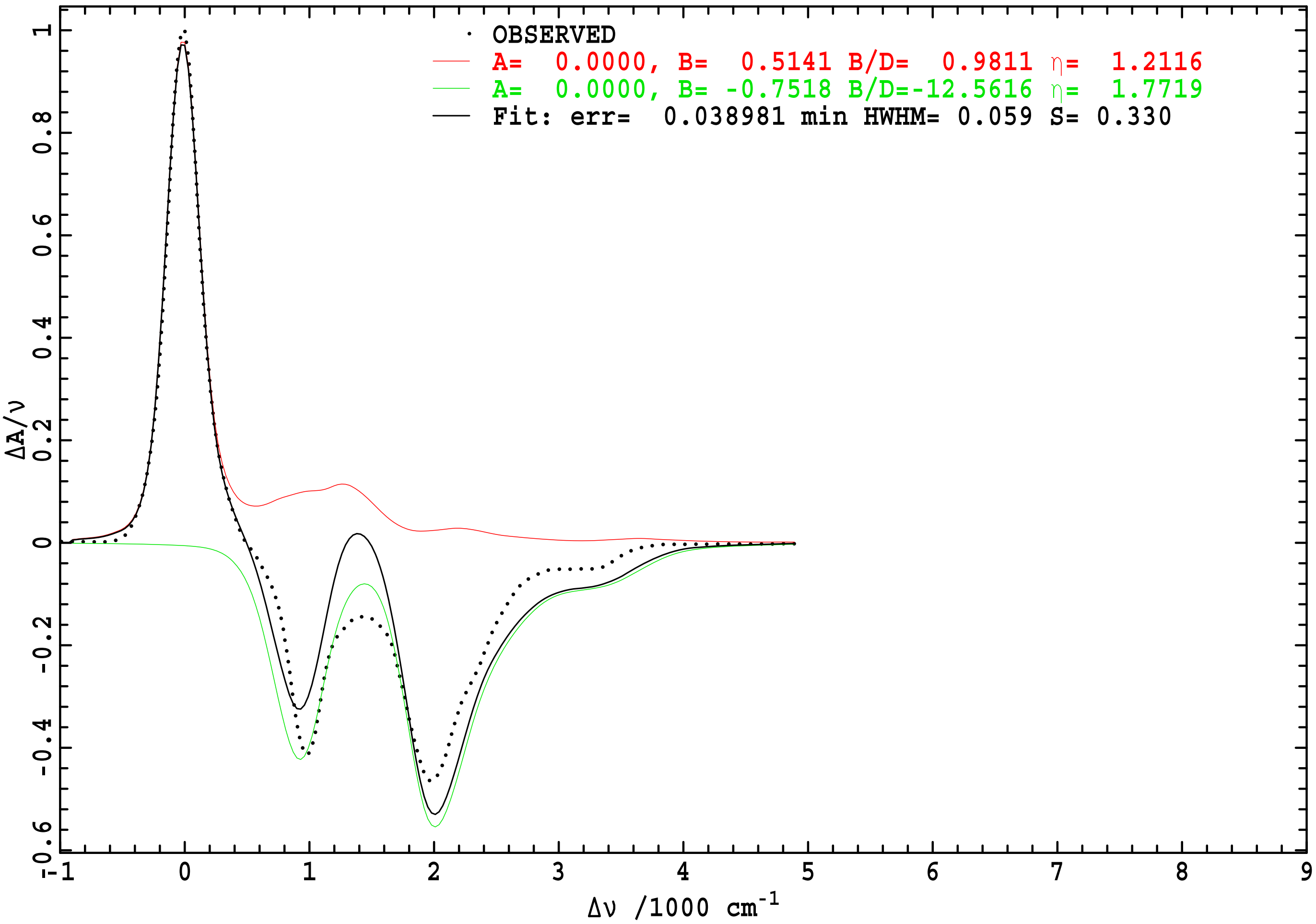


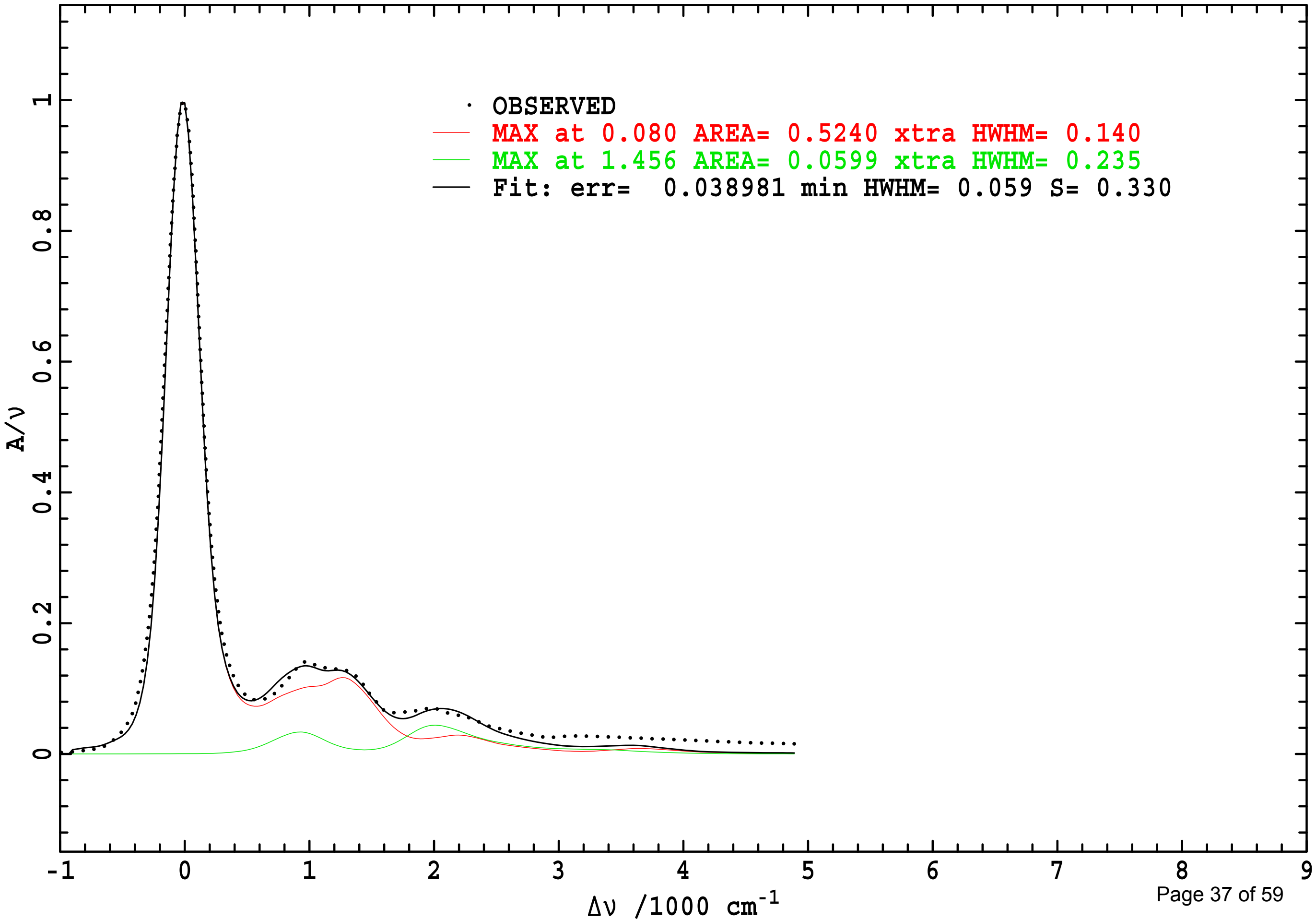

Fig. 1h: BChl-c ether

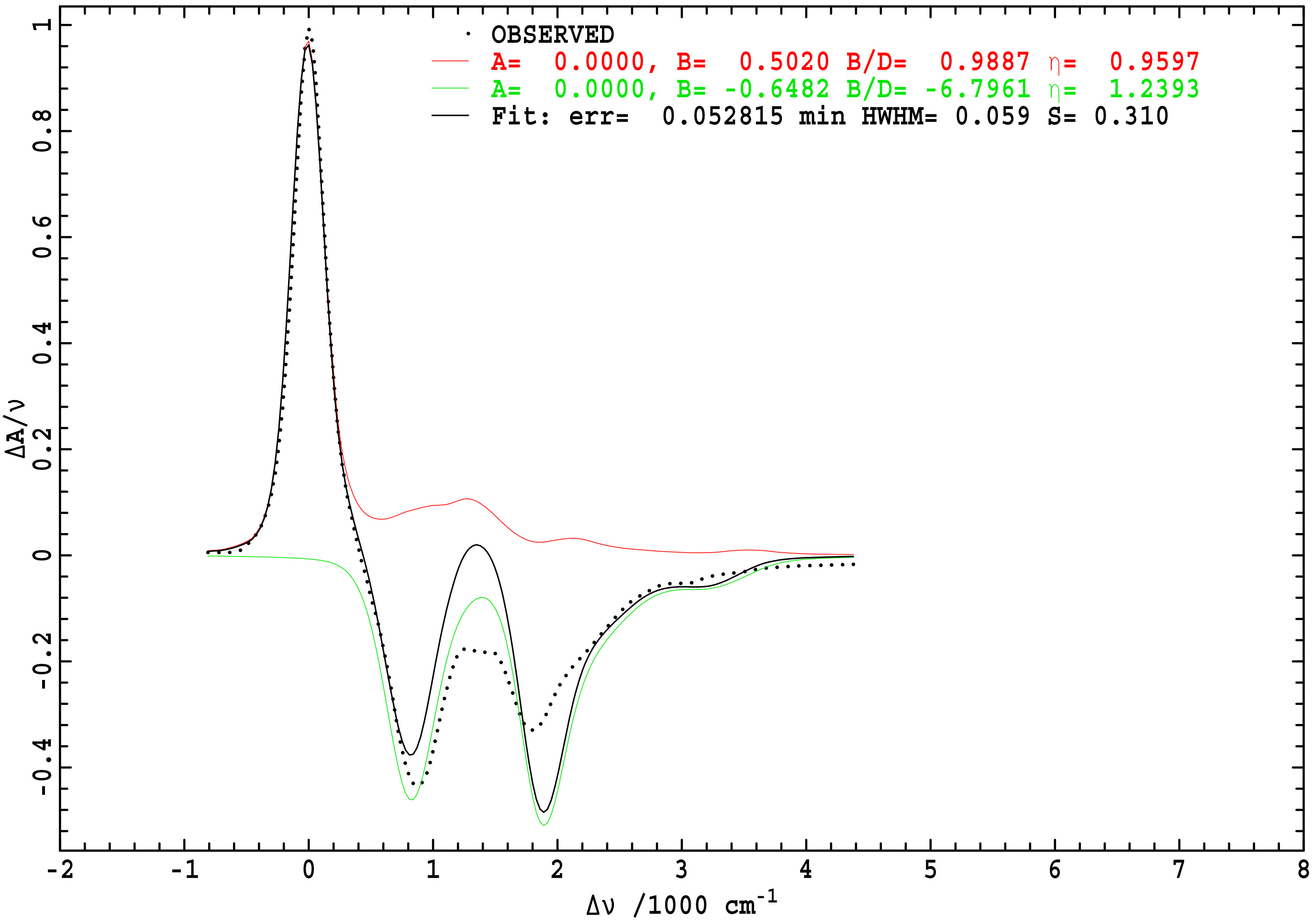


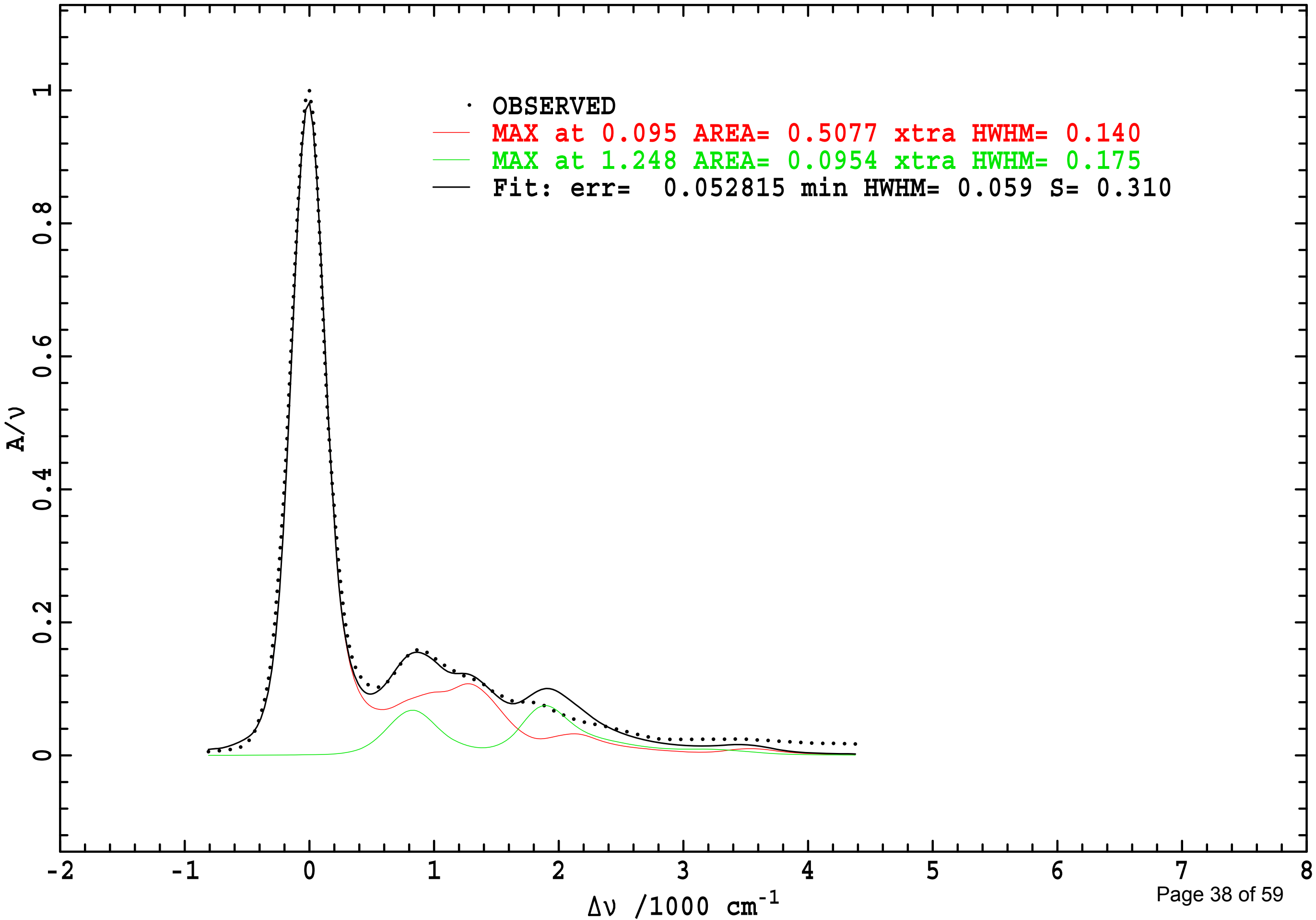

Fig. 1i: ChlZ(D1) PS-II 1.7 K

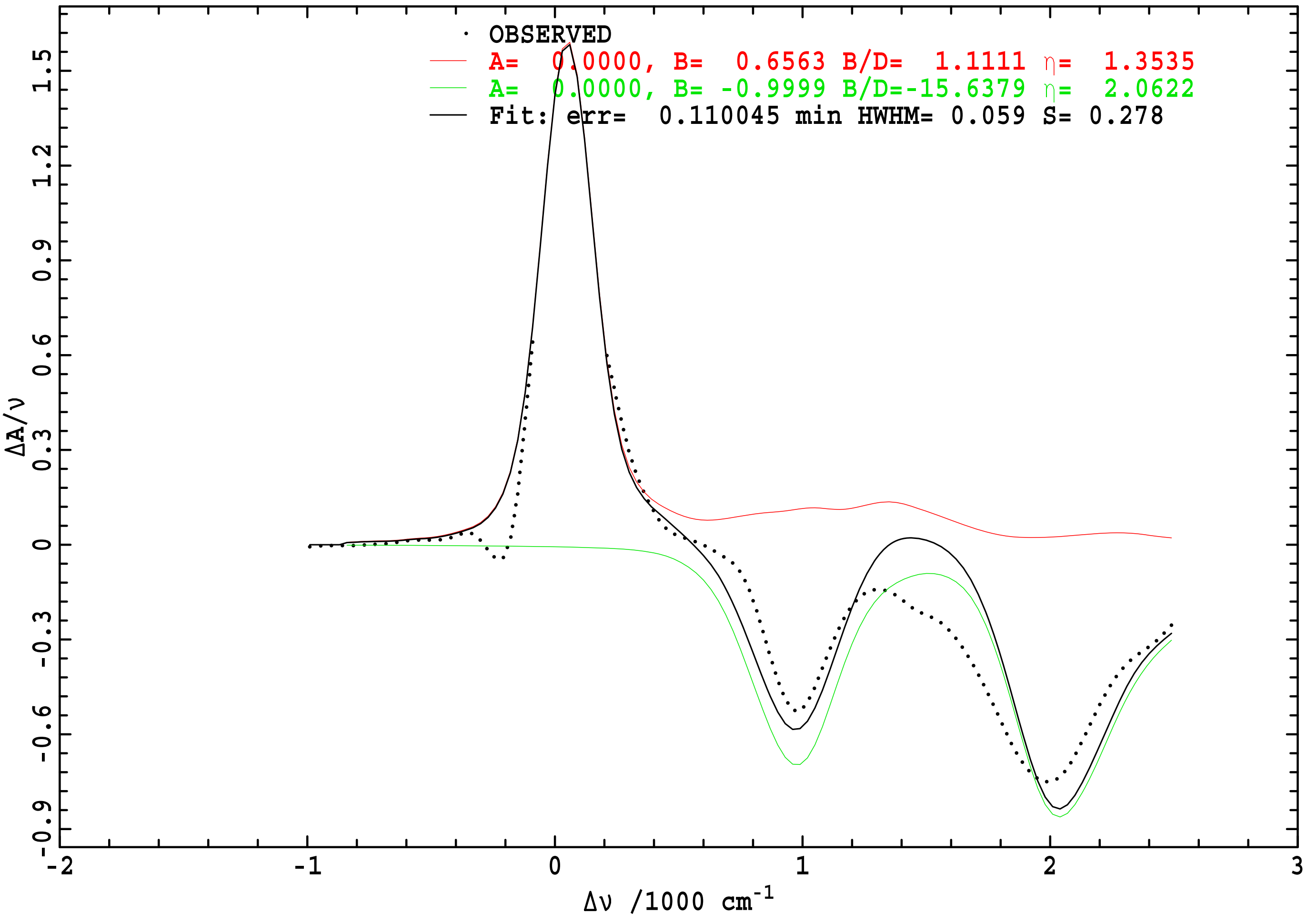


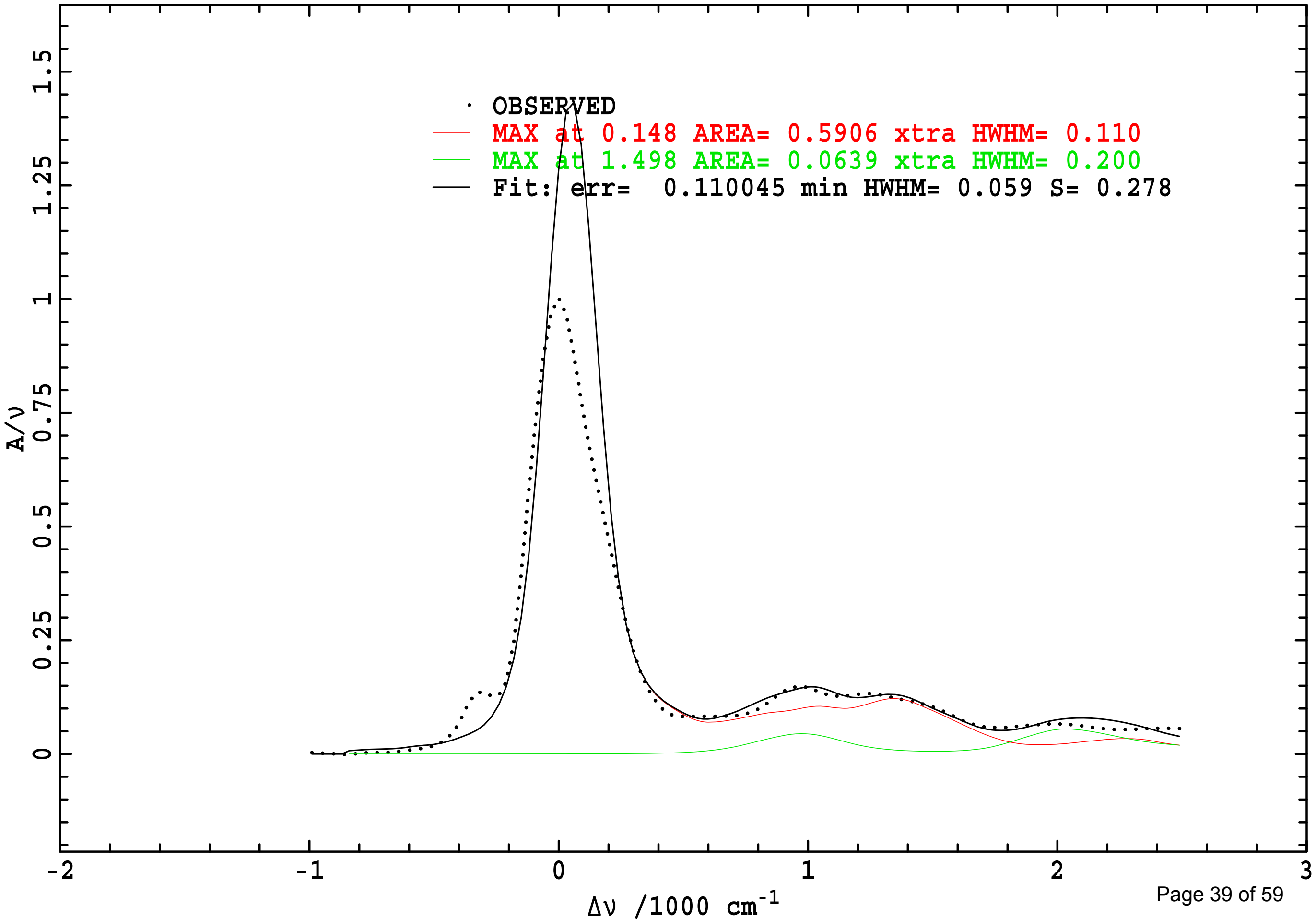

Fig. 1j: Chl-a pyridine

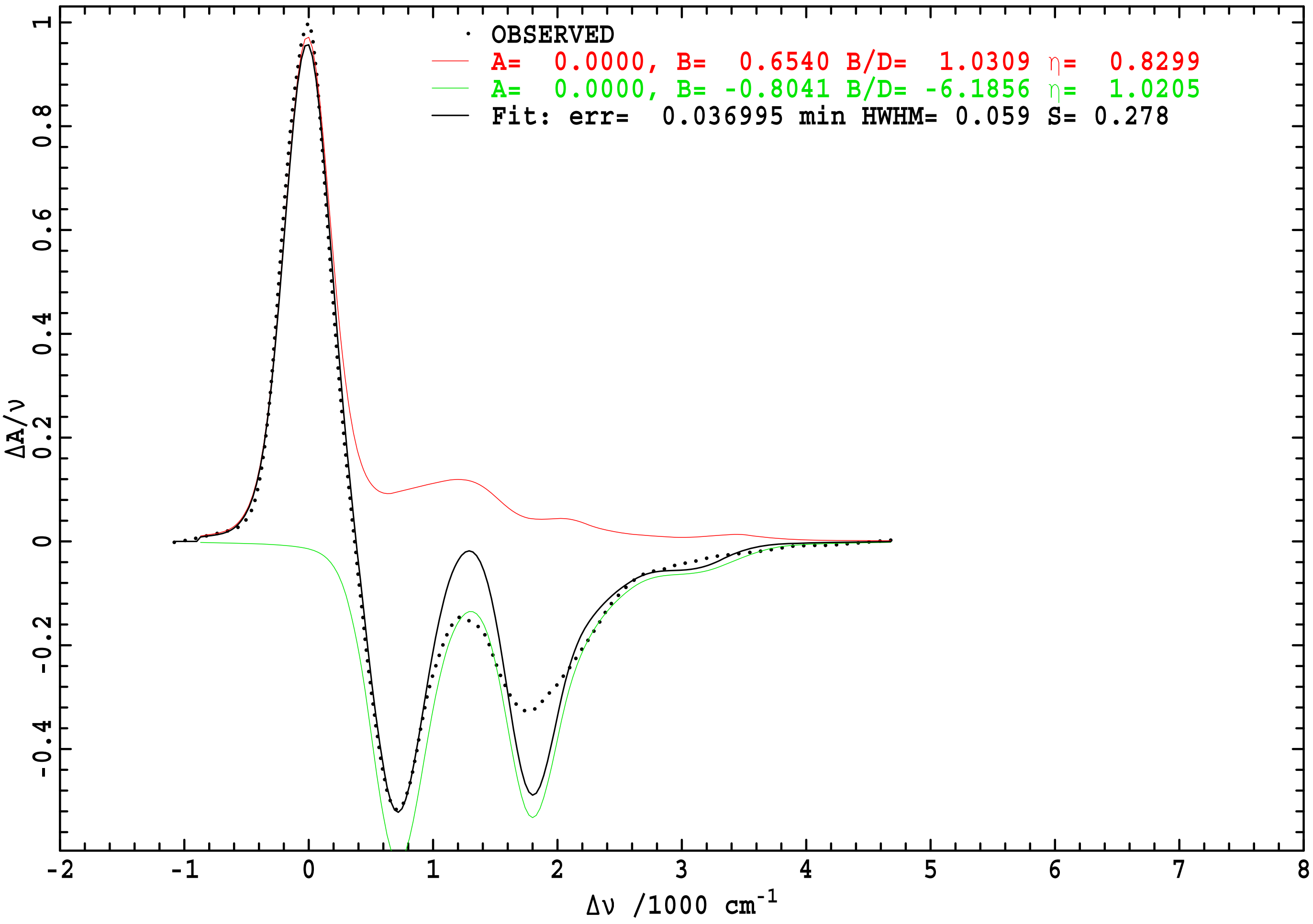


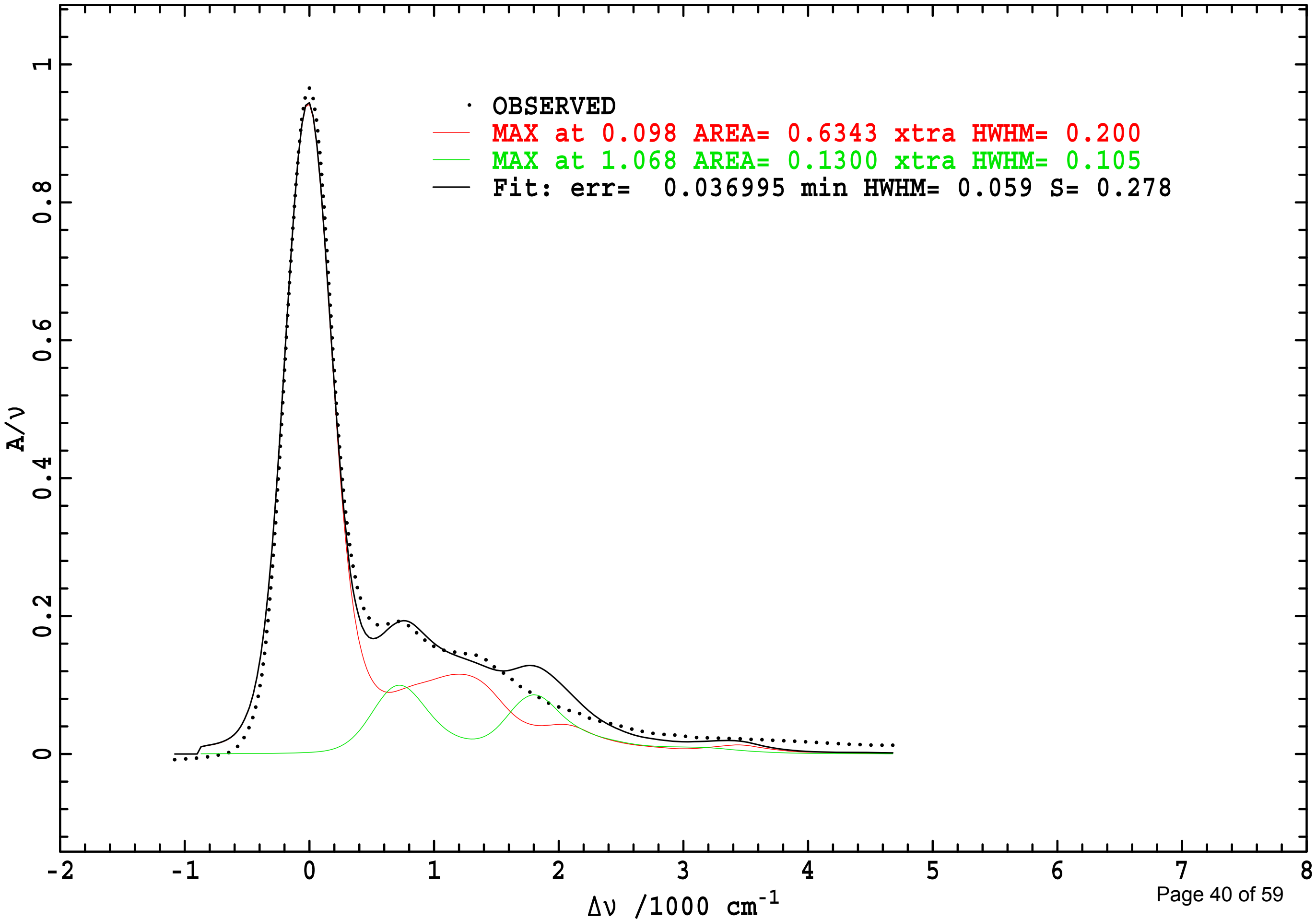

## Fig. 1k: BChl-d pyridine

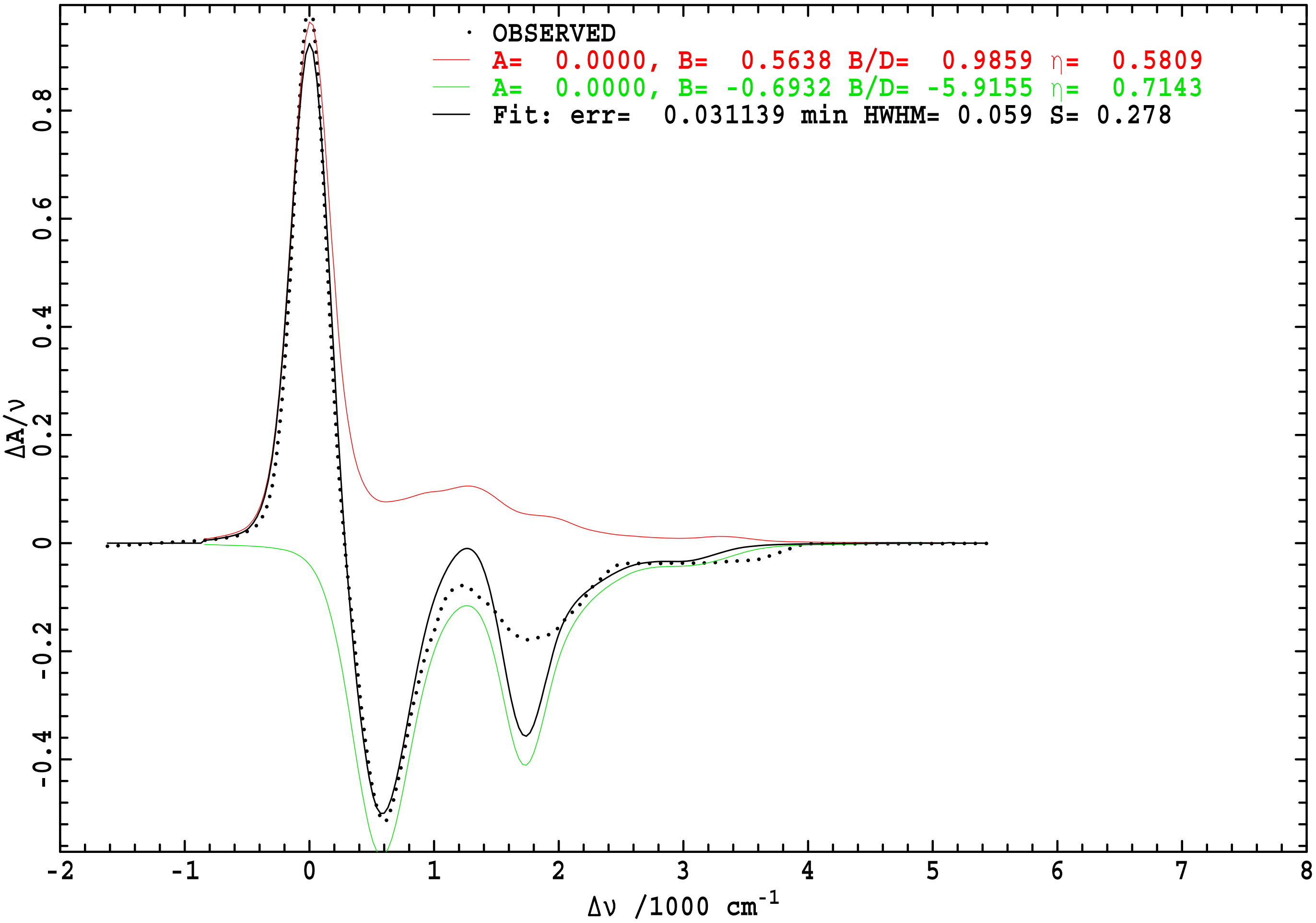


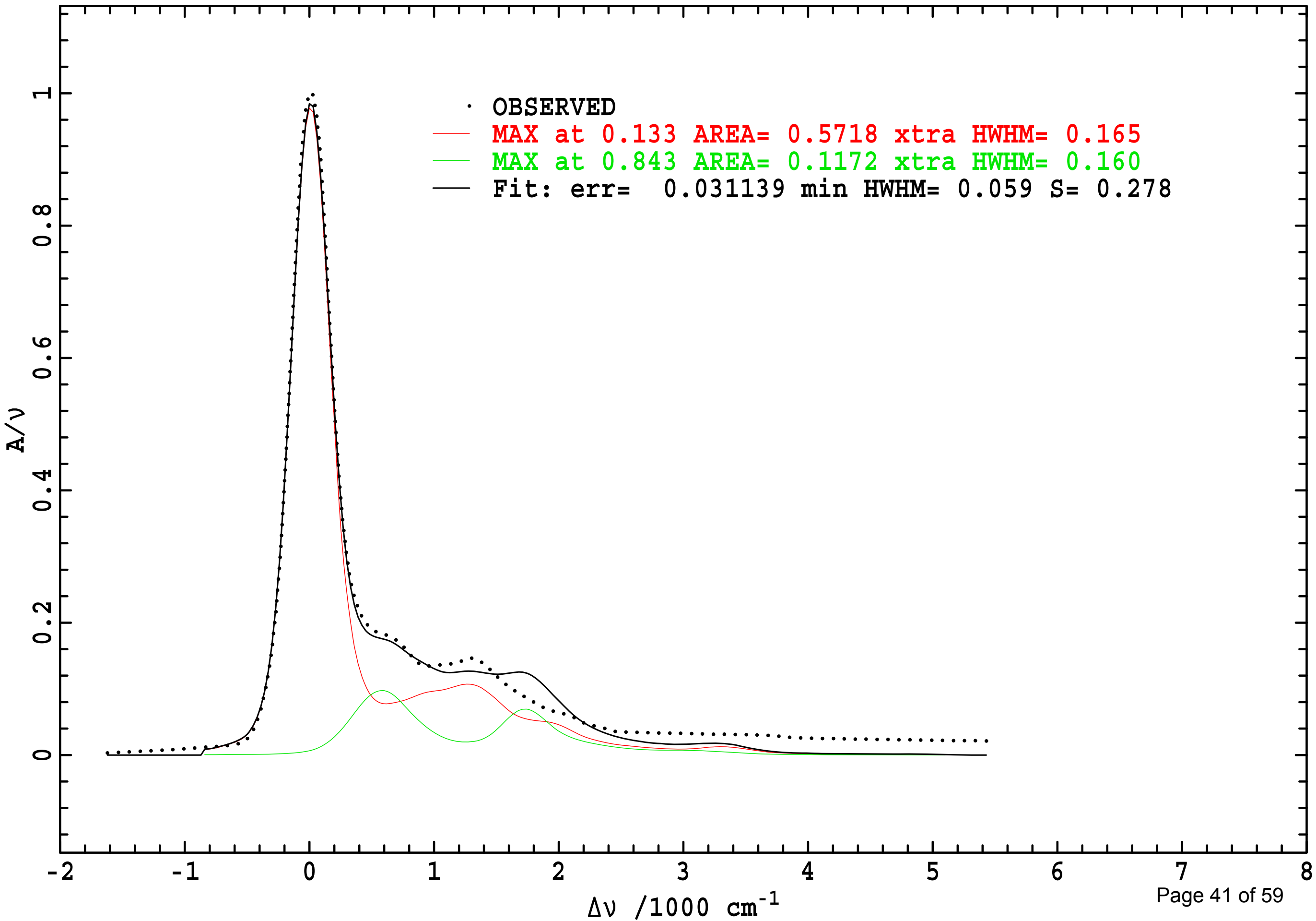

Fig. 1l: BChl-c pyridine

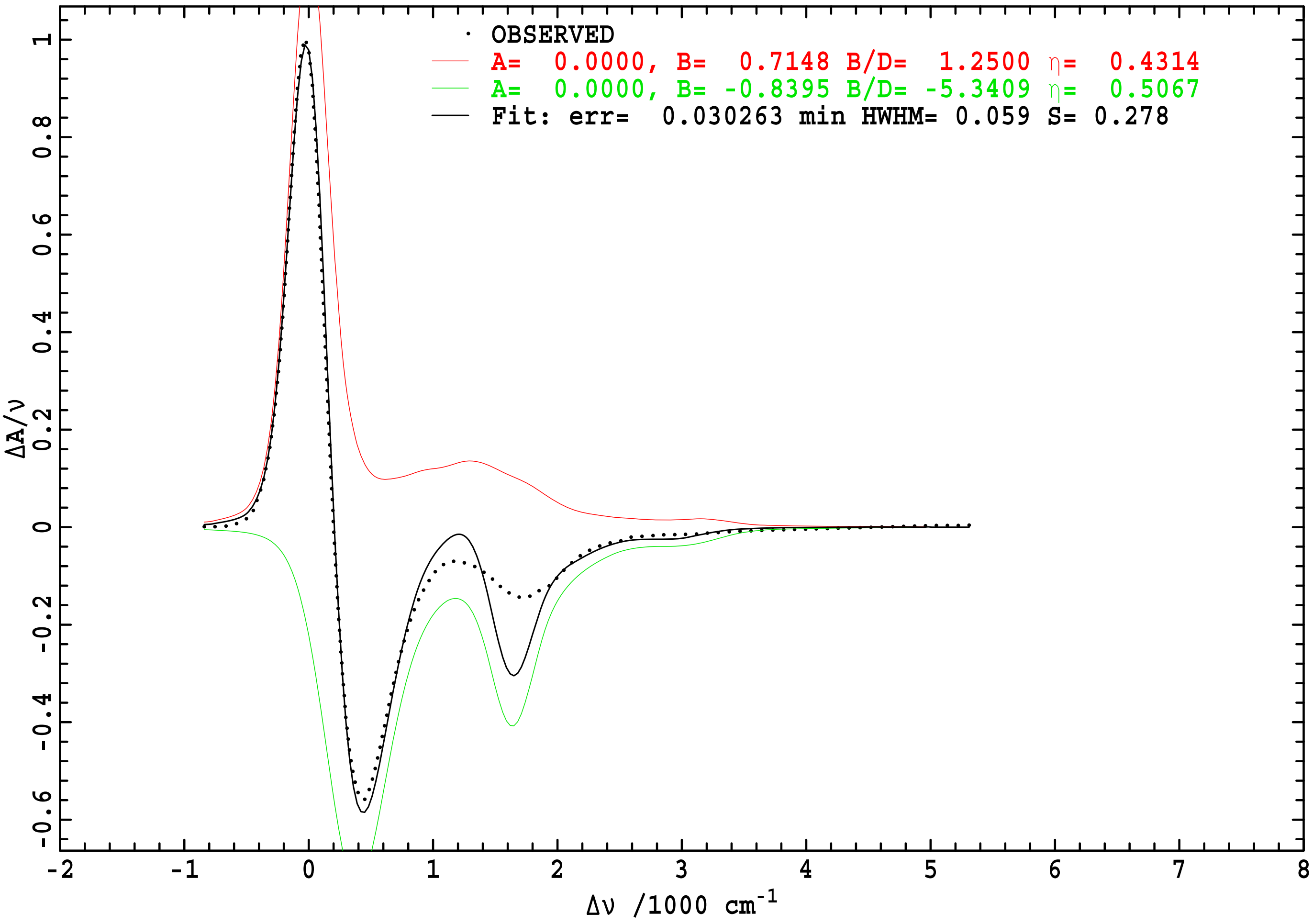


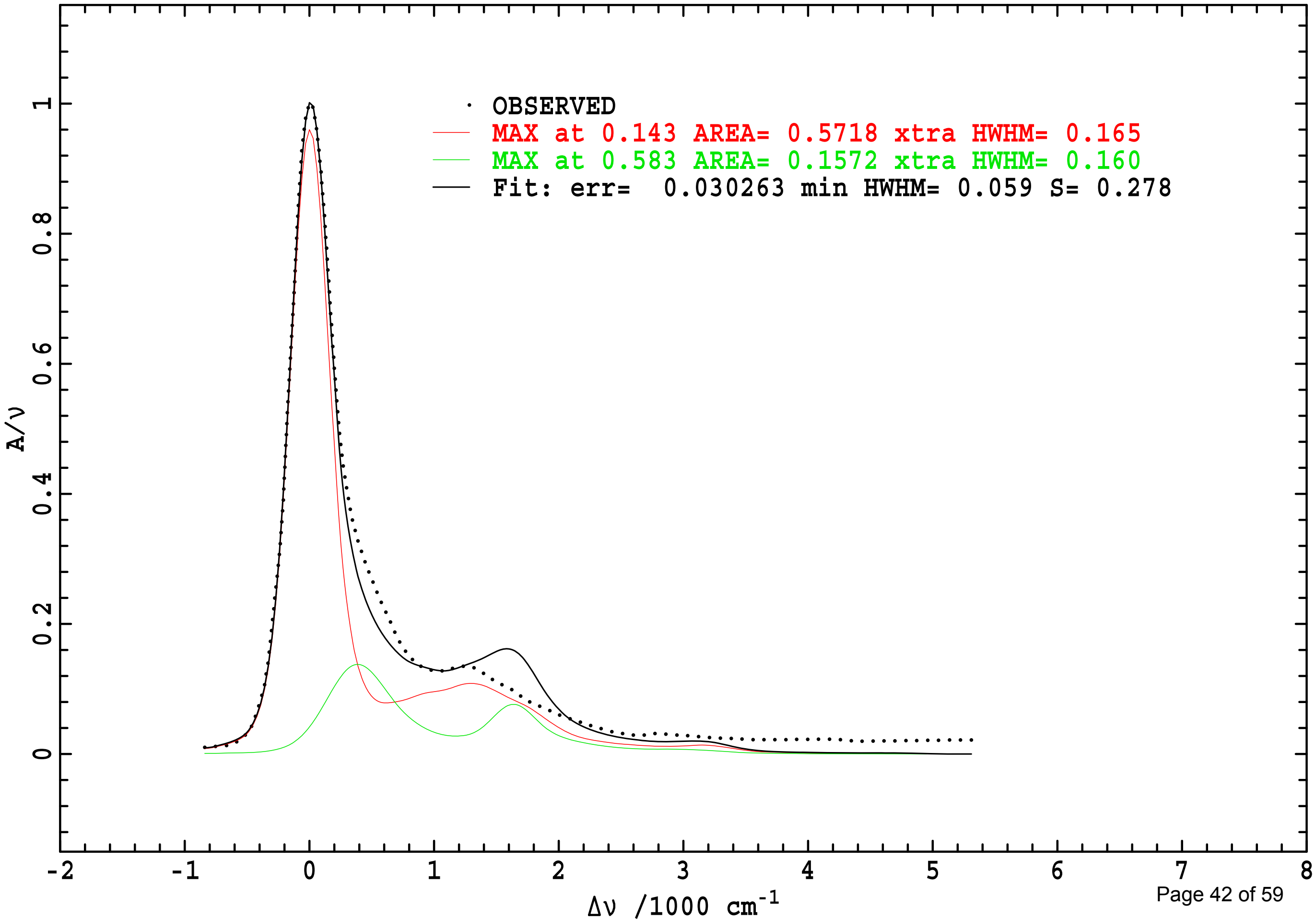

## Fig. S1a: Rhodochlorin DME dioxane

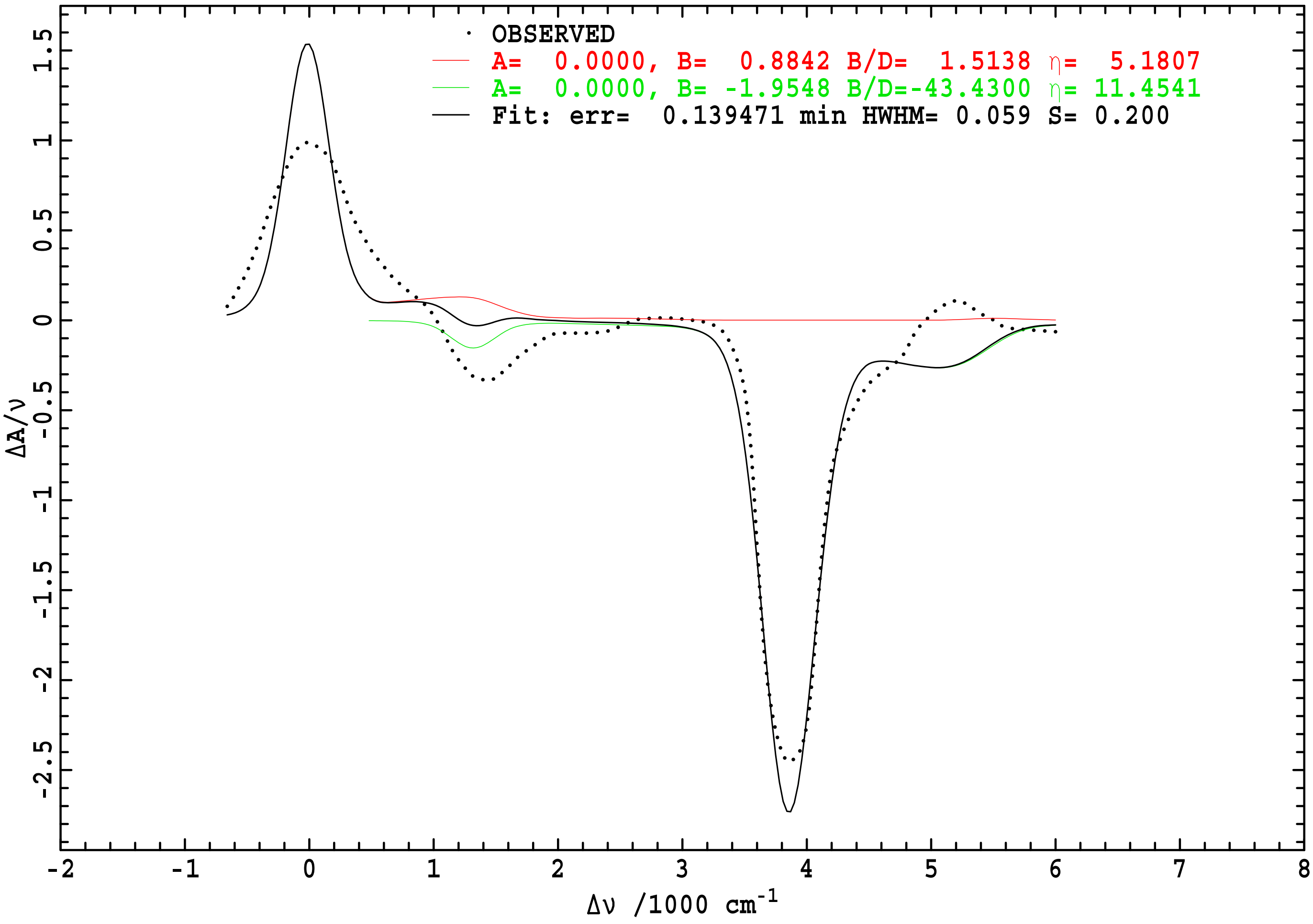


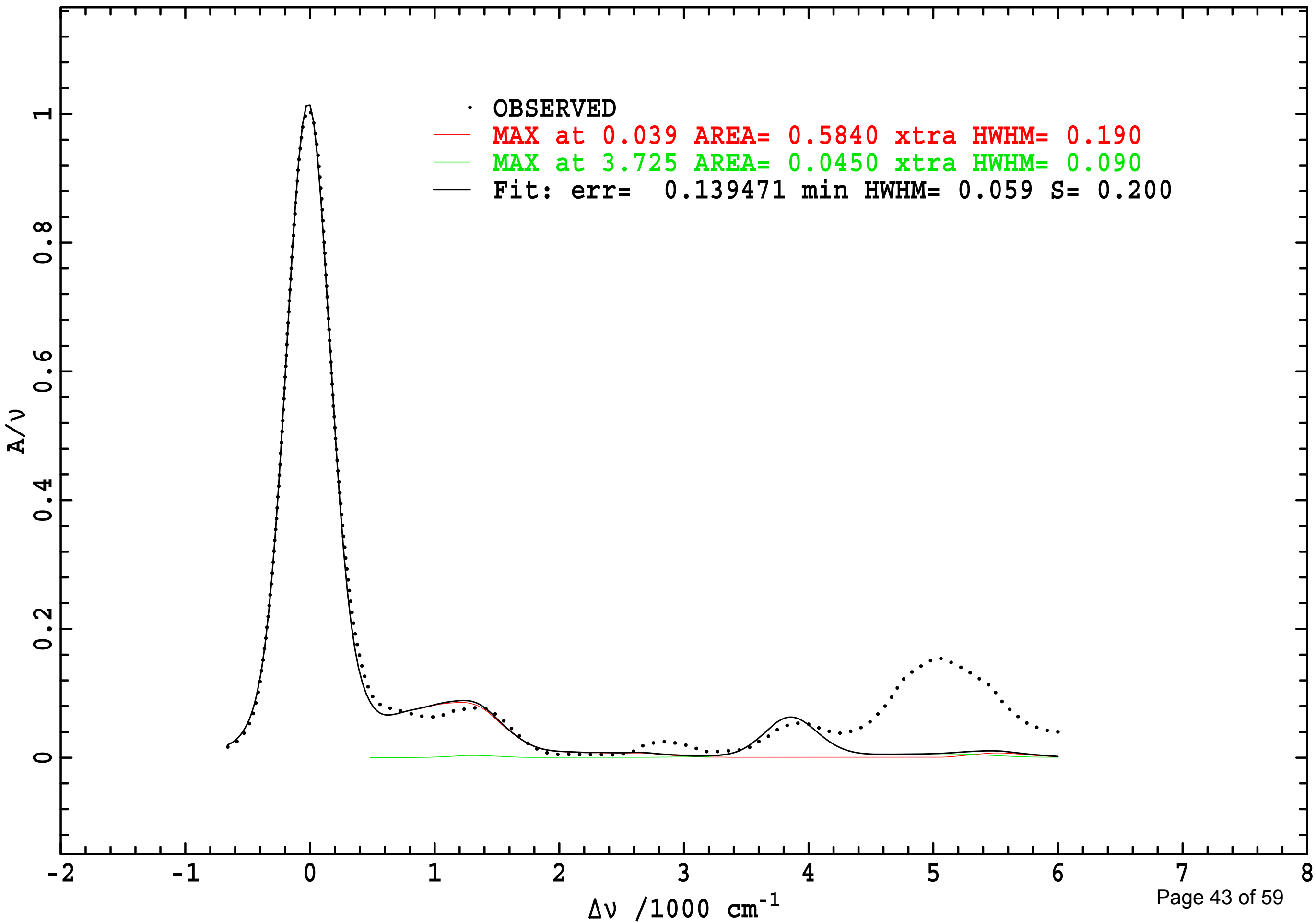

## Fig. S1b: Isochlorin-e5 DME dioxane

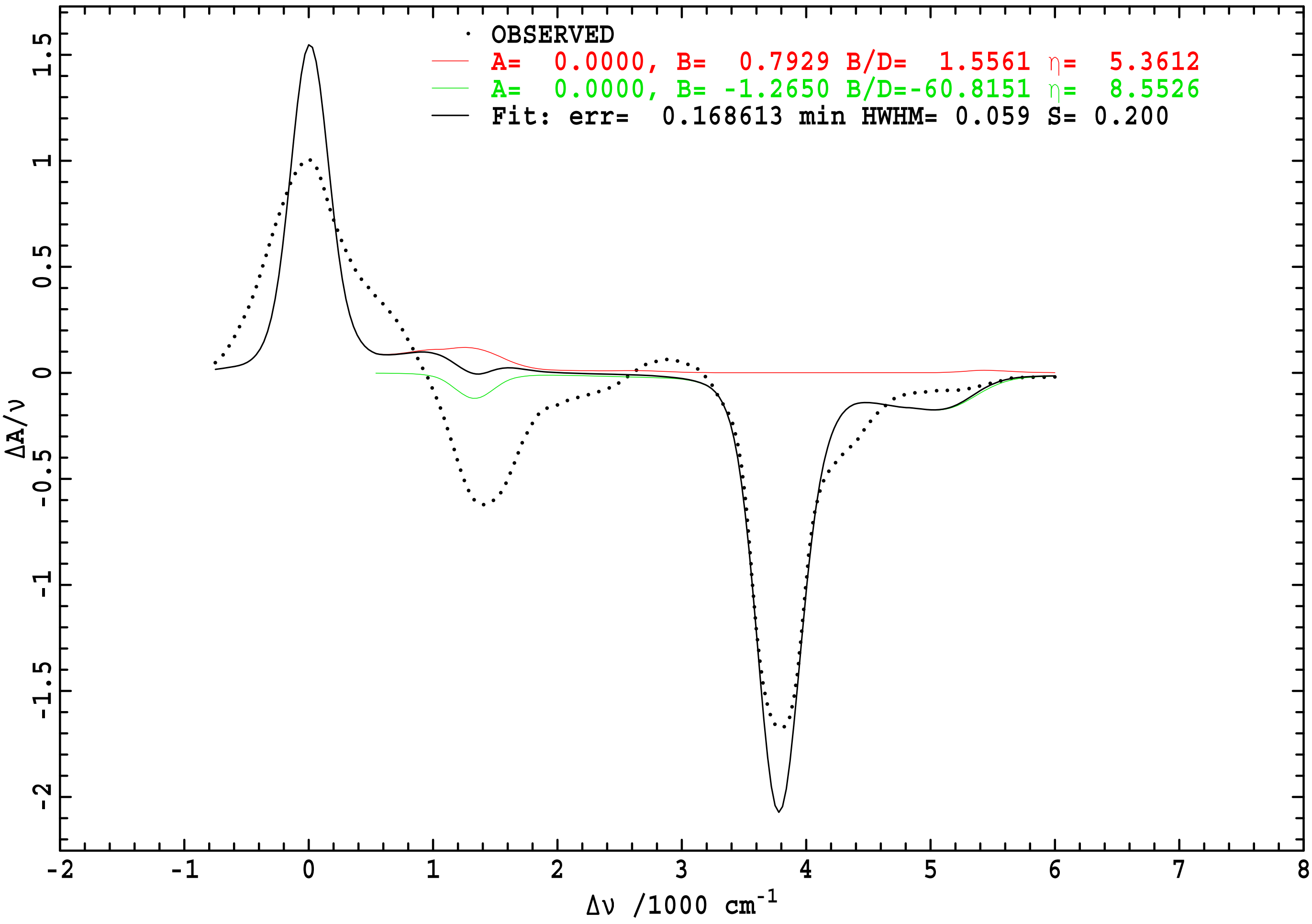


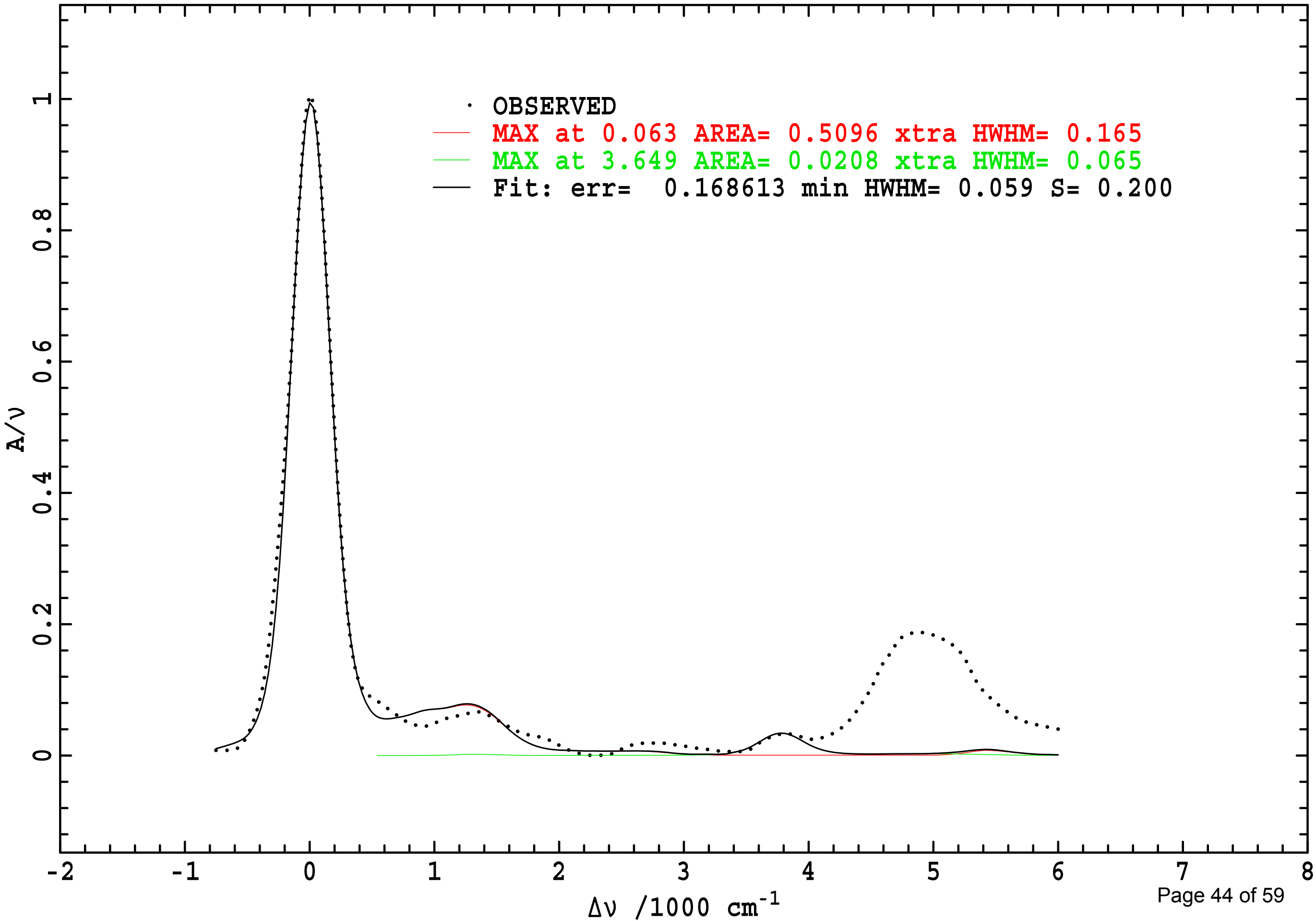

Fig. S1c: Chlorin-e6 TME dioxane

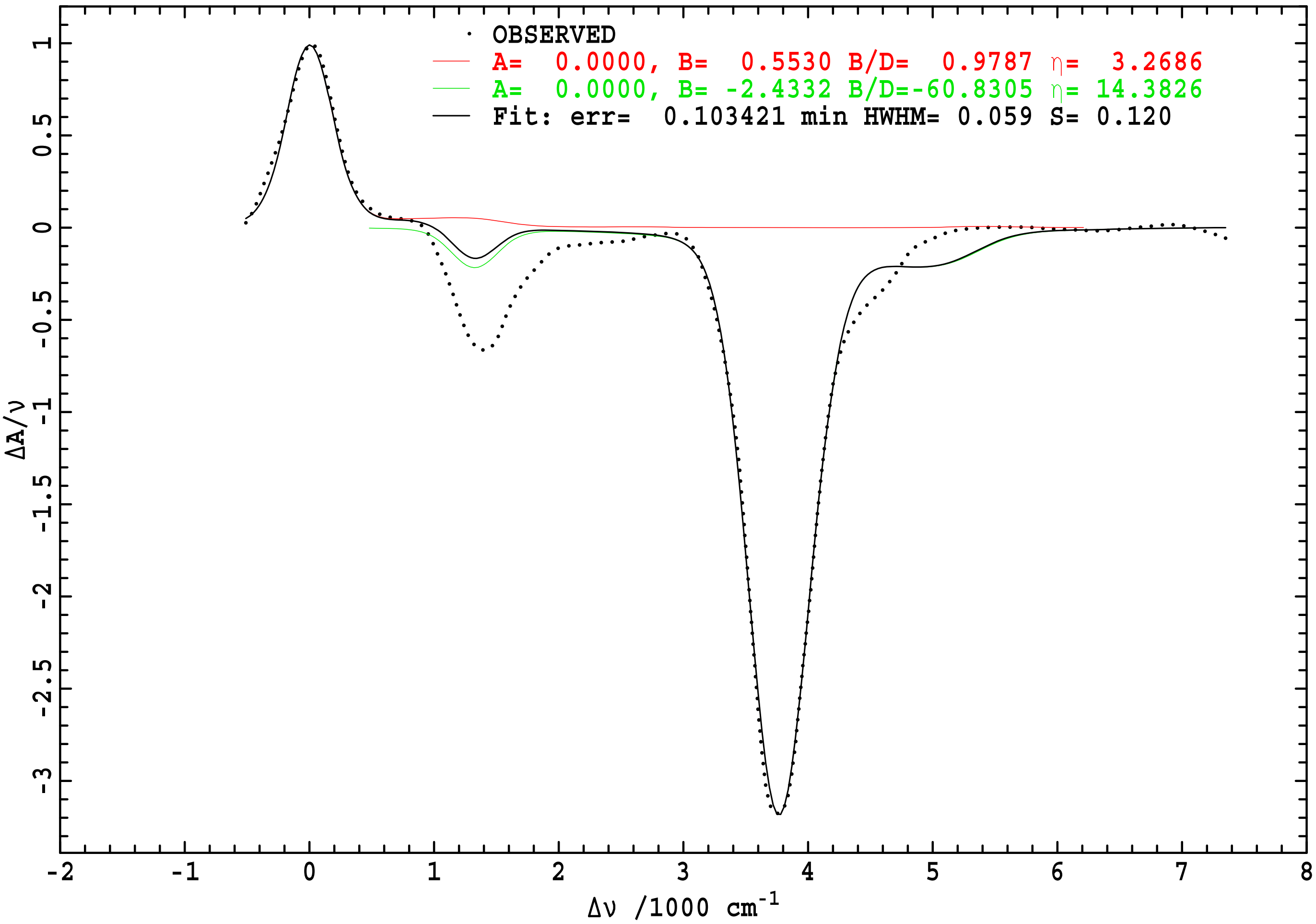


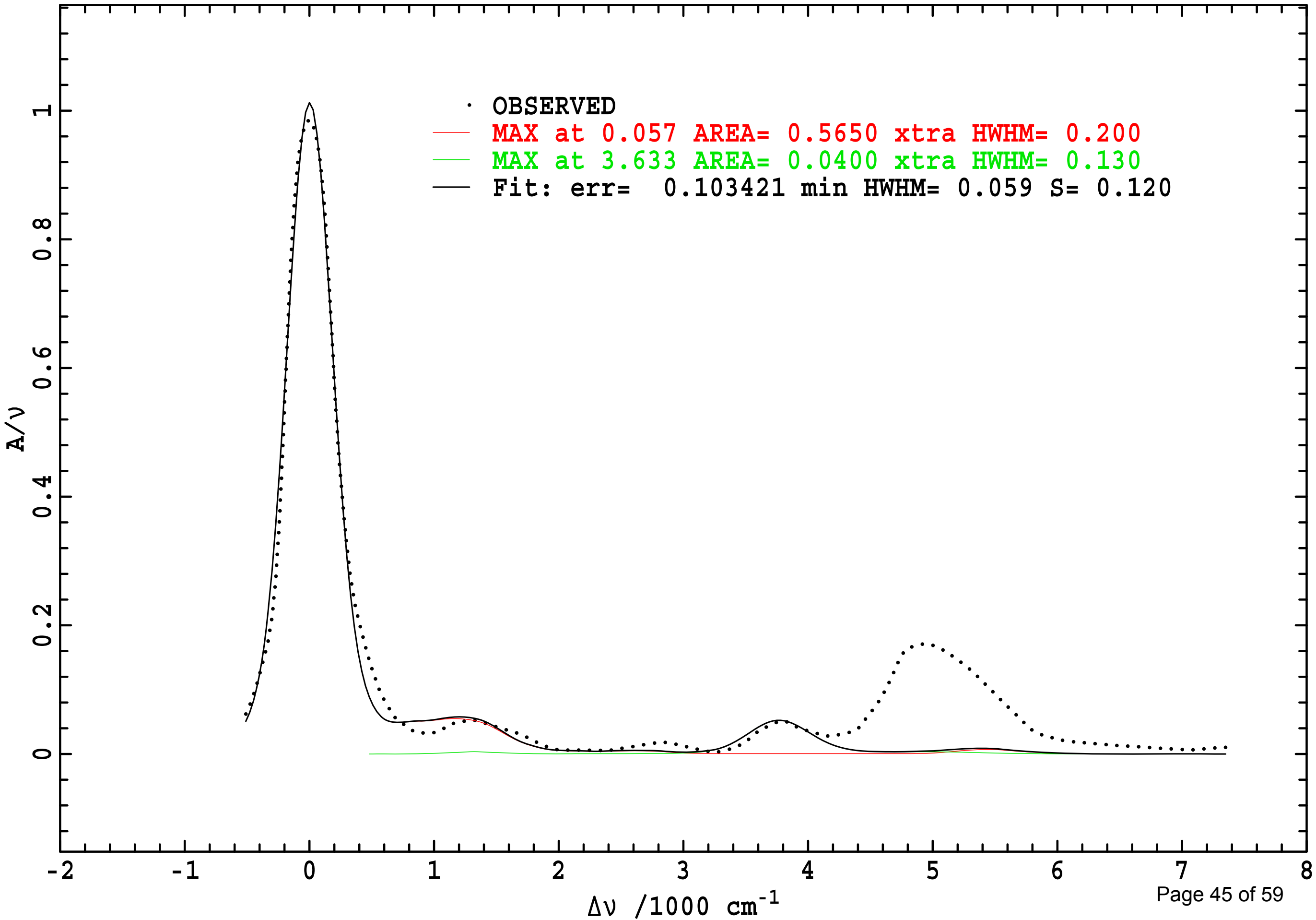

Fig. S1d: Methylpheophorbide-a dioxane

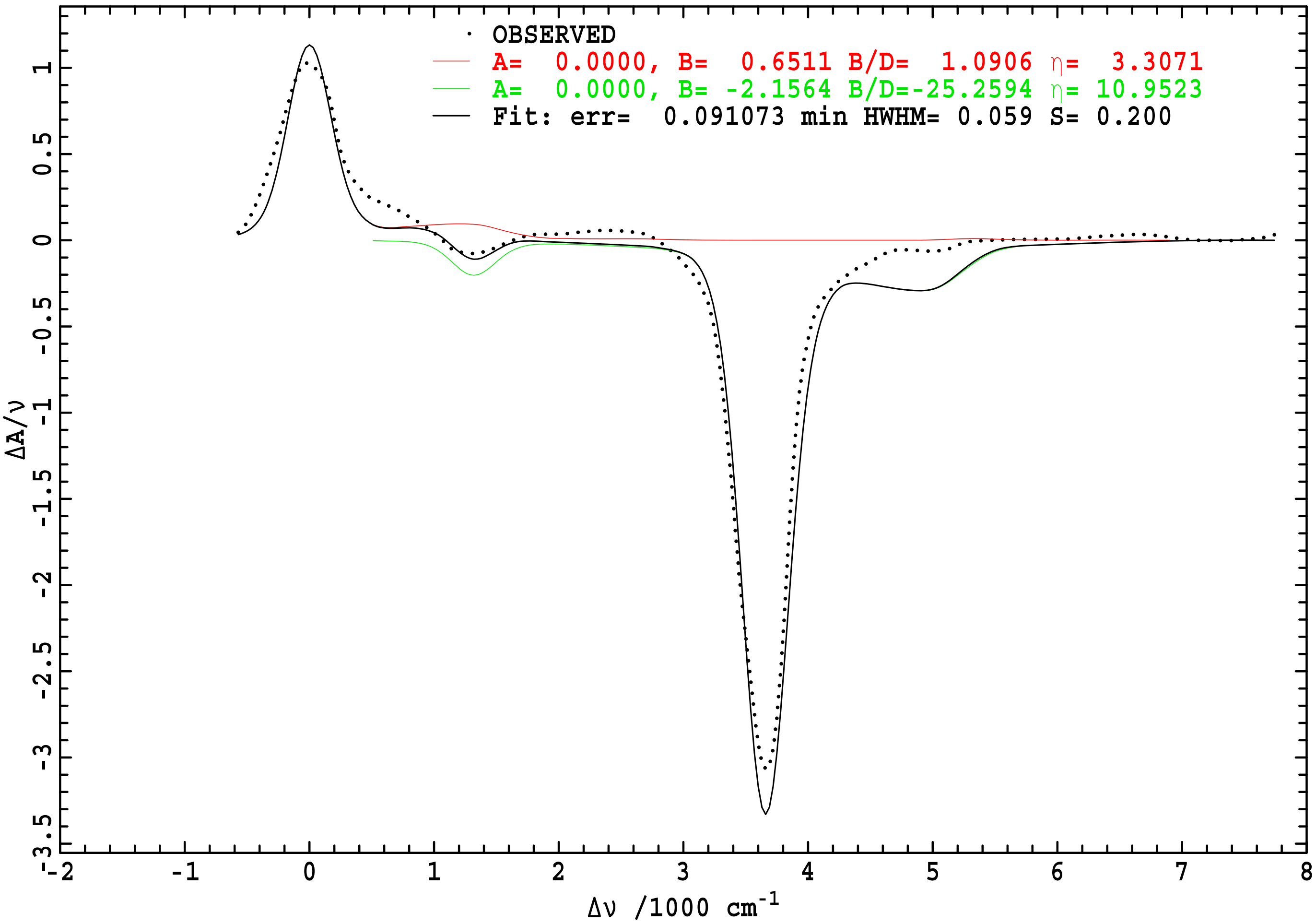


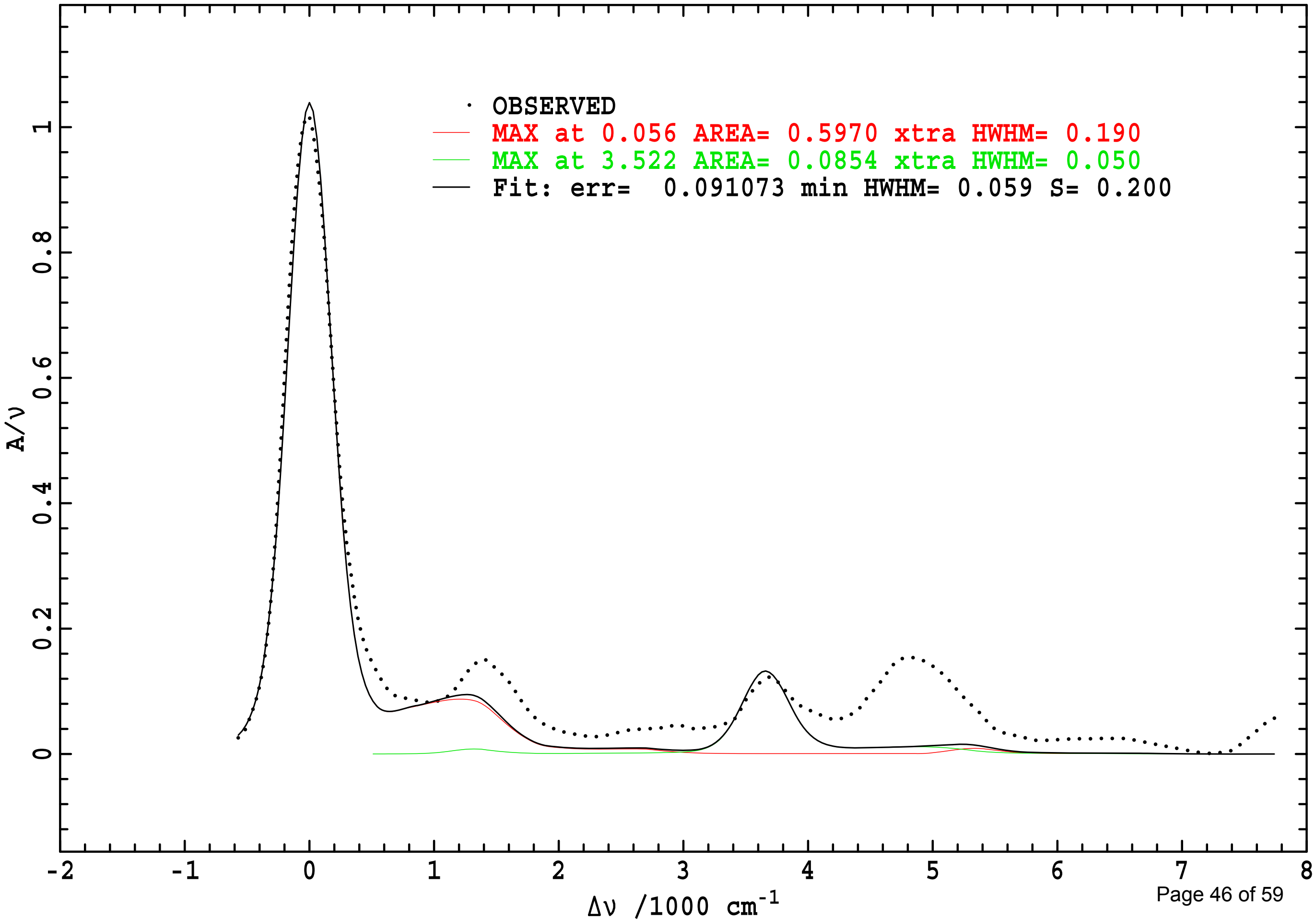

Fig. S1e: Meso-pyromethylpheophorbide-a dioxane

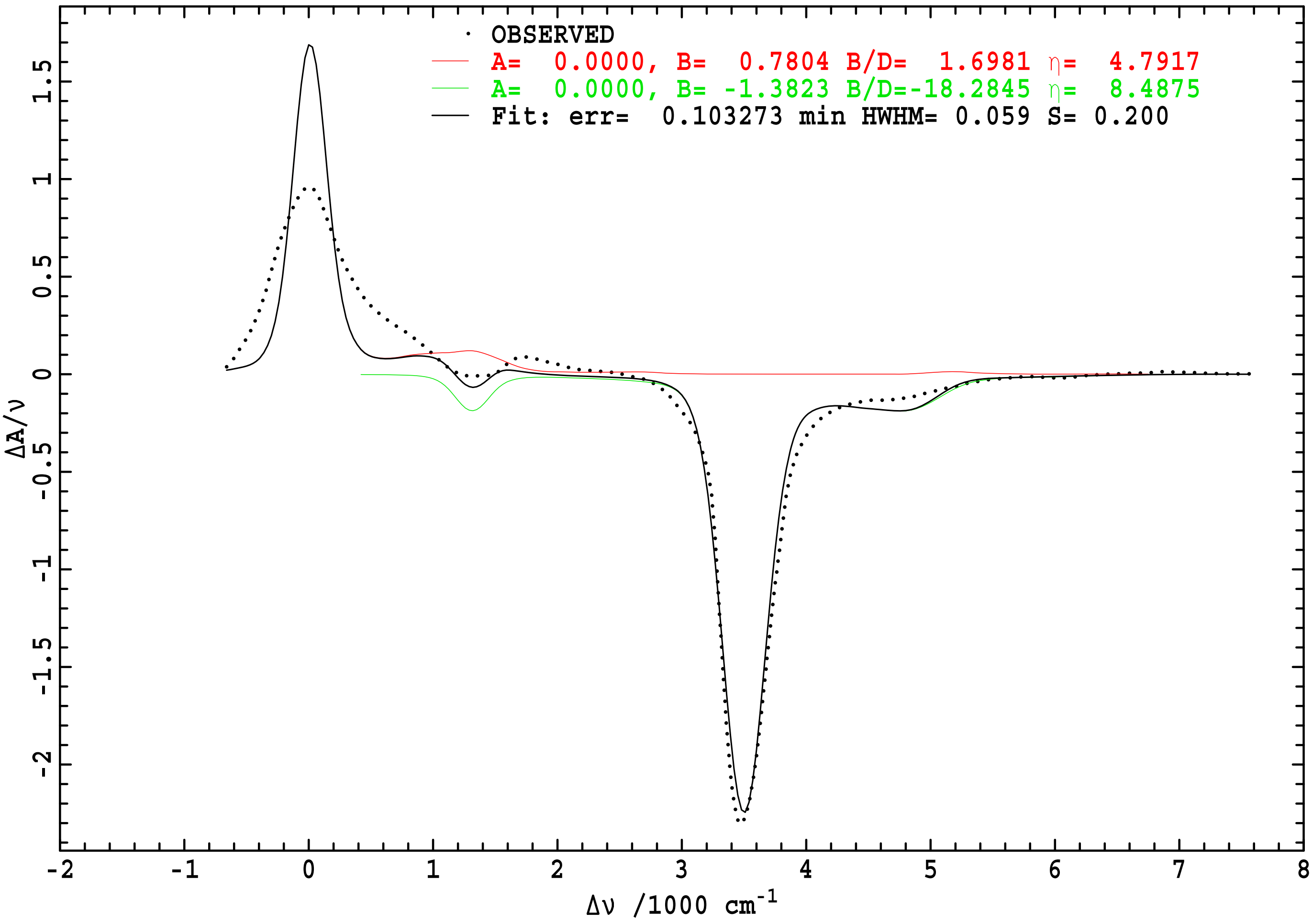


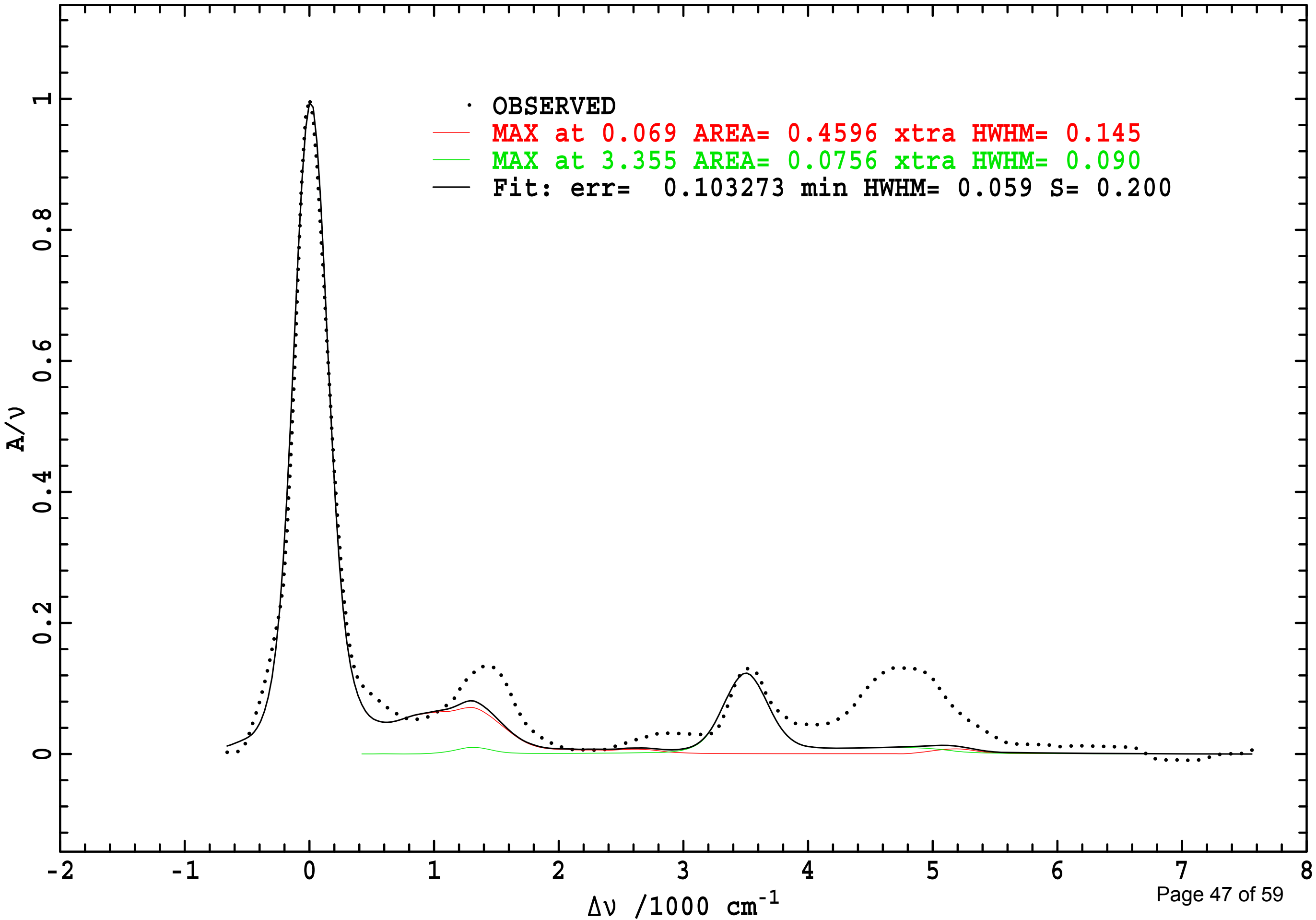

Fig. S1f: Pheo-d MeOH/EtOH 1.7 K

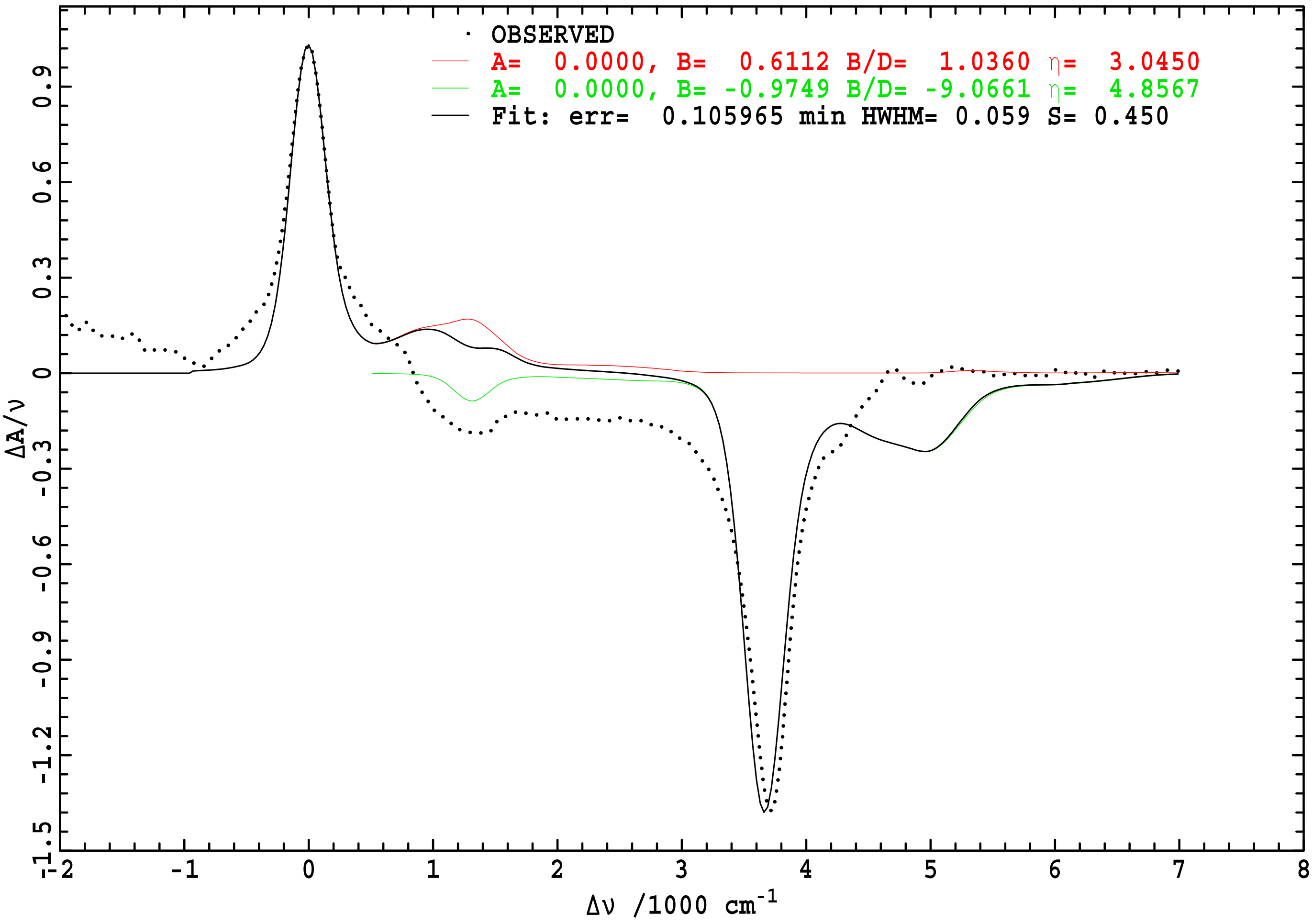


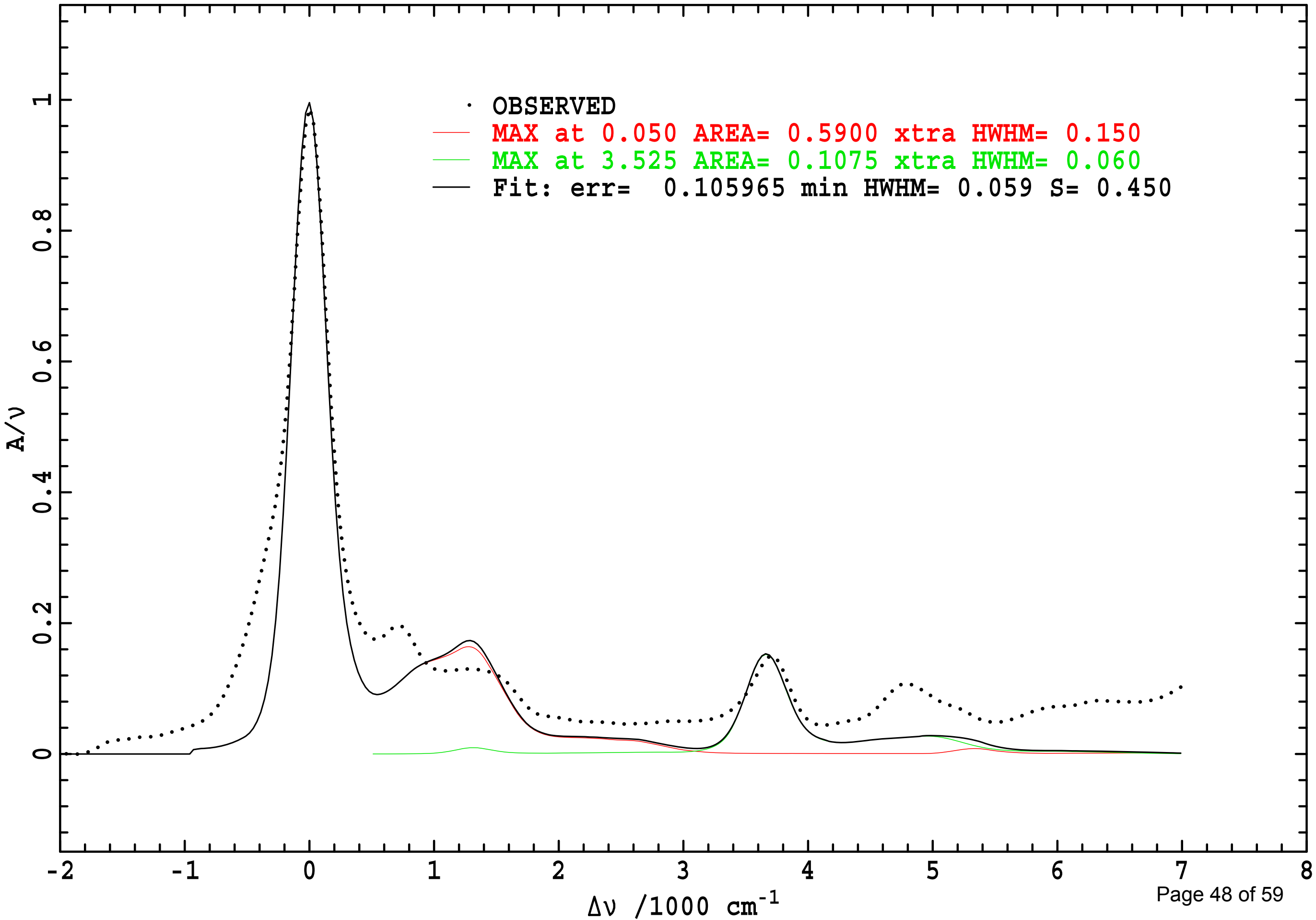

Fig. S1g: Ni(II)-Chl-a ether

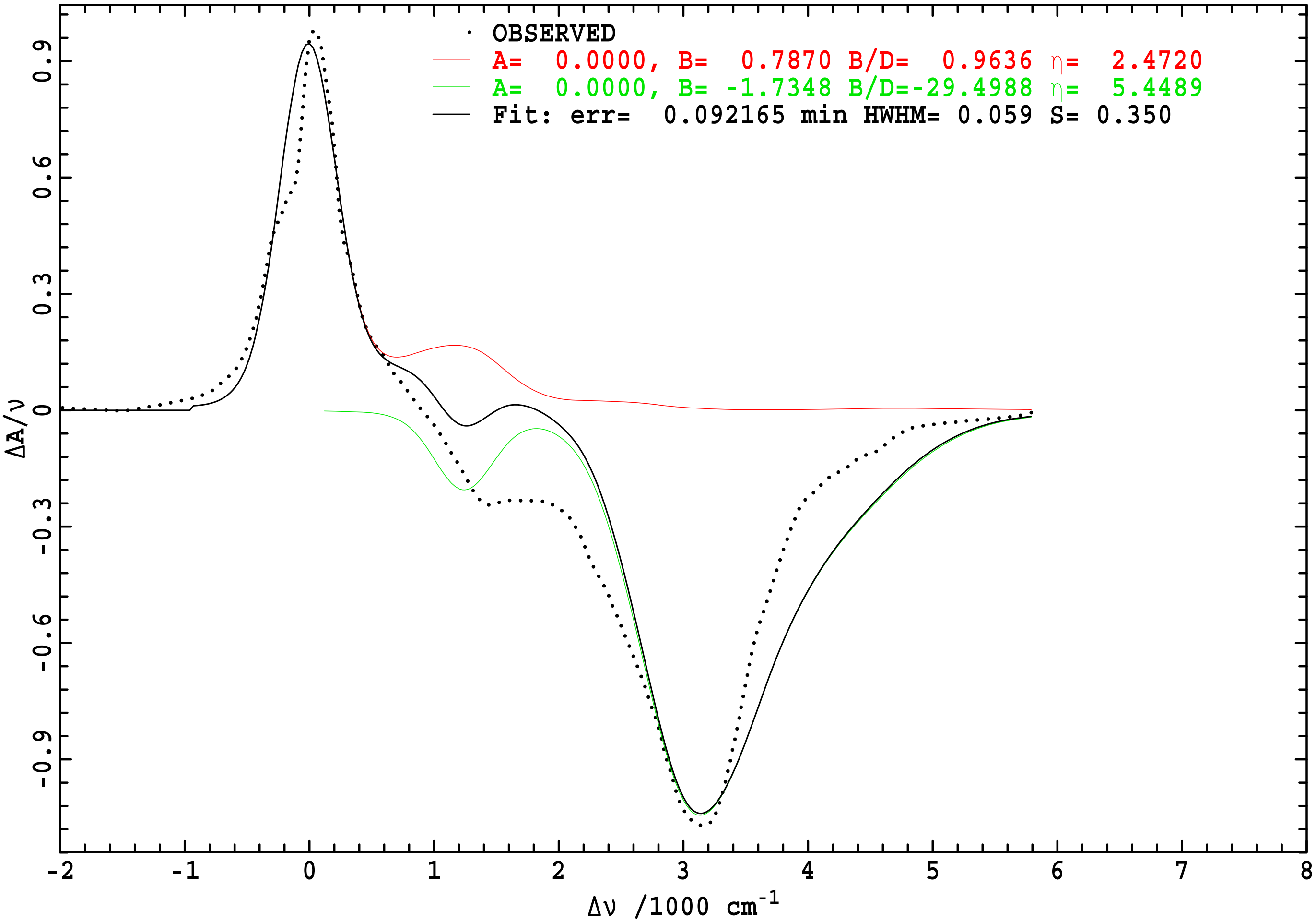


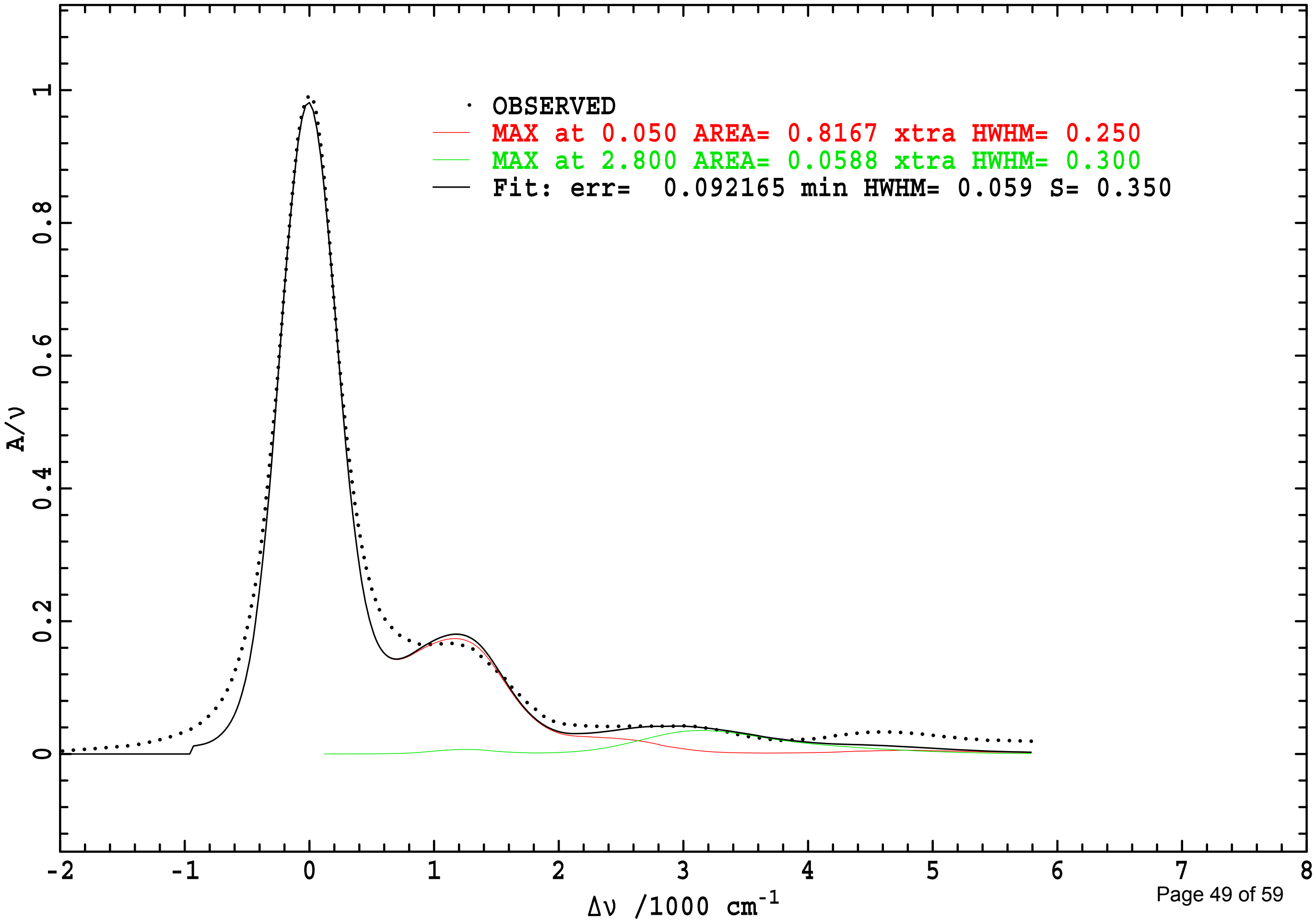

Fig. S1h: Cu(II)-Chl-a ether

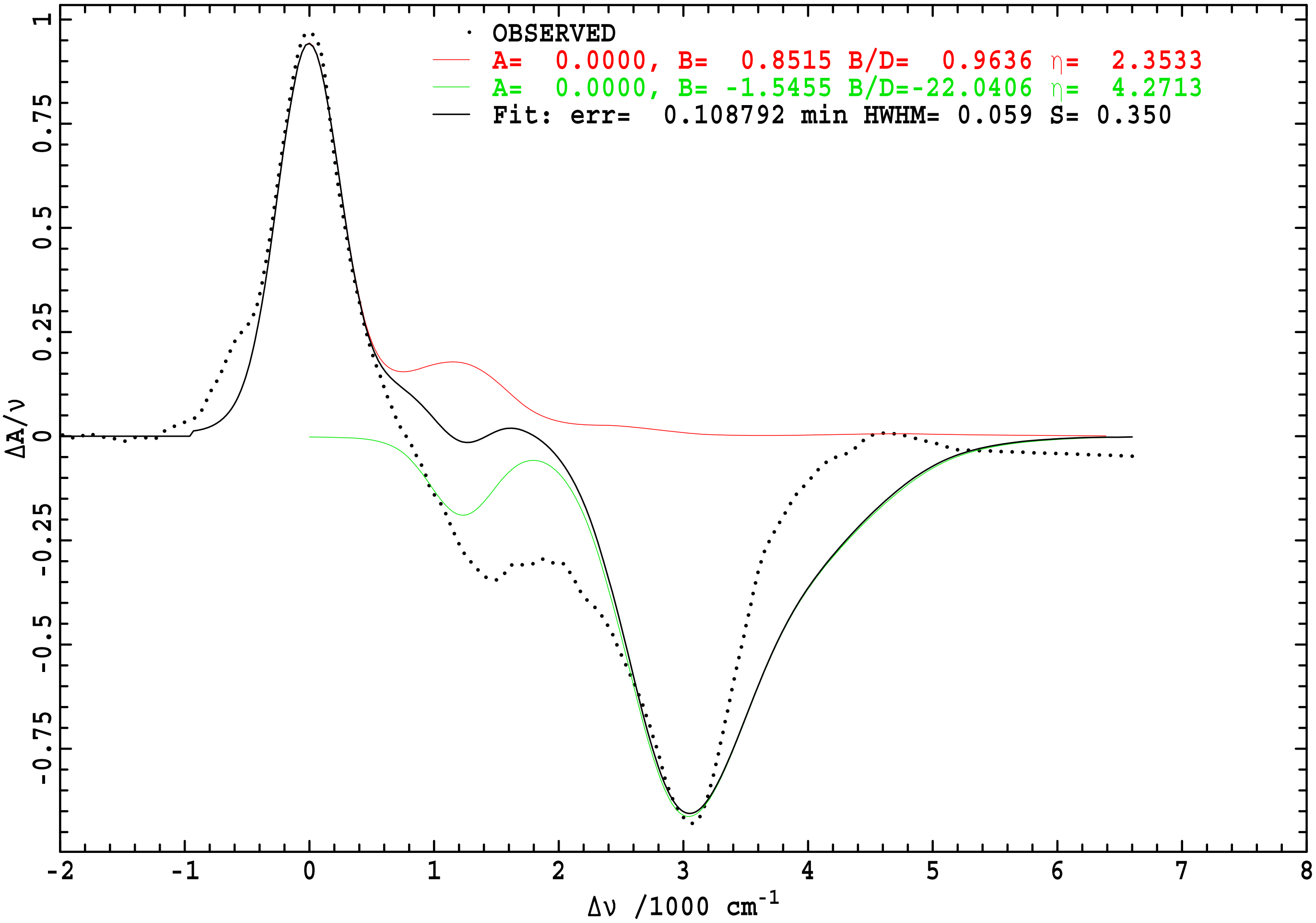


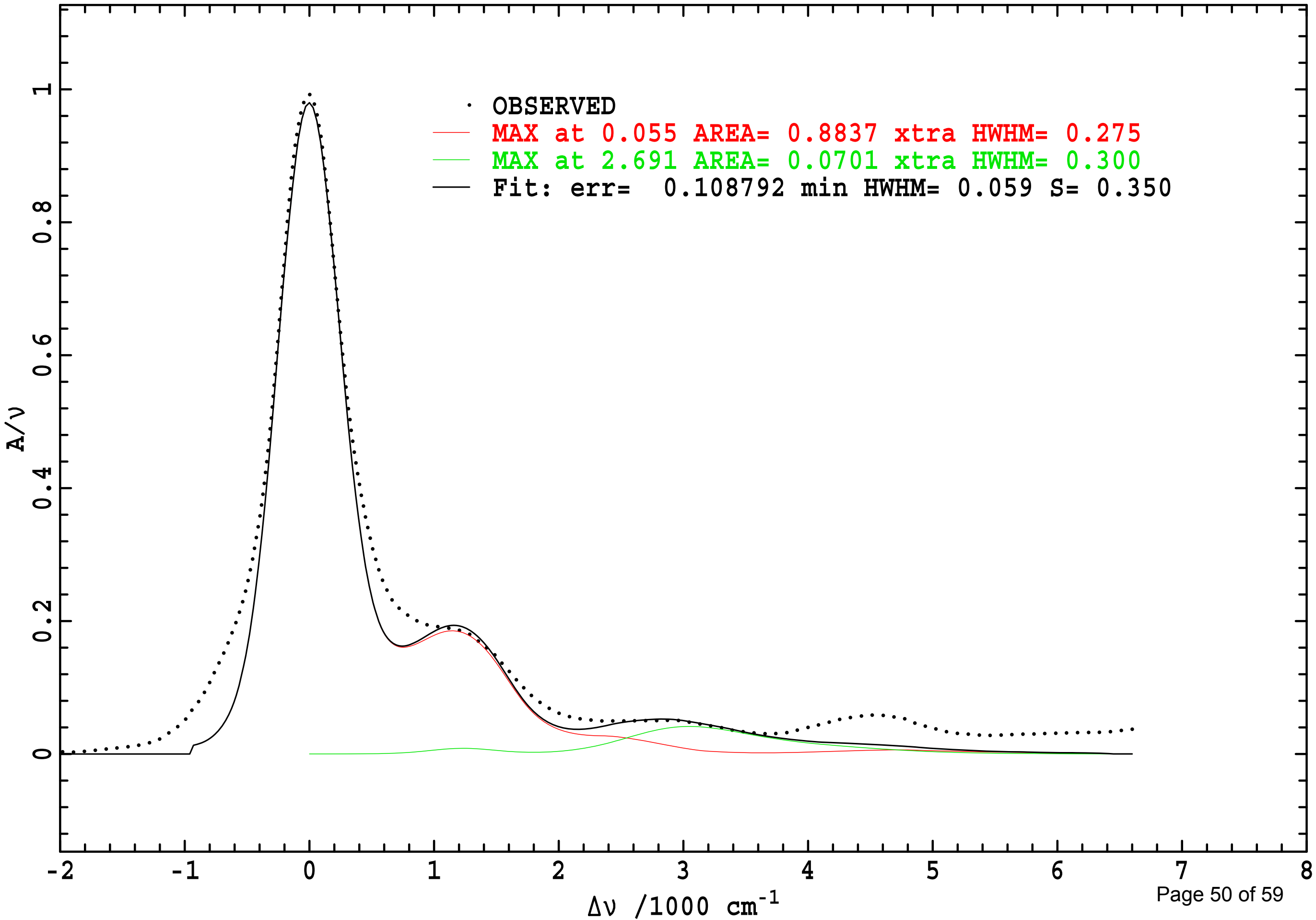

## Fig. S1i: Co(II)-Chl-a ether

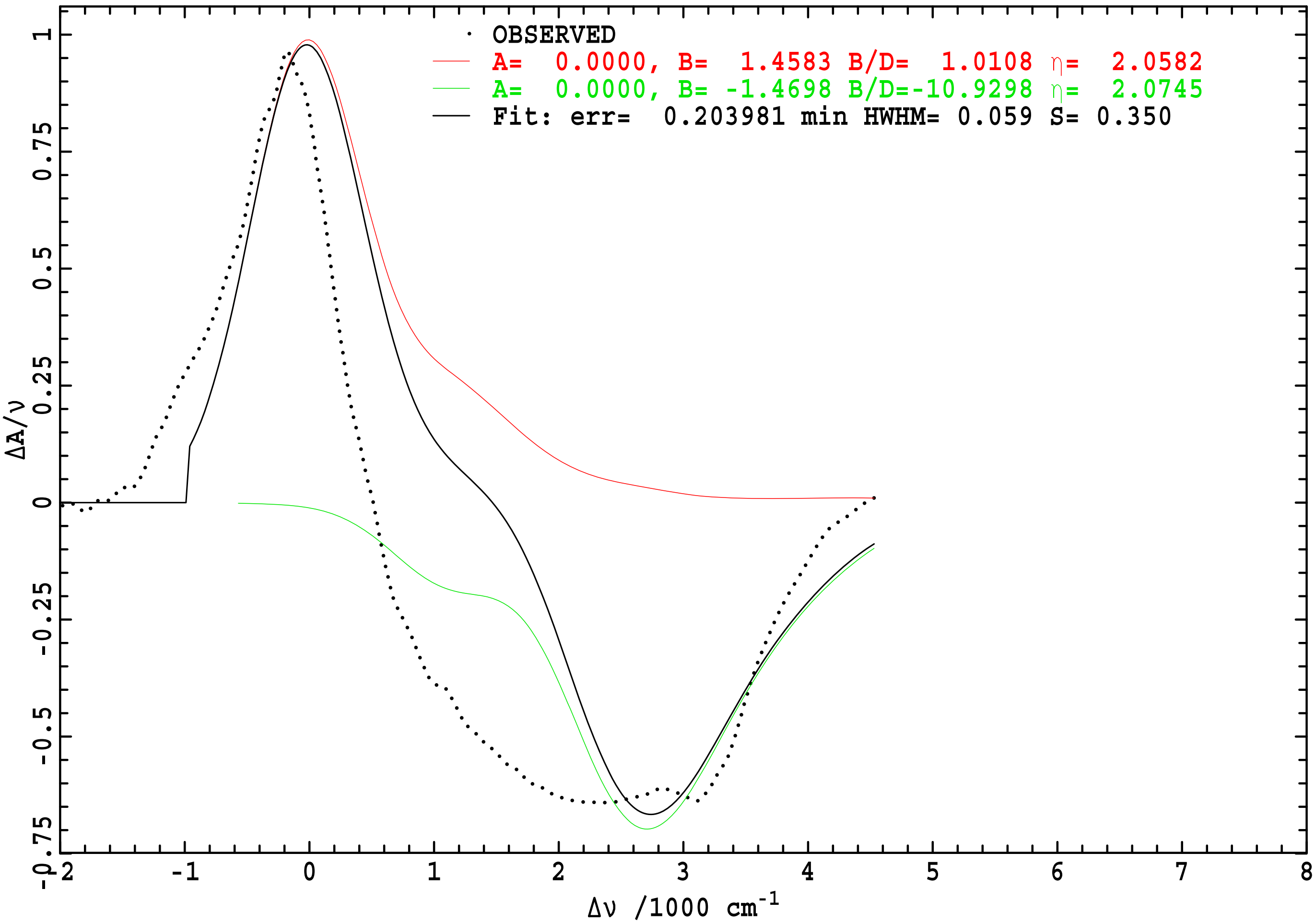


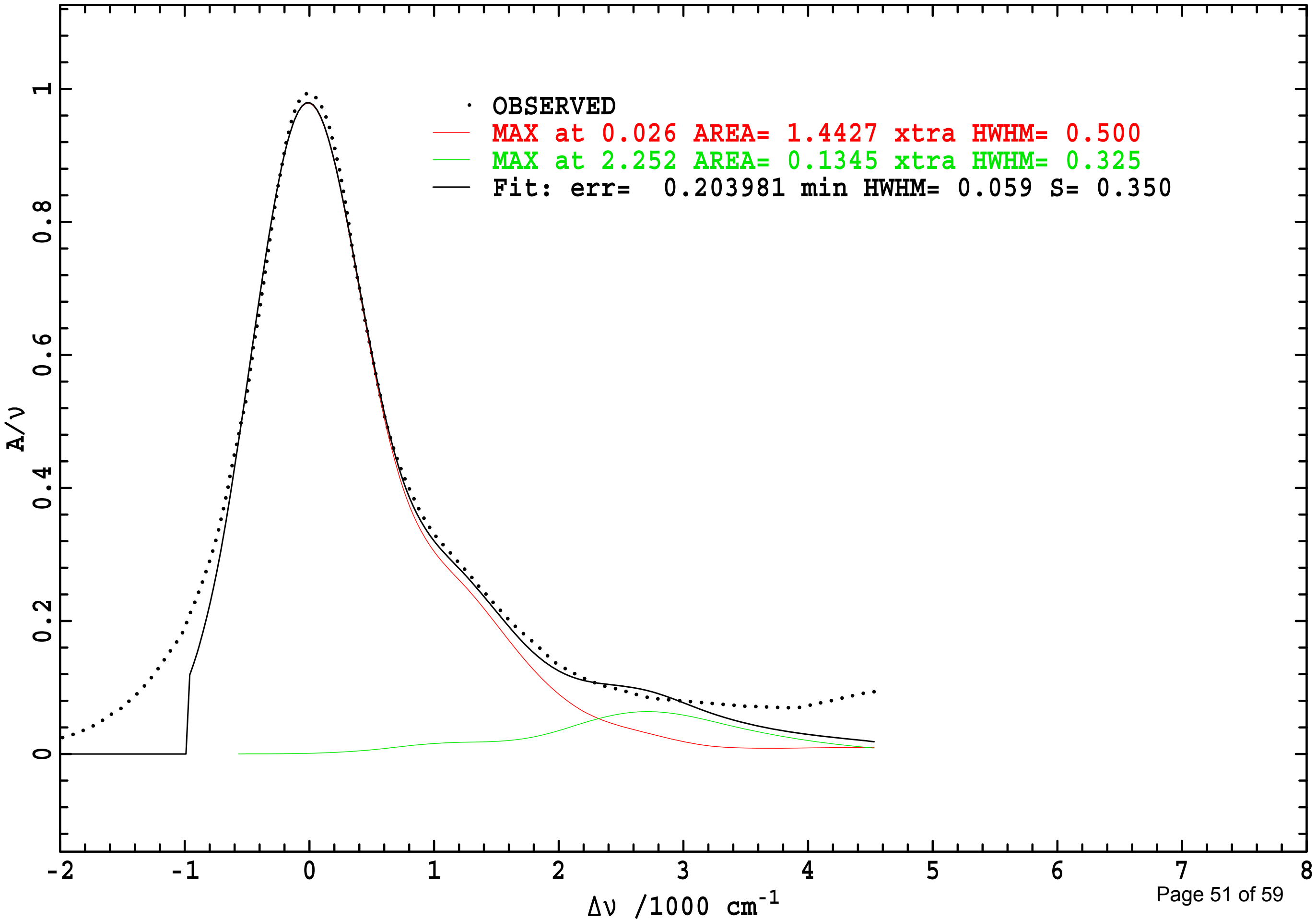

## Fig. S1j: Chl-a ether

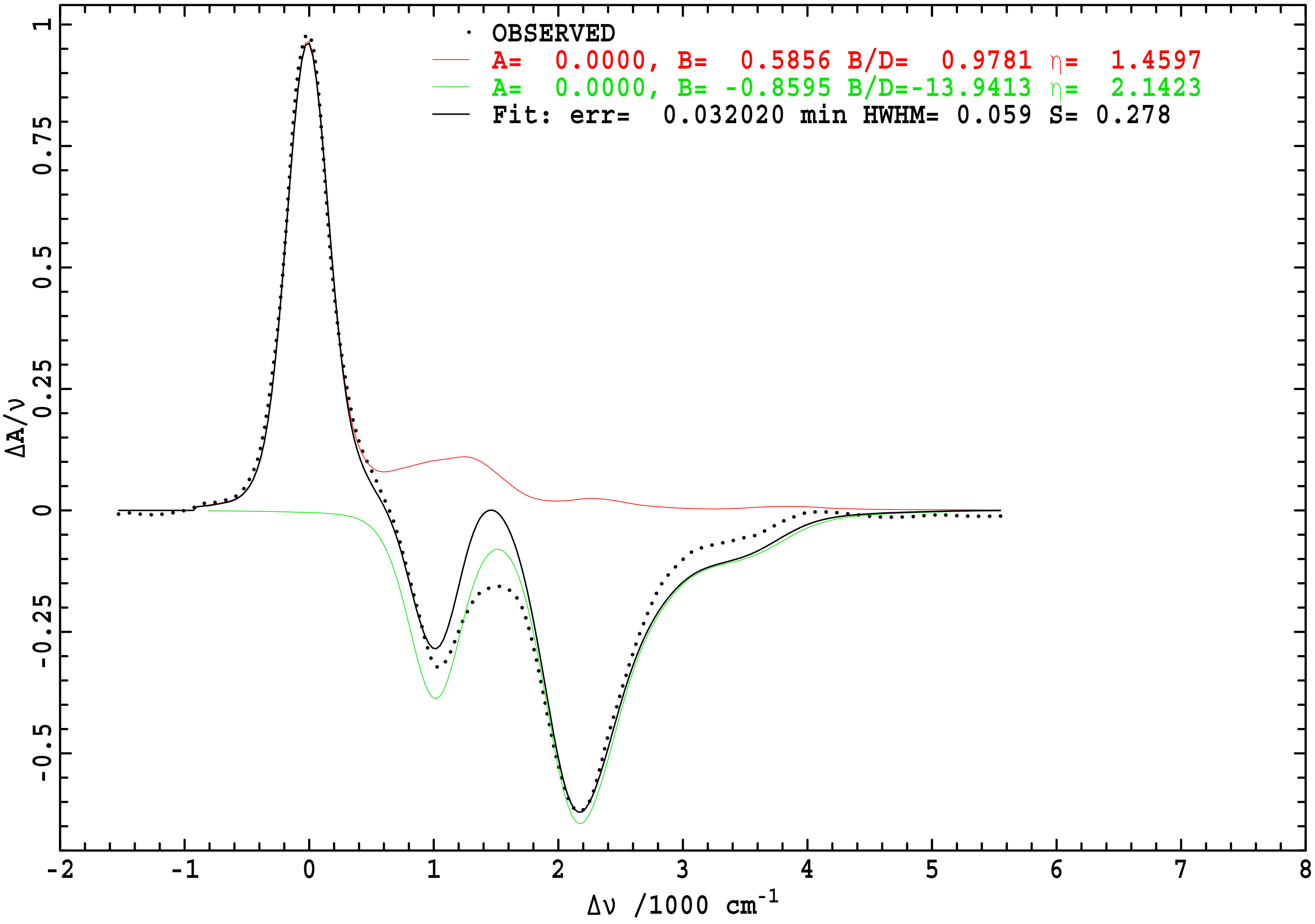


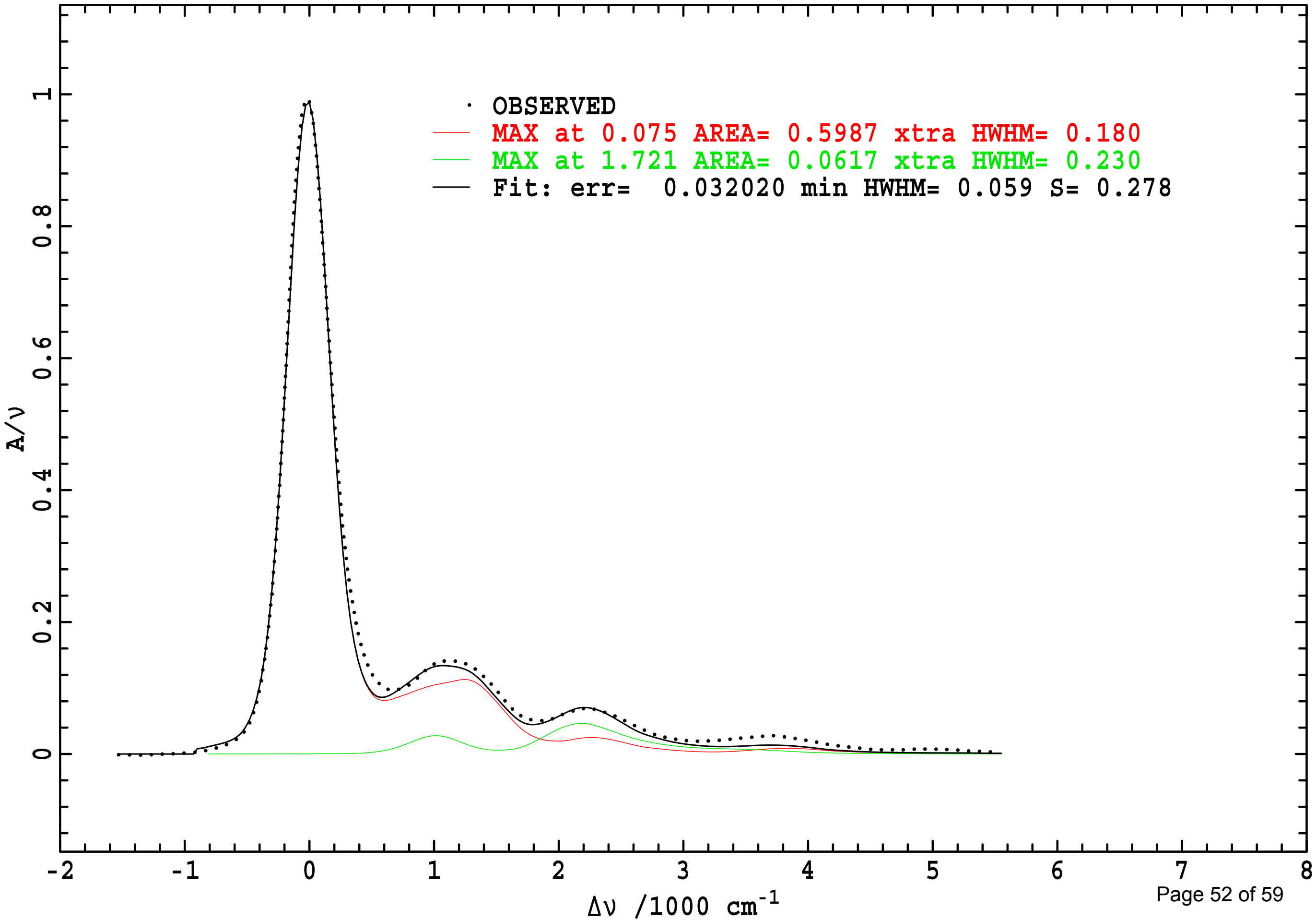

# Fig. S1k: Chl-b MBBA+EBBA+DAB

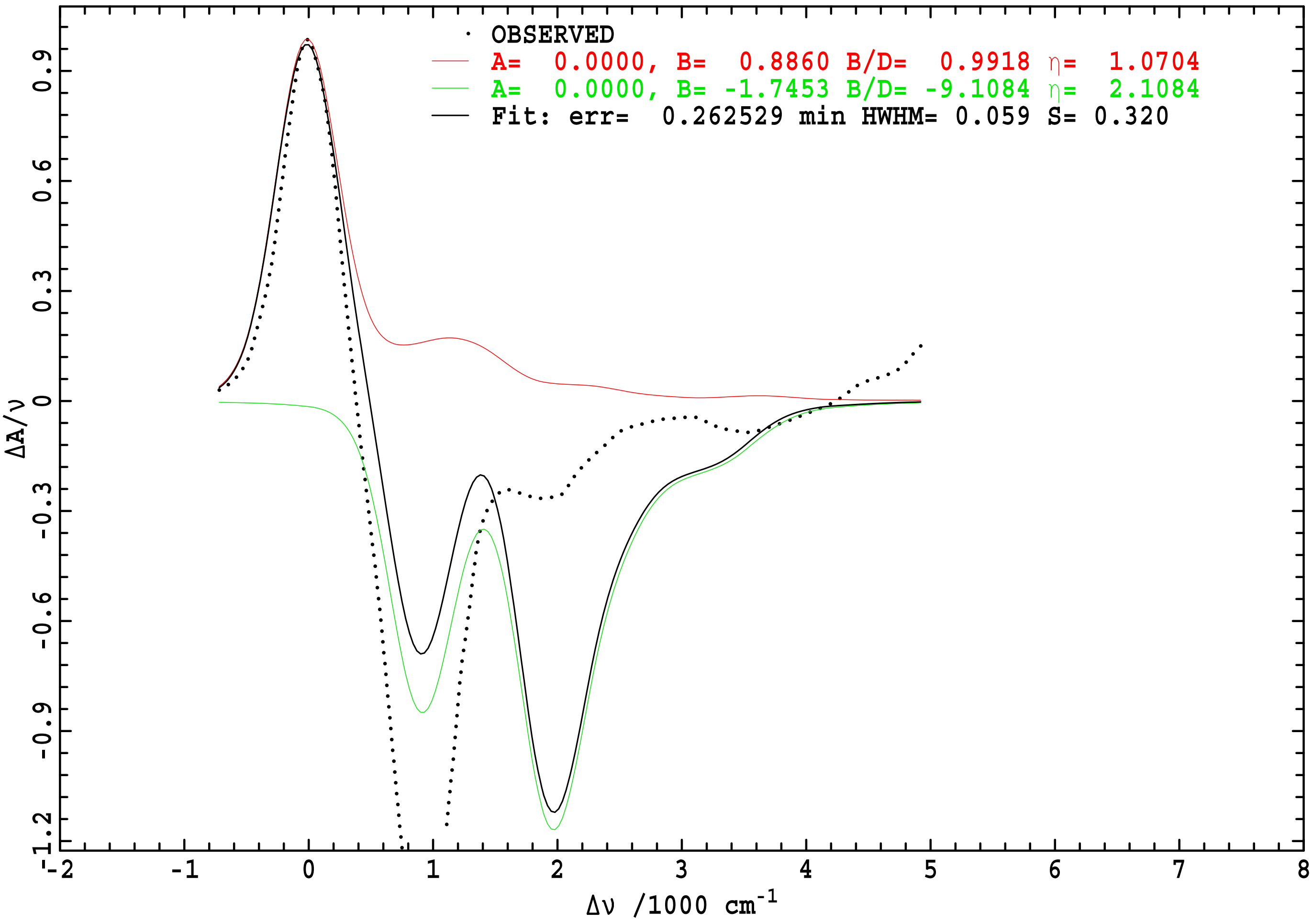


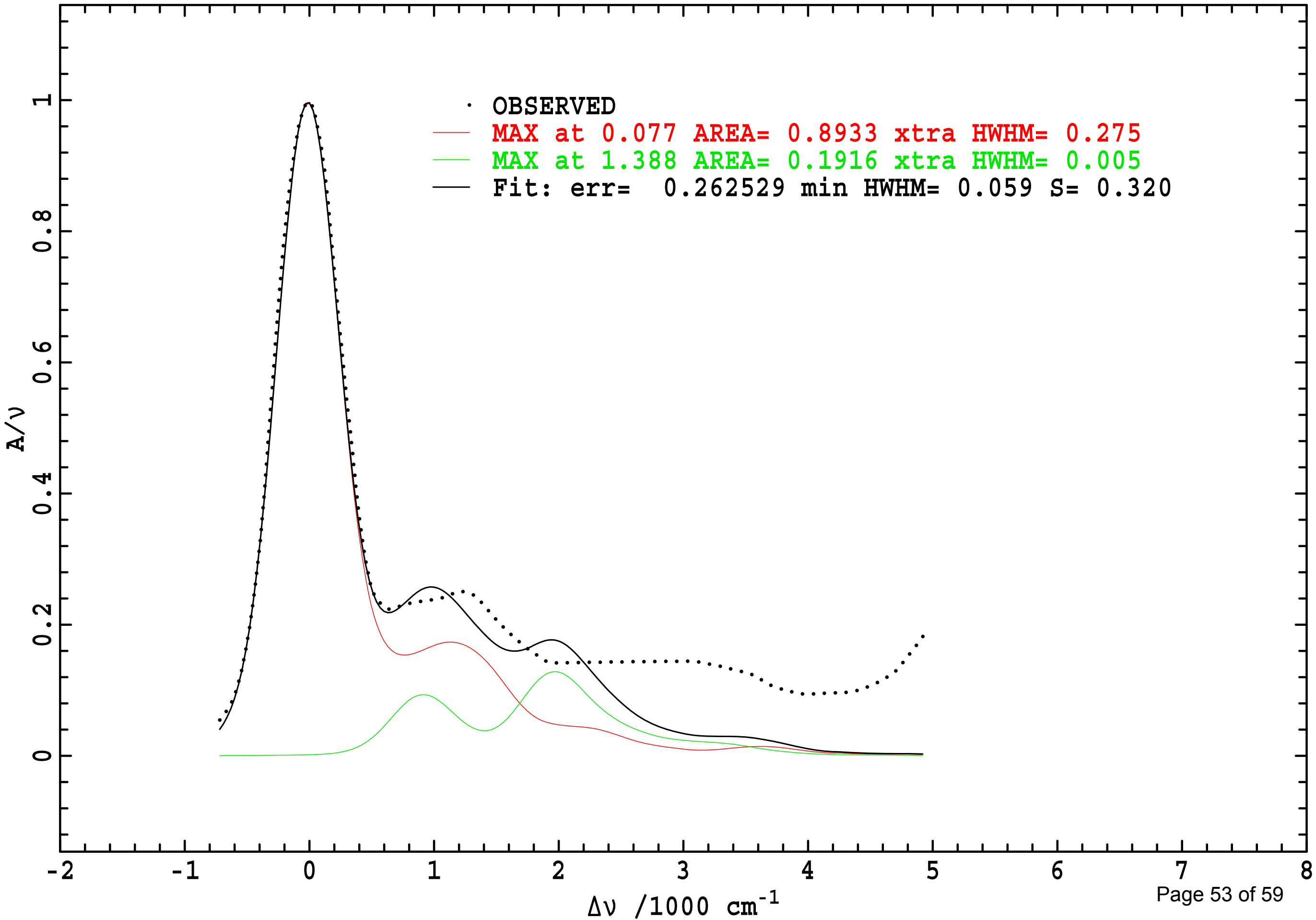

# Fig. S11: Chl-d MeOH/EtOH 1.7 K

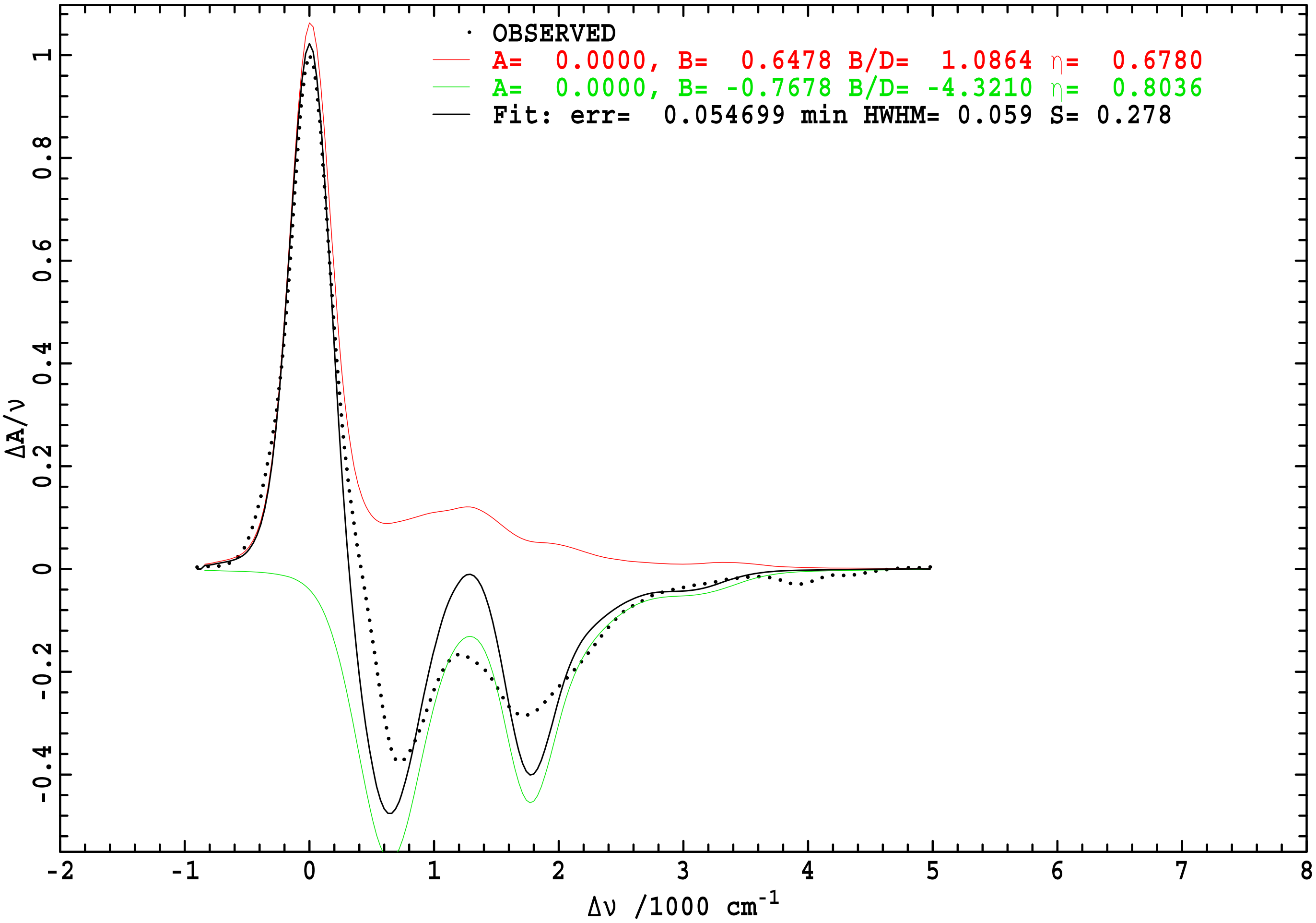


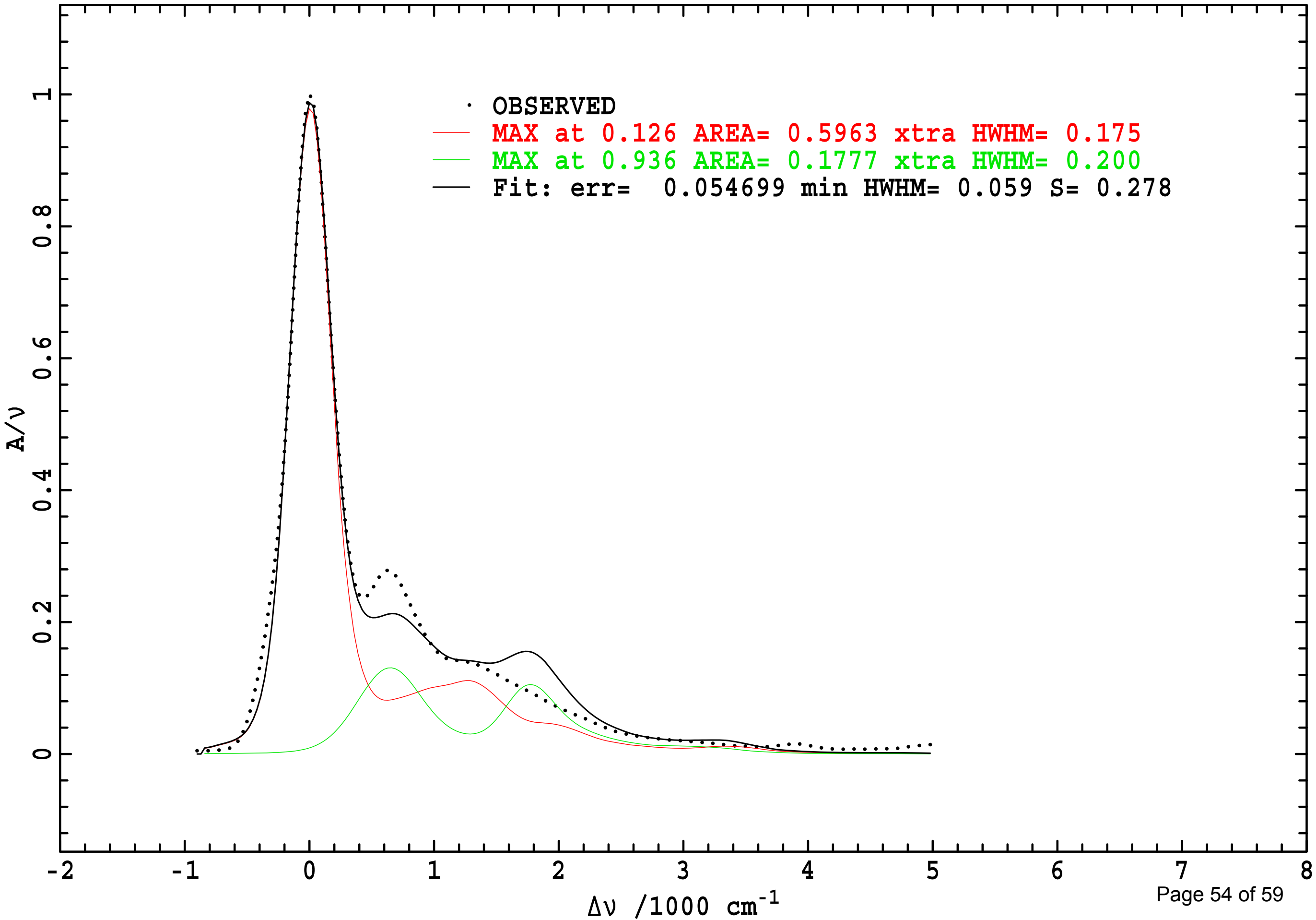

# Fig. S1m: Chl-a MeOH/EtOH 1.8 K

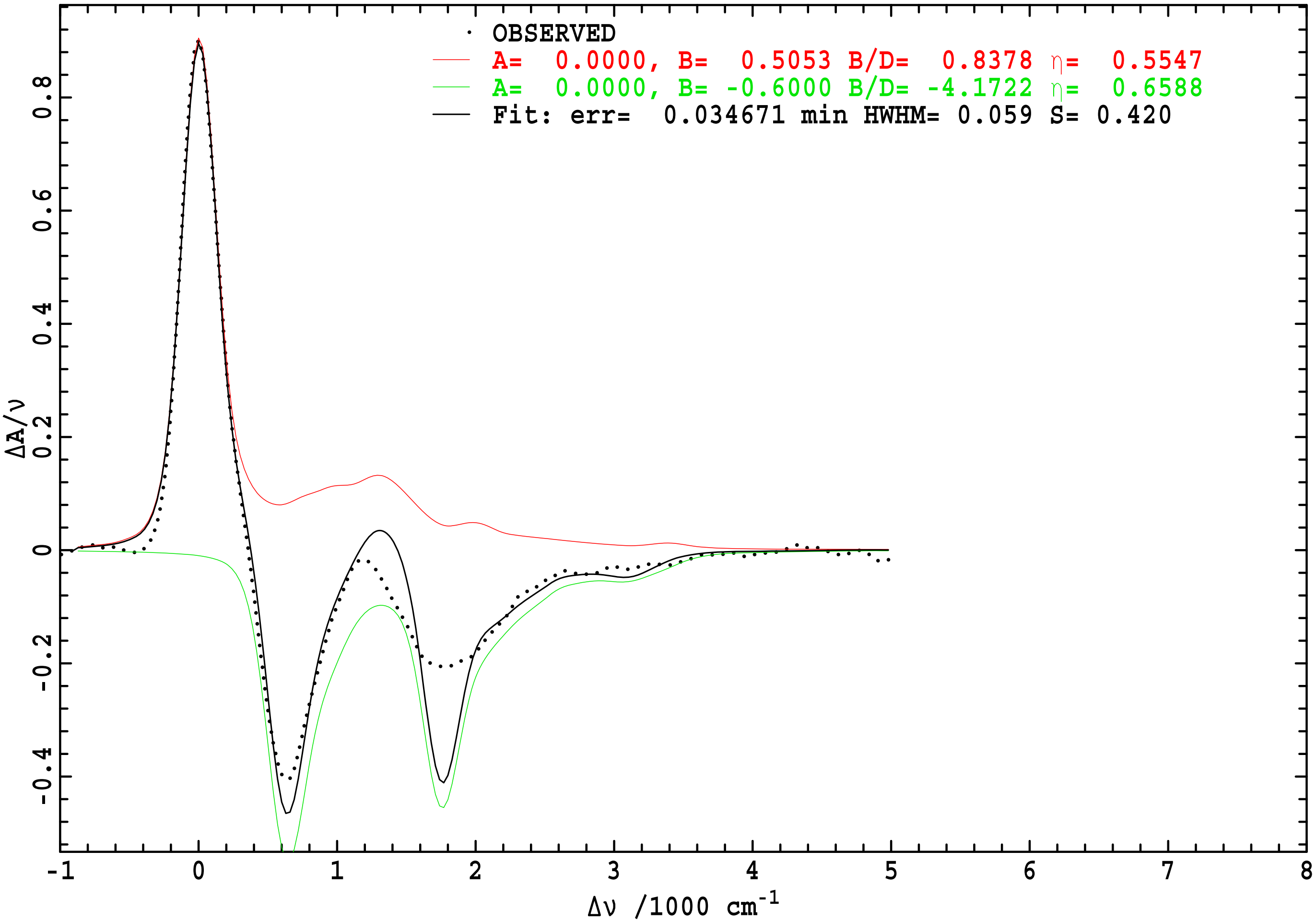


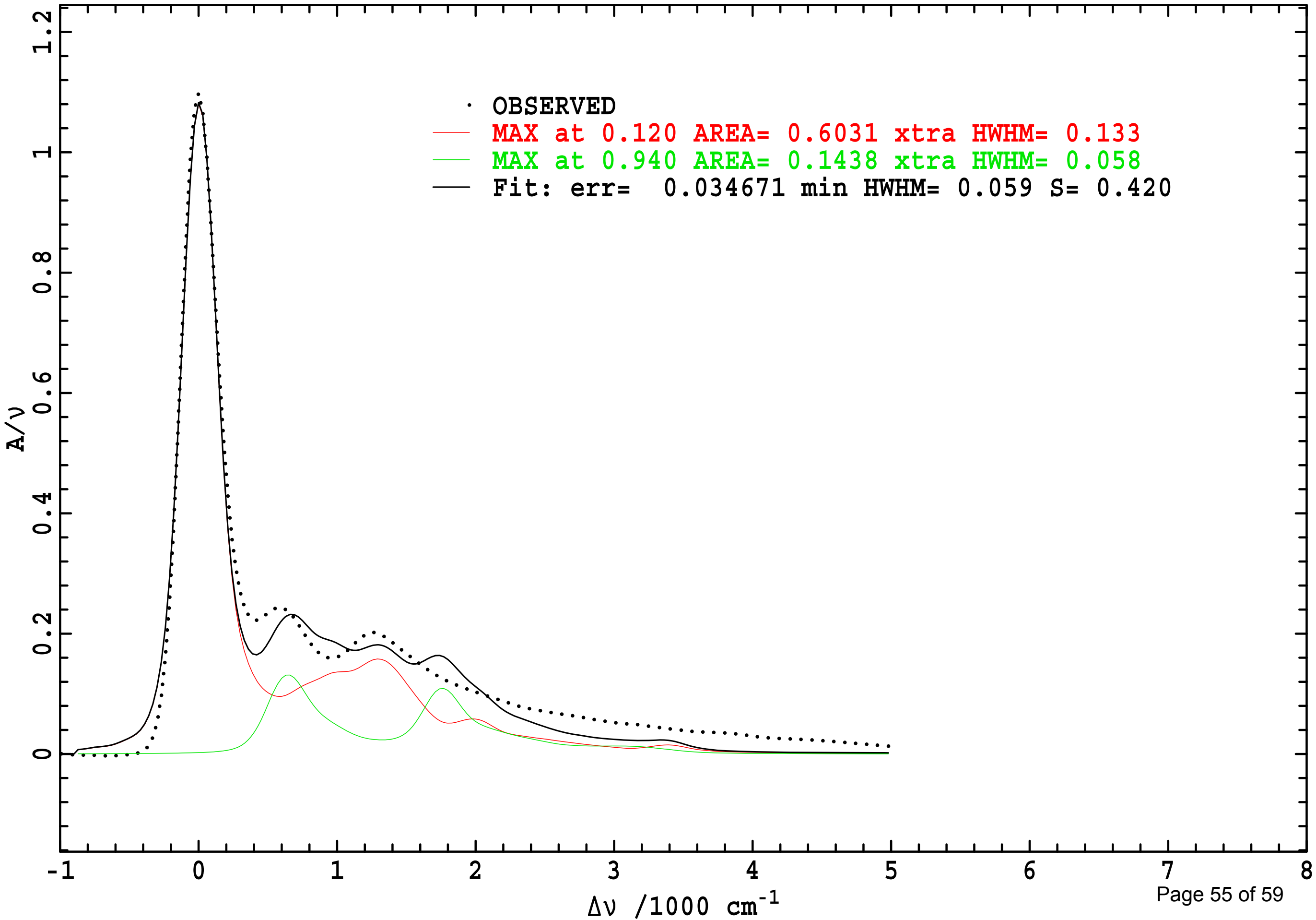

# Fig. S1n: Chl-a 1-propanol 1.8 K

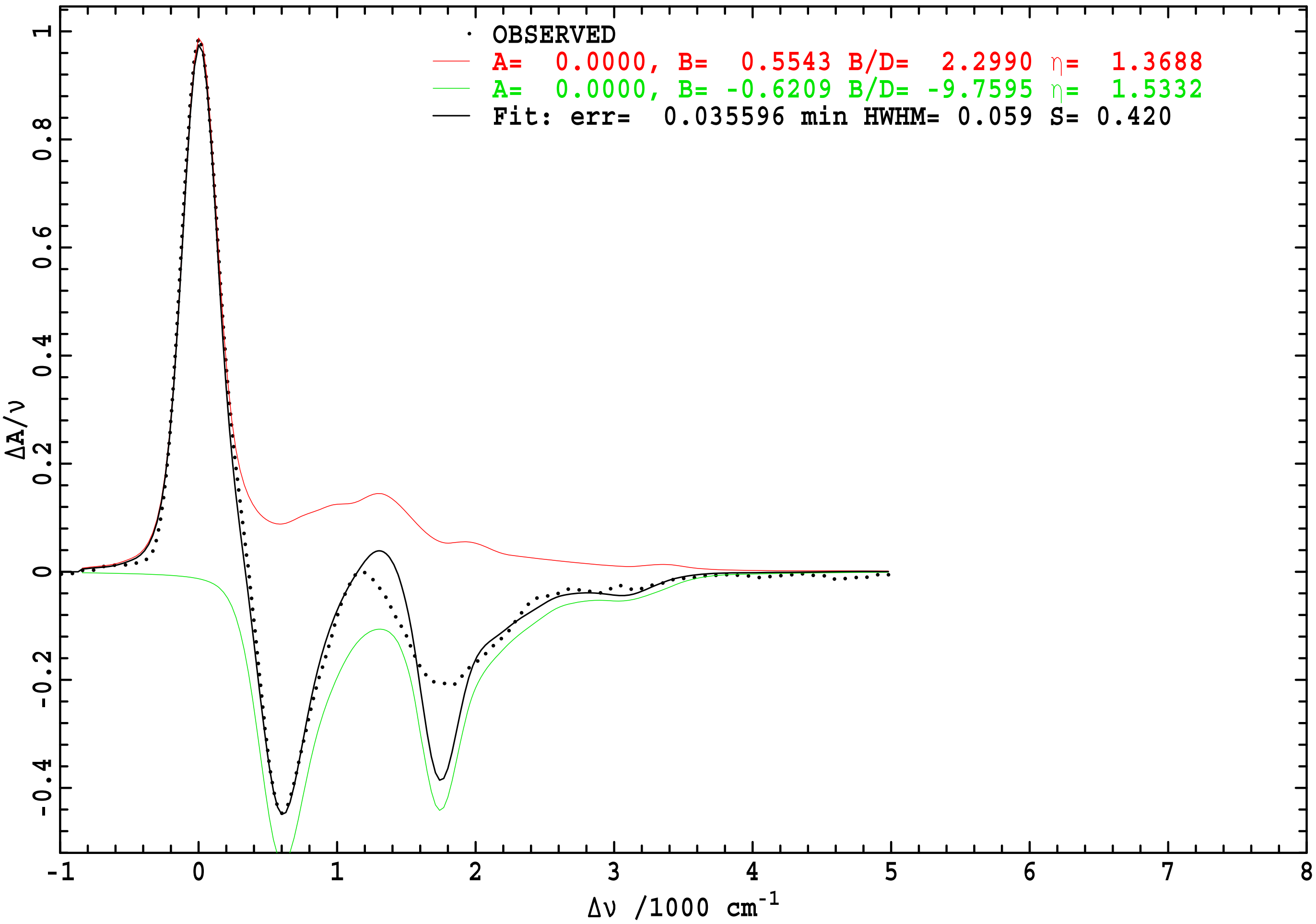


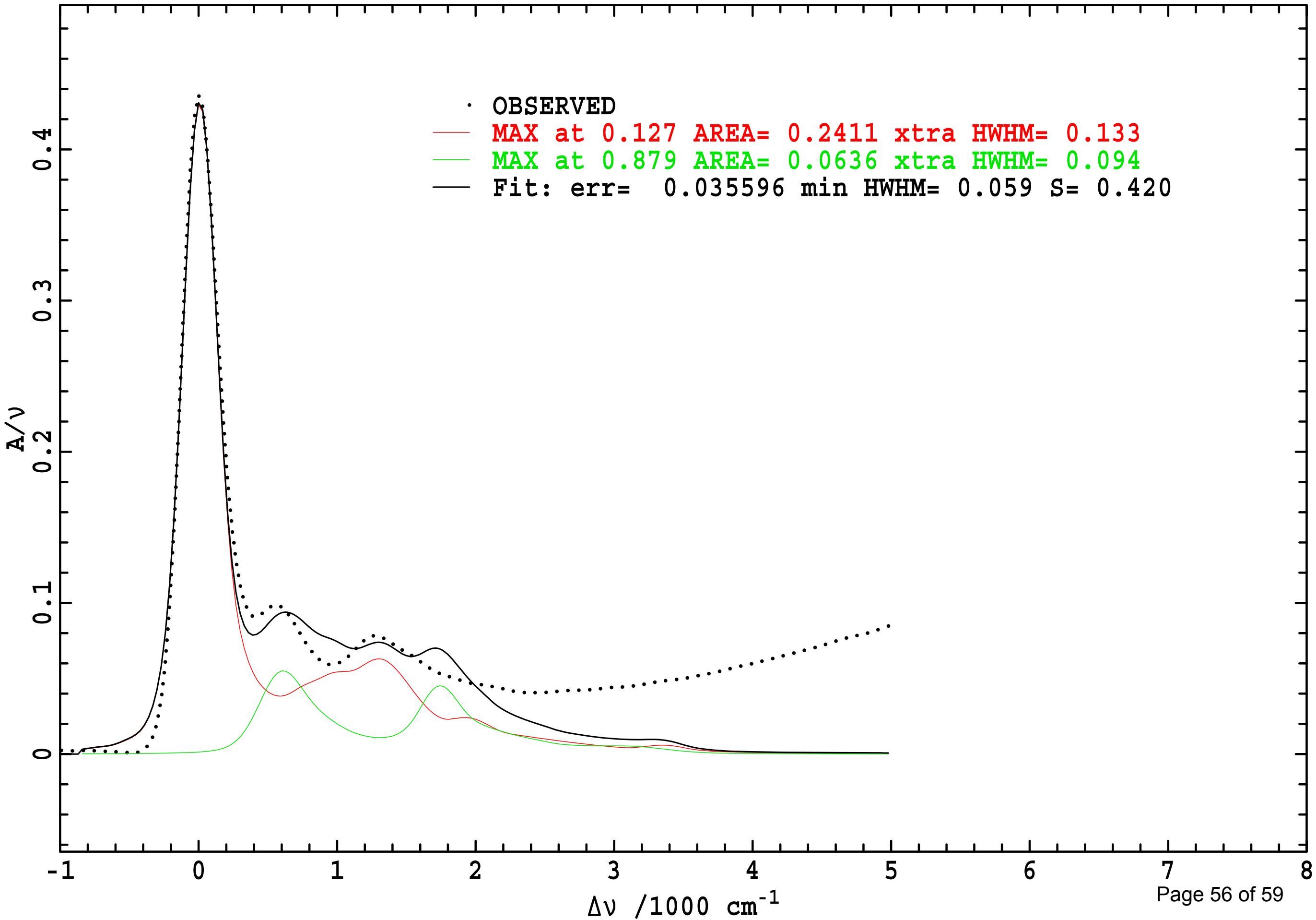

# Fig. S1o: Chl-a 1-propanol 160 K

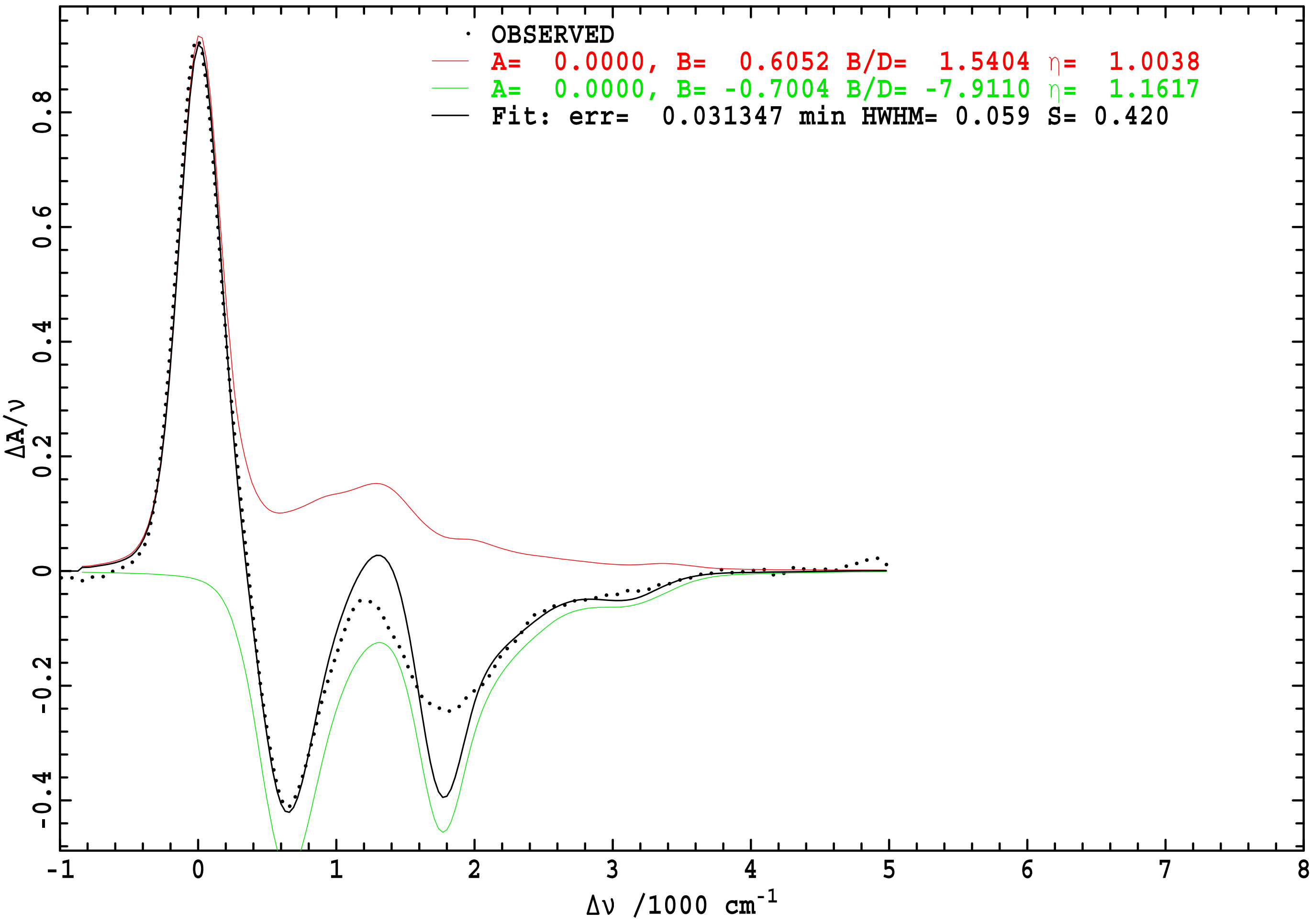


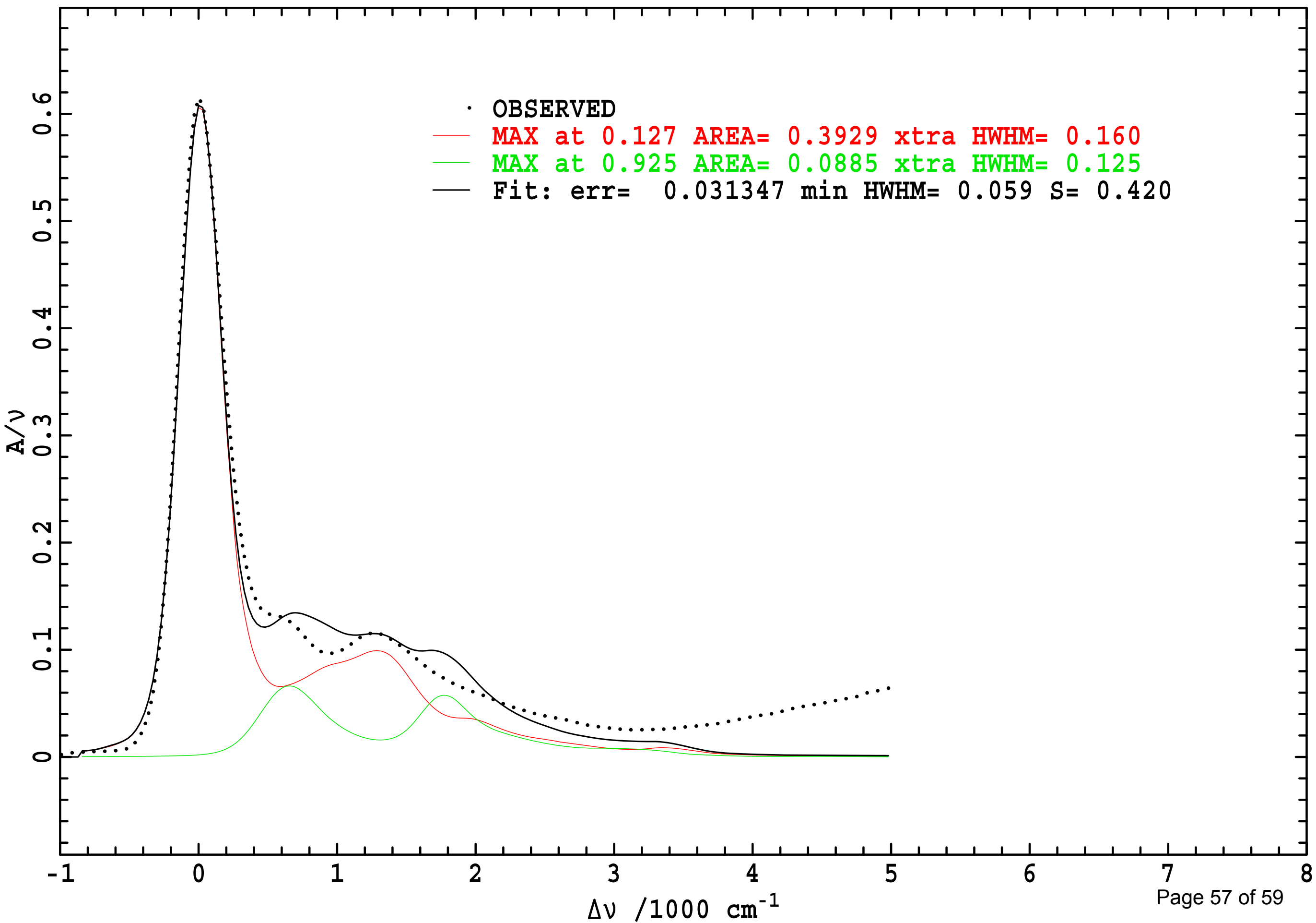

Fig. S1p: Chl-a 2-propanol 2.0 K

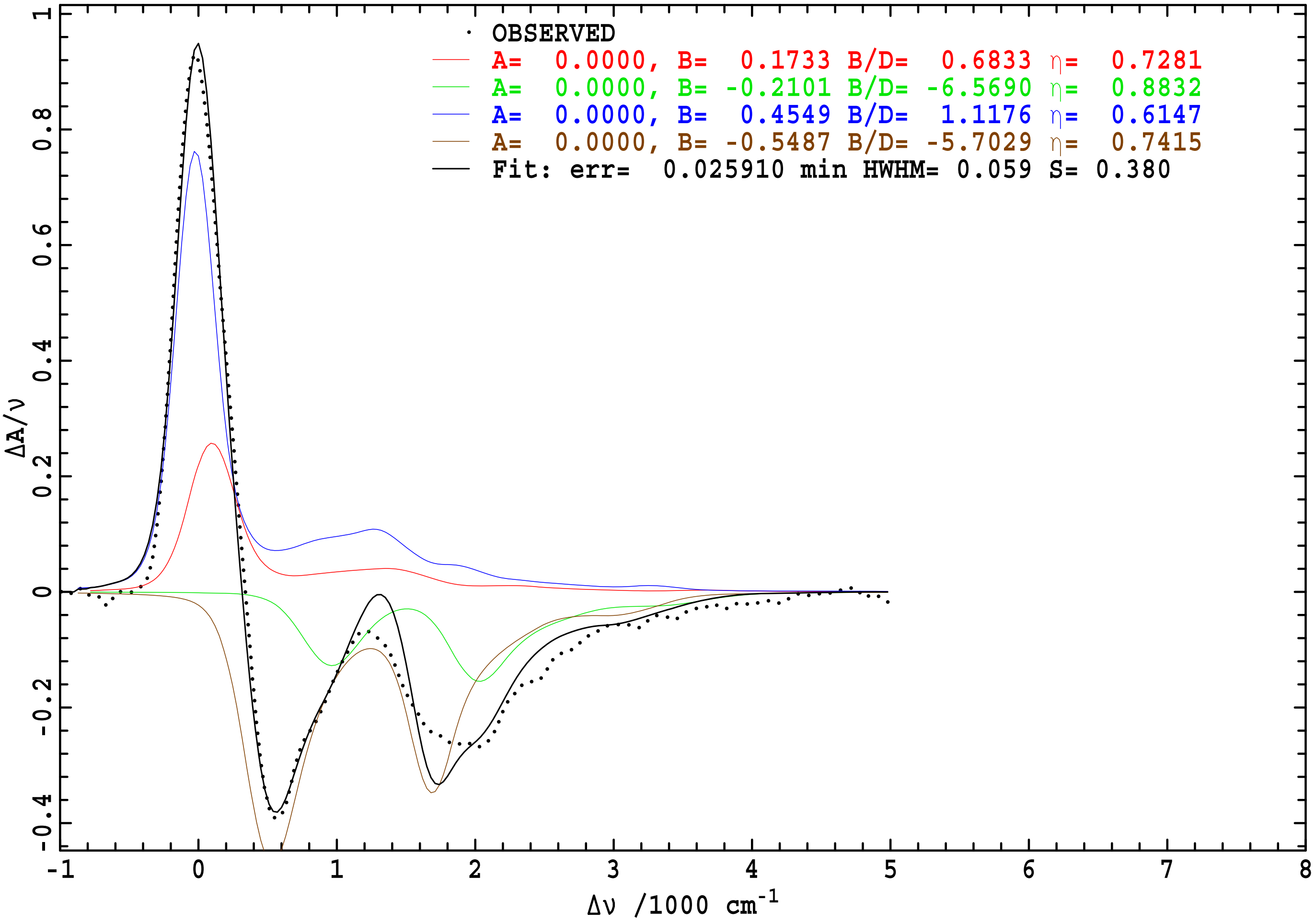


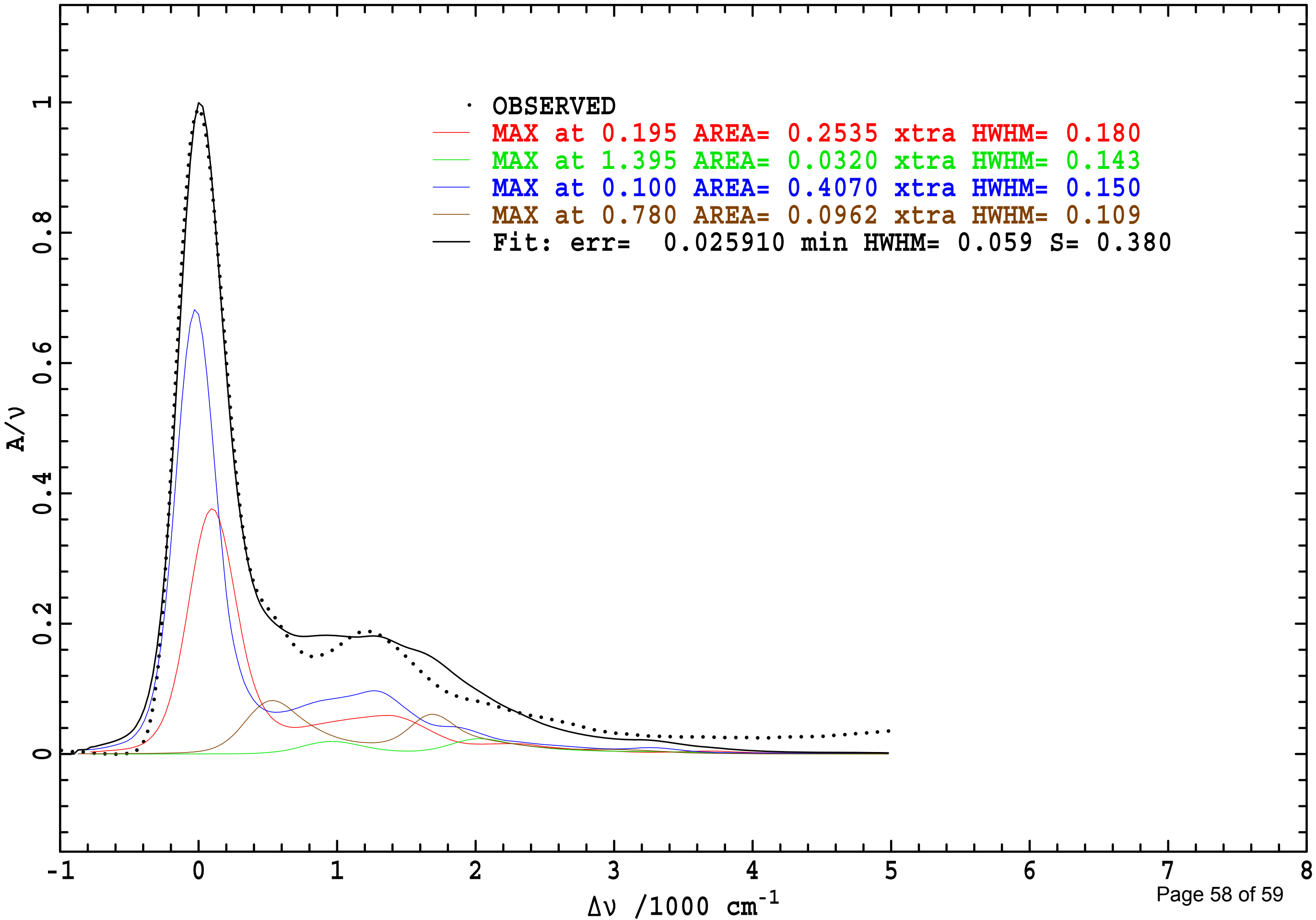

## Fig. S1q: Protochlorophyll ether

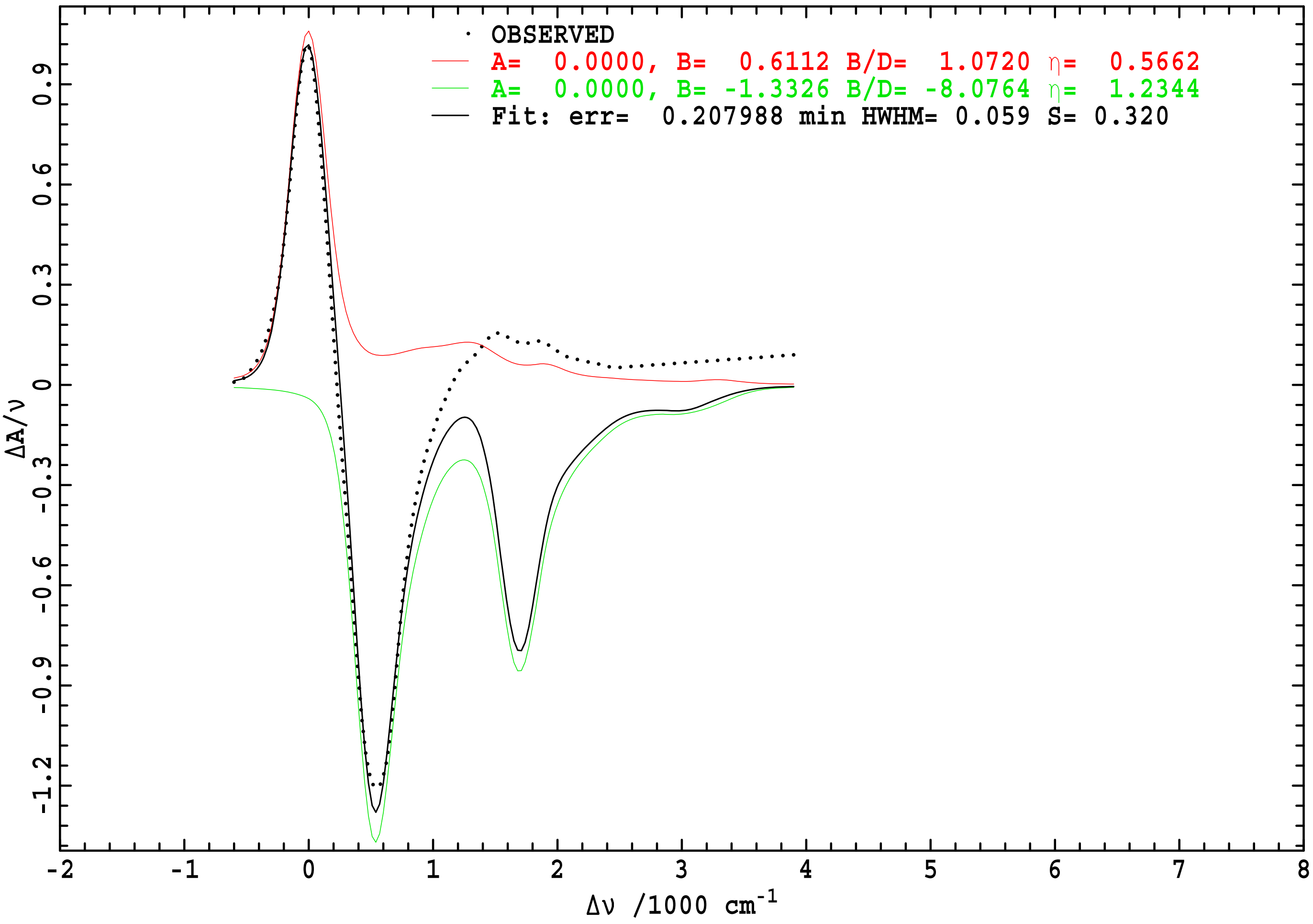


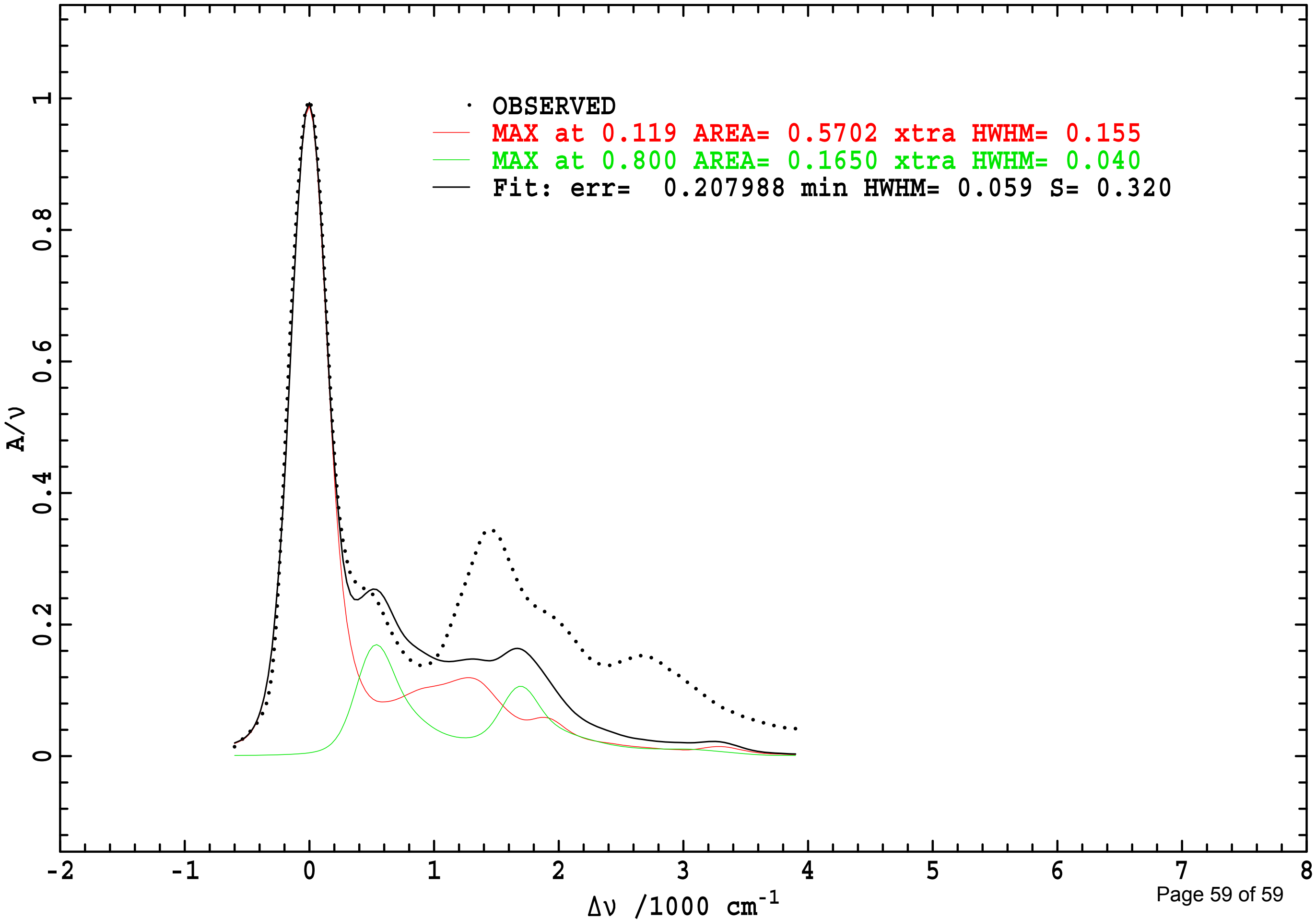

Supplement: Supplementary Information [file srep02761-s1.pdf]
